# Supplementary material for: Patient-generated data in the management of HIV: a scoping review
Source: BMJ Open. 2021 May 19;11(5):e046393. doi: 10.1136/bmjopen-2020-046393 (PMC8137219; doi:10.1136/bmjopen-2020-046393)
Supplement: Supplementary data [file bmjopen-2020-046393supp002.pdf]

Appendix B- Search terms used within the literature search strategy.

Keywords for each subject heading of 'HIV' and 'patient-generated health data' respectively were combined using the search command 'OR'. Articles yielded within each of these searches were combined using the search command 'AND' to produce the final article results.

Keywords used

Subject heading: HIV

HIV OR human immunodeficiency virus OR HIV/AIDS OR HIV-infected OR HIV-associated OR HIV-positive OR HIV positive OR HIV+ OR people living with HIV OR persons living with HIV OR HIV patient* OR HIV infection

Subject heading: Patient-generated health data

Patient generated data OR patient-generated data OR patient generated health data OR patient-generated health data OR patient generated health information OR patient-generated health information OR personal health record* OR patient portal* OR personal data sharing OR patient collected data OR patient-collected data OR personal health informati* OR personal health data OR electronic health record* OR electronic personal health record* OR electronic health data OR self-recorded health data OR self-recorded data OR self-generated data or self-reported data OR patient collated data OR patient-collected data OR patient-captured data OR patient-controlled data OR patient-reported data OR electronic record.

An example complete database search strategy, for Medline, is shown below:

# ▲	Searches	Results	Type	Actions	Annotations
1	exp HIV Infections/ or HIV/ or exp HIV Long-Term Survivors/	277245	Advanced	Display Results More ▾	
2	(HIV or human immunodeficiency virus).mp. or HIV/aids or HIV-infected.mp. or HIV-associated.mp. or HIV-positive.mp. or HIV positive.mp. or HIV+.mp. or people living with HIV.mp. or persons living with HIV.mp. or HIV patient*.mp. or HIV infection.mp. [mp=title, abstract, original title, name of substance word, subject heading word, floating sub-heading word, keyword heading word, organism supplementary concept word, protocol supplementary concept word, rare disease supplementary concept word, unique identifier, synonyms]	351883	Advanced	Display Results More ▾	
3	exp health records, personal/ or exp patient generated health data/ or exp patient portals/	1636	Advanced	Display Results More ▾	
4	(Patient generated data or patient-generated data or patient generated health data or patient-generated health data or patient generated health information or patient-generated health information or personal health record* or patient portal* or personal data sharing or patient collected data or patient-collected data or personal health informat* or personal health data or electronic health record* or electronic personal health record* or electronic health data or self-recorded health data or self-recorded data or self-generated data or self-reported data or patient collated data or patient-collected data or patient-captured data or patient-controlled data or patient-reported data or electronic record).mp. [mp=title, abstract, original title, name of substance word, subject heading word, floating sub-heading word, keyword heading word, organism supplementary concept word, protocol supplementary concept word, rare disease supplementary concept word, unique identifier, synonyms]	28979	Advanced	Display Results More ▾	
5	1 or 2	391186	Advanced	Display Results More ▾	
6	3 or 4	29523	Advanced	Display Results More ▾	
7	5 and 6	489	Advanced	Display Results More ▾	
