# Supplementary material for: Patient-generated data in the management of HIV: a scoping review
Source: BMJ Open. 2021 May 19;11(5):e046393. doi: 10.1136/bmjopen-2020-046393 (PMC8137219; doi:10.1136/bmjopen-2020-046393)
Supplement: Supplementary data [file bmjopen-2020-046393supp003.pdf]

Appendix C- Data extraction table of included articles

**Studies concerning a hypothetical digital platform for PGData collection**							
**Authors (date)**	**Location**	**Participants**	**Study design**	**Length of study**	**Study aims and summary**	**Digital platform summary**	**Measures**
Bussone (2018, Study One of three)	England, UK	N = 16 people living with HIV<br><br>Age: range 18-57 years, M = 39.87<br><br>Gender: 8 male, 8 female<br><br>Ethnicity: 1 White British, 1 White other, White/Black African, 8 Black African, 2 Latin, 1 Asian Pakistani, 1 White/Black/Caribbean, 1 Mixed other<br><br>Orientation: 5 homosexual, 9 heterosexual, 1 bisexual, 1 asexual	Qualitative, individual semi-structured interviews of approximately 30 minutes	N/A	• **Aim**: to understand how people living with HIV monitor their personal health data<br>**Topics addressed:** participants' current self-management behaviours and issues surrounding current, previous or desired means of tracking personal health information	N/A as no specific digital platform used	Verbal feedback from participants regarding self-tracking of their personal health information

Marent, Henwood & Darking (2018)	Brighton (UK), Lisbon (Portugal), Barcelona (Spain), Antwerp (Belgium) and Zagreb (Croatia)	N = 160<br>• 97 people living with HIV<br>• 63 HCPs working in HIV care<br>-40 doctors<br>-10 nurses<br>-4 psychologists<br>-4 pharmacists<br>-2 social workers<br>-2 nutritionists<br>-1 sexologist<br><br>Age (of people living with HIV): range = 23-78 years<br><br>Gender (people living with HIV): 78 male<br><br>Ethnicity: authors report that people living with HIV had 14 different nationalities, with 13 identifying as belonging to a migrant community<br><br>Orientation (people living with HIV): 65 gay or lesbian, 26 heterosexual, 3 bisexual, 3 other.	Qualitative, 14 recorded workshops and 22 semi-structured interviews	January-June 2016	• **Aim**: to determine perceptions of, and advantages and concerns surrounding, the development of a mobile app for HIV management<br>• **Topics addressed:** perceived challenges and barriers to use of a mobile app for HIV management, current mobile app usage and perceived useful features of a mobile app for HIV management	A hypothetical mobile app was discussed.	Verbal feedback from participants regarding factors related to a hypothetical mobile app for HIV management

Nokes et al. (2013)	New York, USA	N = 100 people living with HIV/AIDS<br><br>Age: M= 48.5<br><br>Gender: 94 male<br><br>Orientation: unreported<br><br>Ethnicity: 7 Asian/Pacific Islander, 51 African American/Black, 34 Hispanic/Latino, 1 Native American, 4 White/Anglo, 3 other<br><br>Education: 21 <11th grade, 45 high school/general education development, 25 2-years college, 9 4+ years of college	Quantitative	N/A	• **Aim:** to assess self-efficacy of people living with HIV regarding use of a hypothetical personal health record<br>• Participants received an explanation of a personal health record and completed questionnaires measuring their self-efficacy in relation to digital and paper-based personal health records, condom use and chronic disease management	N/A as no specific digital platform used	• Self-efficacy for creation and use of a paper-based personal health record<br>• Self-efficacy for creation and use of a digital personal health record<br>• Self-efficacy regarding medication adherence (HIV-Treatment Adherence Self-Efficacy Scale)<br>• Self-efficacy for chronic disease management scale<br>Self-efficacy for condom usage scale

Ramanathan et al. (2013)	Los Angeles, USA	N = 29 people living with HIV<br><br>Age: range= 30-60 years<br><br>Gender: 75% male<br><br>-Ethnicity: reported approximately one third of participants in each ethnic group of African American, Latino and White<br><br>Orientation: 55% men who have sex with men, 10% transgender	Qualitative, focus group discussions	N/A	• **Aim:** to assess feature preferences for a mobile app for HIV self-management<br>• **Topics addressed:** privacy, goal-setting, data capturing methods, feedback regarding app-user behaviours and the role of reminders	A hypothetical mobile app was discussed.	• Verbal feedback regarding key issues pertaining to development of a mobile app for HIV self-management

Swendeman et al. (2016)	Los Angeles, USA	N = 12 HCPs from a Los Angeles County HIV clinic<br>• 3 doctors<br>• 1 nurse practitioner<br>• 2 nurse case managers<br>• 2 psychosocial case managers<br>• 1 psychosocial case manager<br>• 1 psychotherapist<br><br>Age: unreported<br><br>Gender: unreported<br><br>Ethnicity: unreported<br><br>Orientation: unreported<br><br>Education: unreported	Qualitative, individual 45-60 minute semi-structured interviews	N/A	• **Aim:** to assess HCP attitudes regarding a hypothetical mobile app (for people living with HIV) and online dashboards (for HCPs) for HIV management<br>• **Topics addressed:** usability, acceptability and barriers to digital platform use	A hypothetical mobile app and online dashboard were discussed.	• Verbal feedback from participants regarding the use of hypothetical web-based dashboards for HCPs and hypothetical mobile apps for people living with HIV, both to facilitate self-management in patients

**Studies concerning a novel digital platform for PGData collection**							
Schnall et al. (2011)	New York, USA	N = 37 Case Managers working with people living with HIV<br><br>Age: unreported<br><br>Gender: unreported<br><br>Ethnicity: unreported<br><br>Orientation: unreported	Qualitative, one-hour focus group discussions	March-December 2008	• **Aim:** to determine factors influencing usage and acceptability of online “SelectHealth Continuity of Care Document” for people living with HIV, before the platform’s release<br>• **Topics addressed:** facilitators and barriers to use of the continuity of care record	The “SelectHealth Continuity of Care Document” includes key health information including latest medical test results, pharmaceutical record, visits to healthcare facilities and patient contact information	• Verbal feedback regarding perceived facilitators and barriers to use of the “SelectHealth Continuity of Care Document”
Schnall et al. (2016)	New York, USA	N = 15<br>• 10 people living with HIV<br>• 5 informaticians<br><br>Age: unreported<br><br>Gender (people living with HIV): 6 male<br><br>Ethnicity: unreported<br><br>Orientation: unreported	Mixed: quantitative, qualitative	[illegible]	• **Aim:** to test a prototype mobile app designed to facilitate HIV management in people living with HIV Participants received a description of a mobile app prototype for HIV management<br>• Informaticians tested the model for 45-90 minutes and completed a Heuristic Evaluation Checklist<br>• people living with HIV evaluated prototype app screenings and completed the Post Study System Usability Questionnaire	No description of mobile app prototype provided	• For informaticians: score on Heuristic Evaluation Checklist<br>• For people living with HIV: score on Post Study System Usability Questionnaire as a measurement of the app’s usability

Stonbraker et al. (2018)	New York, USA	N= 25<br>• 20 people living with HIV who possessed at least one HIV-associated non-AIDS condition<br>• 5 informaticians<br><br>Age: M= 54.4 years<br><br>Gender: 40% male<br><br>Ethnicity: only reported 85% African American<br><br>Orientation: unreported<br><br>Education: 50% <high school, 35% high school, 15% more than high school	Mixed: quantitative, qualitative	N/A	• **Aim:** to determine usability of novel mobile app *VIP-HANA* (Video Information Provider for HIV-associated non-AIDS conditions) designed to aid people living with HIV with management of HIV/non-HIV-related symptoms, and to determine where improvements to the app were needed<br>• After receiving descriptions of the app participants were assigned tasks on a beta version of the app and had to describe aloud their thoughts and actions whilst their activities were tracked<br>• people living with HIV completed one questionnaire to assess their health literacy level, and two questionnaires relating to the app's usability	• Within the app individuals must indicate which HIV-associated non-AIDS conditions they possess out of nine options<br>• Individuals can then categorise associated symptoms of the condition(s) out of 28 options, according to their severity<br>Upon severity categorisation, the app-user receives tailored strategies to facilitate self-management behaviours	• For informaticians: assigned mobile app task activity and score on Heuristic Evaluation Checklist<br>• For people living with HIV: assigned mobile app task activity, score on the Newest Vital Sign assessment (to determine health literacy level) and scores on two questionnaires relating to app usability (Health Information Technology Usability Evaluation Scale and Post Study System Usability Questionnaire)

**Studies concerning an existing, established digital platform for PGData collection**							
**Authors (date)**	**Location**	**Participants**	**Study design**	**Length of study**	• **Study aims and summary**	• **Digital platform summary**	• **Measures**
DeSilva et al. (2013)	Nanning, China	N= 10 people living with HIV who were injection drug users<br><br>Age: M = 32.7 years<br><br>Gender: 7 male<br><br>Orientation: unreported<br><br>Ethnicity: unreported<br><br>Education: 9 participants <middle school	Qualitative, individual interviews before and after using the device	One month	• **Aim:** to determine the feasibility and acceptability of using a *Wisepill* device in injecting drug users who were HIV positive<br>• Participants used the *Wisepill* device to monitor their antiretroviral medication adherence for one month<br>• **Topics addressed:** acceptability, feasibility and usability of the device	• The *Wisepill* container stores medication<br>• Upon opening, a wireless signal is immediately transmitted to a server, the data of which can be obtained by HCPs<br>• Opening the *Wisepill* container provides a proxy measurement of medication adherence	• Verbal feedback from participants regarding feasibility, acceptability and usability of the *Wisepill* device to track medication adherence<br>• Self-reported adherence to antiretroviral medication after one month of using the *Wisepill* device

Luque et al. (2013)	Rochester, New York, USA	N = 29 people living with HIV<br><br>Age: M= 48<br><br>Gender: 55% male, 41% female, 3% transgender<br><br>Ethnicity: 41% African American, 21% Latino, 28% white, 10% other<br><br>Orientation: unreported<br><br>Education level: 31% <high school, 28 % high school or equivalent, 41% >high school<br><br>Annual household income: 83% <$20,000, 17% >$20,000<br><br>Use of electronic technology: 18% never used a computer, 21% never used the internet, 36% never used email	Quantitative	6 weeks	• **Aim:** to determine acceptability and usability of a personal health record on an iPod touch device, and to observe the effect of using a digital personal health record upon HIV treatment self-efficacy<br>• Participants underwent six 90-minute training sessions and were given 'homework' tasks to rehearse between training sessions	• The *MyMedical* app, (downloaded from iTunes Store) was trialled as a personal health record on an iPod touch device<br><br>• No more information regarding the app was provided within the study	• HIV-Treatment Adherence Self-Efficacy Scale (baseline and one month post-training)<br>• Post-training questionnaire score for iPod device usability<br>• Post-training questionnaire score for personal health record usability<br>• Attitudes Towards Computers Scale

Odlum et al. (2014)	New York, USA	N= 57 people living with HIV<br>• Quantitative: 41 people living with HIV<br>-M age= 47 years, range 24-63 years<br><br>-Gender: 27 male<br><br>-Ethnicity: 27 Black/African American, 11 other/mixed race<br><br>-Orientation: unreported<br><br>-Education: 25 <high school diploma, 4 associate degrees, 2 Bachelor's degrees<br><br>• Qualitative: 15 people living with HIV,<br>8 users of the *MyHealthProfile* app<br><br>-Gender: 2 male<br><br>-Ethnicity: 7 Black African American, 1 Latino<br><br>-Orientation: unreported	Mixed: quantitative (System Usefulness Survey), qualitative (focus groups of 1 hour)	N/A	• **Aim:** to determine usability of the *MyHealthProfile* app and to determine where improvements could be made to inform a new version of the app named *MyHealthProfile-plus*<br>• Participants completed 2 surveys relating to the usefulness and content of the pre-established *My Health Profile* app<br>• Participants completed 60 minute focus groups regarding the development of new *My Health Profile-plus* app	• The *MyHealthProfile* app acts as a continuity of care document for people living with HIV to track their personal health record via an internet connection	• System Usefulness Survey<br>• Ease of Use Scale<br>• App usefulness score<br>• App components score<br>• Verbal feedback regarding perceived app needs for novel app *My Health Profile-plus*

		7 non-users of the *MyHealthProfile* app<br>-Gender: 5 male<br>-Ethnicity: 6 Black African American, 1 Caucasian<br><br>-Orientation: unreported					
