# Supplementary material for: Patient-generated data in the management of HIV: a scoping review
Source: BMJ Open. 2021 May 19;11(5):e046393. doi: 10.1136/bmjopen-2020-046393 (PMC8137219; doi:10.1136/bmjopen-2020-046393)
Supplement: Supplementary data [file bmjopen-2020-046393supp004.pdf]

Appendix D- Results from the Mixed Methods Appraisal Tool assessment

Bussone (2018)

		Responses		
**Study design**	**Methodological quality criteria**	**Yes**	**No**	**Can't tell**
**Screening questions (all studies)**	S1. Are there clear research questions?	X		
	S2. Do the collected data allow to address the research questions?	X		
**Qualitative**	1.1. Is the qualitative approach appropriate to answer the research question?	X		
	1.2. Are the qualitative data collection methods adequate to address the research question?	X		
	1.3. Are the findings adequately derived from the data?	X		
	1.4. Is the interpretation of results sufficiently substantiated by data?	X		
	1.5. Is there coherence between qualitative data sources, collection, analysis and interpretation?	X		

BMJ Publishing Group Limited (BMJ) disclaims all liability and responsibility arising from any reliance placed on this supplemental material which has been supplied by the author(s)

DeSilva et al. (2013)

		Responses		
**Study design**	**Methodological quality criteria**	**Yes**	**No**	**Can't tell**
**Screening questions (all studies)**	S1. Are there clear research questions?	X		
	S2. Do the collected data allow to address the research questions?	X		
**Qualitative**	1.1. Is the qualitative approach appropriate to answer the research question?	X		
	1.2. Are the qualitative data collection methods adequate to address the research question?	X		
	1.3. Are the findings adequately derived from the data?			X
	1.4. Is the interpretation of results sufficiently substantiated by data?			X
	1.5. Is there coherence between qualitative data sources, collection, analysis and interpretation?			X

Luque et al. (2013)

		Responses		
**Study design**	**Methodological quality criteria**	**Yes**	**No**	**Can't tell**
**Screening questions (all studies)**	S1. Are there clear research questions?	X		
	S2. Do the collected data allow to address the research questions?	X		
**Quantitative descriptive**	4.1. Is the sampling strategy relevant to address the research question?	X		
	4.2. Is the sample representative of the target population?		X	
	4.3. Are the measurements appropriate?	X		
	4.4. Is the risk of nonresponse bias low?	X		
	4.5. Is the statistical analysis appropriate to answer the research question?	X		

Marent, Henwood & Darking (2018)

		Responses		
**Study design**	**Methodological quality criteria**	**Yes**	**No**	**Can't tell**
**Screening questions (all studies)**	S1. Are there clear research questions?	X		
	S2. Do the collected data allow to address the research questions?	X		
**Qualitative**	1.1. Is the qualitative approach appropriate to answer the research question?	X		
	1.2. Are the qualitative data collection methods adequate to address the research question?	X		
	1.3. Are the findings adequately derived from the data?	X		
	1.4. Is the interpretation of results sufficiently substantiated by data?	X		
	1.5. Is there coherence between qualitative data sources, collection, analysis and interpretation?	X		

Nokes et al. (2013)

		Responses		
**Study design**	**Methodological quality criteria**	**Yes**	**No**	**Can't tell**
**Screening questions (all studies)**	S1. Are there clear research questions?	X		
	S2. Do the collected data allow to address the research questions?	X		
**Quantitative descriptive**	4.1. Is the sampling strategy relevant to address the research question?	X		
	4.2. Is the sample representative of the target population?	X		
	4.3. Are the measurements appropriate?	X		
	4.4. Is the risk of nonresponse bias low?	X		
	4.5. Is the statistical analysis appropriate to answer the research question?	X		

Odlum et al. (2014)

		Responses		
**Study design**	**Methodological quality criteria**	**Yes**	**No**	**Can't tell**
**Screening questions (all studies)**	S1. Are there clear research questions?	X		
	S2. Do the collected data allow to address the research questions?	X		
**Qualitative**	1.1. Is the qualitative approach appropriate to answer the research question?	X		
	1.2. Are the qualitative data collection methods adequate to address the research question?	X		
	1.3. Are the findings adequately derived from the data?	X		
	1.4. Is the interpretation of results sufficiently substantiated by data?	X		
	1.5. Is there coherence between qualitative data sources, collection, analysis and interpretation?	X		
**Quantitative descriptive**	4.1. Is the sampling strategy relevant to address the research question?			X

	4.2. Is the sample representative of the target population?		X	
	4.3. Are the measurements appropriate?			X
	4.4. Is the risk of nonresponse bias low?			X
	4.5. Is the statistical analysis appropriate to answer the research question?			X
**Mixed methods**	5.1. Is there an adequate rationale for using a mixed methods design to address the research question?		X	
	5.2. Are the different components of the study effectively integrated to answer the research question?		X	
	5.3. Are the outputs of the integration of qualitative and quantitative components adequately interpreted?		X	
	5.4. Are divergences and inconsistencies between quantitative and qualitative results adequately addressed?		X	
	5.5. Do the different components of the study adhere to the quality criteria of each tradition of the methods involved?			X

Ramanathan et al. (2013)

		Responses		
**Study design**	**Methodological quality criteria**	**Yes**	**No**	**Can't tell**
**Screening questions (all studies)**	S1. Are there clear research questions?	X		
	S2. Do the collected data allow to address the research questions?	X		
**Qualitative**	1.1. Is the qualitative approach appropriate to answer the research question?	X		
	1.2. Are the qualitative data collection methods adequate to address the research question?	X		
	1.3. Are the findings adequately derived from the data?	X		
	1.4. Is the interpretation of results sufficiently substantiated by data?	X		
	1.5. Is there coherence between qualitative data sources, collection, analysis and interpretation?	X		

Swendeman et al. (2016)

Study design	Methodological quality criteria	Responses		
		**Yes**	**No**	**Can't tell**
**Screening questions (all studies)**	S1. Are there clear research questions?	X		
	S2. Do the collected data allow to address the research questions?	X		
**Qualitative**	1.1. Is the qualitative approach appropriate to answer the research question?	X		
	1.2. Are the qualitative data collection methods adequate to address the research question?	X		
	1.3. Are the findings adequately derived from the data?	X		
	1.4. Is the interpretation of results sufficiently substantiated by data?	X		
	1.5. Is there coherence between qualitative data sources, collection, analysis and interpretation?	X		

Schnall et al. (2011)

		Responses		
**Study design**	**Methodological quality criteria**	**Yes**	**No**	**Can't tell**
**Screening questions (all studies)**	S1. Are there clear research questions?	X		
	S2. Do the collected data allow to address the research questions?	X		
**Qualitative**	1.1. Is the qualitative approach appropriate to answer the research question?	X		
	1.2. Are the qualitative data collection methods adequate to address the research question?	X		
	1.3. Are the findings adequately derived from the data?	X		
	1.4. Is the interpretation of results sufficiently substantiated by data?	X		
	1.5. Is there coherence between qualitative data sources, collection, analysis and interpretation?	X		

Schnall et al. (2016)

		Responses		
**Study design**	**Methodological quality criteria**	**Yes**	**No**	**Can't tell**
**Screening questions (all studies)**	S1. Are there clear research questions?	X		
	S2. Do the collected data allow to address the research questions?		X	
**Qualitative**	1.1. Is the qualitative approach appropriate to answer the research question?			X
	1.2. Are the qualitative data collection methods adequate to address the research question?			X
	1.3. Are the findings adequately derived from the data?			X
	1.4. Is the interpretation of results sufficiently substantiated by data?			X
	1.5. Is there coherence between qualitative data sources, collection, analysis and interpretation?			X
**Quantitative descriptive**	4.1. Is the sampling strategy relevant to address the research question?		X	

	4.2. Is the sample representative of the target population?		X	
	4.3. Are the measurements appropriate?			X
	4.4. Is the risk of nonresponse bias low?			X
	4.5. Is the statistical analysis appropriate to answer the research question?			N/A
**Mixed methods**	5.1. Is there an adequate rationale for using a mixed methods design to address the research question?		X	
	5.2. Are the different components of the study effectively integrated to answer the research question?		X	
	5.3. Are the outputs of the integration of qualitative and quantitative components adequately interpreted?		X	
	5.4. Are divergences and inconsistencies between quantitative and qualitative results adequately addressed?		X	
	5.5. Do the different components of the study adhere to the quality criteria of each tradition of the methods involved?		X	

Stonbraker et al. (2018)

		Responses		
**Study design**	**Methodological quality criteria**	**Yes**	**No**	**Can't tell**
**Screening questions (all studies)**	S1. Are there clear research questions?	X		
	S2. Do the collected data allow to address the research questions?	X		
**Qualitative**	1.1. Is the qualitative approach appropriate to answer the research question?			X
	1.2. Are the qualitative data collection methods adequate to address the research question?			X
	1.3. Are the findings adequately derived from the data?	X		
	1.4. Is the interpretation of results sufficiently substantiated by data?	X		
	1.5. Is there coherence between qualitative data sources, collection, analysis and interpretation?	X		
**Quantitative descriptive**	4.1. Is the sampling strategy relevant to address the research question?			X

	4.2. Is the sample representative of the target population?		X	
	4.3. Are the measurements appropriate?			X
	4.4. Is the risk of nonresponse bias low?			X
	4.5. Is the statistical analysis appropriate to answer the research question?			X
**Mixed methods**	5.1. Is there an adequate rationale for using a mixed methods design to address the research question?		X	
	5.2. Are the different components of the study effectively integrated to answer the research question?		X	
	5.3. Are the outputs of the integration of qualitative and quantitative components adequately interpreted?		X	
	5.4. Are divergences and inconsistencies between quantitative and qualitative results adequately addressed?		X	
	5.5. Do the different components of the study adhere to the quality criteria of each tradition of the methods involved?		X	
