# Supplementary material for: Patient-generated data in the management of HIV: a scoping review
Source: BMJ Open. 2021 May 19;11(5):e046393. doi: 10.1136/bmjopen-2020-046393 (PMC8137219; doi:10.1136/bmjopen-2020-046393)
Supplement: Author's manuscript [file bmjopen-2020-046393.draft_revisions.pdf]

BMJ Open

BMJ Open is committed to open peer review. As part of this commitment we make the peer review history of every article we publish publicly available.

When an article is published we post the peer reviewers' comments and the authors' responses online. We also post the versions of the paper that were used during peer review. These are the versions that the peer review comments apply to.

The versions of the paper that follow are the versions that were submitted during the peer review process. They are not the versions of record or the final published versions. They should not be cited or distributed as the published version of this manuscript.

BMJ Open is an open access journal and the full, final, typeset and author-corrected version of record of the manuscript is available on our site with no access controls, subscription charges or pay-per-view fees (http://bmjopen.bmj.com).

If you have any questions on BMJ Open's open peer review process please email info.bmjopen@bmj.com

BMJ Open

# Patient-generated data in the management of HIV: a scoping review

| | |
|---|---|
| Journal: | *BMJ Open* |
| Manuscript ID | bmjopen-2020-046393 |
| Article Type: | Original research |
| Date Submitted by the Author: | 02-Nov-2020 |
| Complete List of Authors: | Hewitt, Clara; University College London, Institute for Global Health<br>Lloyd, Karen; University College London, Centre for Population Research in Sexual Health and HIV, Institute for Global Health<br>Tariq, Shema; University College London, Institute for Global Health; University College London<br>Durrant, Abigail; Newcastle University, Open Lab, School of Computing<br>Claisse, Caroline; Newcastle University, Open Lab, School of Computing<br>Kasadha, Bakita; Terrence Higgins Trust<br>Gibbs, Jo; University College London, Research Department of Infection and Population Health; |
| Keywords: | HIV & AIDS < INFECTIOUS DISEASES, Telemedicine < BIOTECHNOLOGY & BIOINFORMATICS, GENITOURINARY MEDICINE |
| | |

SCHOLARONE™
Manuscripts

*I, the Submitting Author has the right to grant and does grant on behalf of all authors of the Work (as defined in the below author licence), an exclusive licence and/or a non-exclusive licence for contributions from authors who are: i) UK Crown employees; ii) where BMJ has agreed a CC-BY licence shall apply, and/or iii) in accordance with the terms applicable for US Federal Government officers or employees acting as part of their official duties; on a worldwide, perpetual, irrevocable, royalty-free basis to BMJ Publishing Group Ltd ("BMJ") its licensees and where the relevant Journal is co-owned by BMJ to the co-owners of the Journal, to publish the Work in this journal and any other BMJ products and to exploit all rights, as set out in our licence.*

*The Submitting Author accepts and understands that any supply made under these terms is made by BMJ to the Submitting Author unless you are acting as an employee on behalf of your employer or a postgraduate student of an affiliated institution which is paying any applicable article publishing charge ("APC") for Open Access articles. Where the Submitting Author wishes to make the Work available on an Open Access basis (and intends to pay the relevant APC), the terms of reuse of such Open Access shall be governed by a Creative Commons licence – details of these licences and which Creative Commons licence will apply to this Work are set out in our licence referred to above.*

*Other than as permitted in any relevant BMJ Author's Self Archiving Policies, I confirm this Work has not been accepted for publication elsewhere, is not being considered for publication elsewhere and does not duplicate material already published. I confirm all authors consent to publication of this Work and authorise the granting of this licence.*

For peer review only

**BMJ Open**

# Patient-generated data in the management of HIV: a scoping review

**Authors and affiliations**

Clara Hewitt[1]
Karen C Lloyd[1]
Shema Tariq[1]
Abigail Durrant[2]
Caroline Claisse[2]
Bakita Kasadha[3]
Jo Gibbs[1]

1 Institute for Global Health, University College London, UK

2 Open Lab, School of Computing, Newcastle University, Newcastle upon Tyne, UK

3 Terrence Higgins Trust, London, UK

**Correspondence to:**
Dr. Karen C. Lloyd, Mortimer Market Centre, Off Capper St, Bloomsbury, London WC1E 6JB; Karen.lloyd@ucl.ac.uk

**Word count** (excluding title page, abstract, tables, contributions and references): **3,118**

# ABSTRACT

**Objectives**

Patient-generated data (PGData) is an emergent research area and may improve HIV care. The objectives of this scoping review were to synthesise, evaluate and make recommendations based upon the available literature regarding PGData use in HIV care.

**Design:** Scoping review

**Search Strategy**

We systematically searched the Embase, Medline, CINAHL Plus, Web of Science, Scopus, PsycINFO and Emcare databases for studies involving PGData use within HIV care for people living with HIV and/or healthcare professionals (HCPs). We used Thematic Analysis to evaluate content and assessed empirical rigour using the Mixed Methods Appraisal Tool.

**Results**

Studies were observational, predominantly concerned hypothetical or novel digital platforms, mainly conducted in high-income settings (10/11, with 8 conducted in North America), and had small sample sizes (range=10-160). There were multiple definitions of PGData. In the majority of studies (n= 9) participants were people living with HIV, with a few studies including HCPs, informatics specialists, or mixed participant groups. Participants living with HIV were aged 23-78, mostly male, of diverse ethnicities, and had low educational, health literacy and income levels.

We identified four key themes: 1. Perceptions of PGData and associated digital platforms; 2. Opportunities; 3. Anticipated barriers; and 4. Potential impact upon patient-HCP relationships.

**Conclusions**

Use of PGData within HIV care warrants further study, especially with regard to digital inequalities, data privacy and security. There is a need for longitudinal data on facilitators and barriers to PGData use within HIV in a variety of settings with a broad range of users, as well as investigation of its impact on clinical outcomes. This will allow greater understanding of the role of PGData use in improving the health and wellbeing of people living with HIV, which is increasingly pertinent as digital healthcare becomes more widespread as a result of COVID-19.


Keywords: HIV; digital health; self-management; patient generated health data.

**Strengths and limitations of this study:**

- This scoping review provides an overview and synthesis of the emergent literature of PGData use within HIV care which can provide a foundation of understanding to inform future research.
- The Mixed Methods Appraisal Tool allowed assessment of mixed study designs.
- Literature searching yielded a limited number of articles, with most articles obtained via manual referencing.
- Only English studies were reviewed, which may bias and thus narrow the scope of the findings to English speaking settings.

## Background

Human Immunodeficiency Virus (HIV) remains a major global health challenge. Antiretroviral therapy (ART) has extended the life expectancy of people living with HIV, transforming HIV into a chronic condition. [1] Effective HIV care requires both long-term clinical follow-up as well as high levels of adherence to ART. This facilitates viral suppression, reducing HIV-related morbidity and mortality, as well as preventing onward HIV transmission. [2] However, HIV care is often complex; people living with HIV have a greater risk of co-morbid conditions such as poor mental health and cardiovascular disease compared to their HIV-negative counterparts. [3] [4] These co-morbidities (often a result of the virus, side effects of ART, and/or complex socioeconomic factors) may require considerable input from a wide range of healthcare professionals (HCPs) which has a consequential impact on healthcare resources, even in high income settings. [1] [5] With health systems already financially and time-poor, it is important to identify innovative approaches to HIV care that can support quality of life, while addressing the challenges posed by increasing co-morbidities and an ageing population. Digital technologies present opportunities through which to improve HIV care, and in turn, the health and quality of life of people living with HIV. [6]

The role of self-management in the care of long-term conditions such as HIV is becoming increasingly important. [6] The ubiquity of digital devices and applications used for health purposes can support self-management and has created fertile grounds for patient generated data (PGData) collection. [7] [8] Although no universal PGData definition exists, multiple authors across the literature have adopted the broad definition [9] that PGData constitute any health-related data which are created or collected by patients or designated proxies to address a health concern. [7] [10] [11] [12] [13] [14] As such, PGData include medication dosages, physical activity, dietary intake, sleep and mood patterns, and can be collected in

different modalities including text, pictures, voice or video recordings, and numerical information e.g. questionnaire scores or physiological measurements. [11] [15] PGData allow more frequent, remote and longitudinal tracking of multiple types of health information, and can be rapidly shared between patients and HCPs, or between patients themselves, usually using wireless connections. [13] [16] Additionally, PGData can serve as an adjunct to health information communicated during clinical consultations; it can demonstrate baseline health measures bespoke to each individual, which may ultimately facilitate more patient-centred, personalised care. [8] [14] [17] Consequently, PGData allow greater real-time insight into health fluctuations or anomalies relative to a baseline, permitting holistic appreciation of patient health for both patients and their HCPs, and potentially enabling more appropriate and timely interventions. [5] [9] [10] [11] [15] [16] [18]

Whilst the use of PGData for the management of other long-term conditions such as cancer [14] and diabetes [18] has been investigated, to our knowledge, no review of PGData use within HIV care has been conducted to date. This scoping review is timely, given that COVID-19 has led to rapidly increased adoption of remote healthcare and telemedicine practices within clinical care, which will likely remain in place beyond the current pandemic.

**Objectives:**

This scoping review aimed to 1. Synthesise and evaluate the existing emergent literature on PGData use within HIV care, 2. Identify the opportunities and challenges it presents and 3. Consider the impact of PGData use upon people living with HIV and their HCPs.



For peer review only - http://bmjopen.bmj.com/site/about/guidelines.xhtml

## METHODS

We conducted a scoping review of literature on PGData use within HIV care. We adopted this approach to enable a broad focus and preliminary synthesis of available evidence [19] in this new, emerging research area. We have reported the review in accordance with the Preferred Reporting Items for Systematic Reviews and Meta-analysis extension for Scoping Reviews (PRISMA-ScR) guidelines [20] (see Appendix A). A review protocol has not been registered.

### Search strategy

During July 2019, we searched Medline, Embase, Emcare, CINAHL, PsycINFO, Scopus and Web of Science databases for articles. Additional grey literature databases were searched using the same strategy: National Institute of Clinical Excellence: Health and Social Care, TRIP medical database, ProQuest Dissertations and Theses, OpenAIRE, Bielefeld Academic Search Engine (BASE), OpenDOAR, Open Access Dissertations and Theses, DART-Europe, Open Grey and Semantic Scholar. Searches were restricted to English language and the terms used were adapted to meet the requirements of each database. A detailed search terms can be found in Appendix B. Article reference lists were also manually searched to obtain additional literature which the formal search strategy may have omitted.



For peer review only - http://bmjopen.bmj.com/site/about/guidelines.xhtml

**Eligibility Criteria**

Study inclusion was contingent on the terms outlined in Table 1 and temporal limits were not imposed to keep the scope of literature generated as broad as possible given the infancy of the literature base.



For peer review only - http://bmjopen.bmj.com/site/about/guidelines.xhtml

| Inclusion Criteria |
| --- |
| • Studies which considered the perspectives of HCPs or people living with HIV regarding PGData use within HIV management<br>• Studies where PGData use met the definition adopted [10]<br>• Where studies involved patients, these needed to be people living with HIV<br>• PGData collection by people living with HIV had to be conducted via a digital platform<br>• PGData collected in relation to living with HIV<br>• Any PGData created had to be collected independently by people living with HIV, and not in response to a reminder or prompt |
| **Exclusion Criteria** |
| • Studies involving PGData which were not generated independently by people living with HIV, or where PGData were created in response to the receipt of a reminder or prompt<br>• Studies concerning PGData use in the diagnosis, prevention or risk reduction of HIV<br>• Studies solely involving a two-way communication system, including studies involving digital messaging or forum platforms<br>• Conference proceedings |

***Table 1.*** **Summary of inclusion and exclusion criteria.**



For peer review only - http://bmjopen.bmj.com/site/about/guidelines.xhtml

**Selection of sources of evidence**

The retrieved articles were exported into Mendeley Reference Manager and duplicates were removed. Titles and abstracts were screened, and full texts were assessed using the eligibility criteria. A data extraction table (see Appendix C) was used to summarise data from articles meeting the eligibility criteria for analysis.

**Empirical appraisal and thematic analysis of included articles**

Due to heterogeneity of study designs, empirical quality was assessed using the Mixed Methods Appraisal Tool (MMAT, [21] see Appendix D). Thematic analysis [22] was conducted based upon the qualitative findings from the identified articles.

For the thematic analysis, primarily individual article-specific mind maps were created to highlight key concepts. [23] Subsequently, a comprehensive mind map was created to synthesise key topics across the reviewed articles, which were finally organised into four overarching themes and three sub-themes.

**Patient and public involvement**

This manuscript reports on a review of the literature and no patients or members of the public were explicitly involved in the scoping review process, however, one co-author is a peer researcher employed by a third sector organisation.

## RESULTS

**Search results**

Literature searches were conducted in June 2019. A total of 2,353 articles were identified through academic databases and grey literature keyword searching (Figure 1). Seventy-three full-text articles were assessed, of which five fulfilled the eligibility criteria for inclusion. A further six eligible studies were identified from a manual search of reference lists.

**[Figure 1. PRISMA Flow Diagram]**

**Participants and demographics**

Sample sizes of included studies ranged from 10-160 participants. Participants were predominantly people living with HIV, however, some studies involved HCPs, allied HCPs, informatics specialists, or mixed participant groups (see Appendix C). Apart from one study,[24] where nurses and doctors were participants, role specialisms were not identified. Where reported, studies primarily included men who have sex with men (MSM) and heterosexual men and women. The age range of people living with HIV across the studies was 23-78 years. Participants were mostly male and of Black (including Black African and African American) ethnicity. Only two studies involved transgender people living with HIV, despite this being a HIV key population. Where reported, people living with HIV were most often from low-income, low-educational backgrounds.

**Study characteristics**

The empirical quality of the 11 articles reviewed was varied (see MMAT assessment, Appendix D). Articles were grouped into three categories according to the type of digital platform used; hypothetical, prototype or pre-established (see Table 2). Studies were predominantly conducted in the USA (eight studies [24] [25] [26] [27] [28] [29] [30] [31]), with others based in China [32], England [33] and other parts of Europe [34]. All PGData platforms were mobile or web-based except for one study involving a medication storage device. [32] Study designs included user testing, [32] [28] [29] [25] focus groups [31] [26] [30] and semi-structured interview sessions. [24] [33] [34] One study also included patient-HCP co-design workshops. [34]

User testing studies varied in structure; two studies trialled beta mobile application (app) prototypes for PGData collection wherein people living with HIV and informaticians completed tasks whilst simultaneously voicing feedback, [28] [29] whilst others examined the impact of medication storage device use [32] and a six-week personal health record app training programme for people living with HIV. [25] User testing studies focused on the



For peer review only - http://bmjopen.bmj.com/site/about/guidelines.xhtml

perceived usability and feasibility of PGData collection and associated digital platforms; some studies employed quantitative assessments to measure these [25] [28] [29] (see Appendix C). No studies conducted user testing with HCPs. Studies conducting interviews and focus group discussions tended to use open-ended questions or follow discussion guides, and centred around issues of PGData platform acceptability, feasibility, and anticipated barriers and facilitators to PGData adoption.

| **Studies concerning hypothetical digital platforms** | | | |
|---|---|---|---|
| ***Author(s)*** | ***Participants, design, location, study length*** | ***Digital platform*** | ***Overview*** |
| **Bussone (2018)** | -N = 16 people living with HIV<br>- Qualitative, individual semi-structured interviews of approximately 30 minutes<br>-England (UK)<br>-Study length unspecified | No specific digital platform used | Aimed to understand how people living with HIV monitor their personal health data. Addressed topics including participants' current self-management behaviours and issues surrounding current, previous or desired means of tracking personal health information. |
| **Marent, Henwood & Darking (2018)** | -N = 160 participants<br>• 97 people living with HIV<br>• 63 HCPs working in HIV care<br>-40 doctors<br>-10 nurses<br>-4 psychologists<br>-4 pharmacists<br>-2 social workers<br>-2 nutritionists<br>• 1 sexologist<br>- Qualitative, 14 recorded workshops and 22 semi-structured interviews<br>-Brighton (UK), Lisbon (Portugal), Barcelona (Spain), Antwerp (Belgium) and Zagreb (Croatia)<br>-January-June 2016 | No specific digital platform used | Aimed to determine perceptions of, and advantages and concerns surrounding, the development of a mobile app for HIV management. Addressed topics including perceived challenges and barriers to use of a mobile app for HIV management, current mobile app usage and perceived useful features of a mobile app for HIV management. |

| | | | |
|---|---|---|---|
| **Nokes et al. (2013)** | -N = 100 people living with HIV or AIDS<br>-Quantitative<br>-New York, USA<br>-Study length unspecified | No specific digital platform used | Aimed to assess self-efficacy of people living with HIV regarding use of a hypothetical personal health record. Participants received an explanation of a personal health record and completed questionnaires measuring their self-efficacy in relation to digital and paper-based personal health records, condom use and chronic disease management. |
| **Ramanathan et al. (2013)** | -N= 29 people living with HIV<br>-Qualitative: focus group discussions<br>-Los Angeles, USA<br>-Study length unspecified | No specific digital platform used | Aimed to assess feature preferences for a mobile app for HIV self-management. Addressed topics including privacy, goal-setting, data capturing methods, feedback regarding app-user behaviours and the role of reminders. |
| **Swendeman et al. (2016)** | - N = 12 HCPs working in HIV care<br>• 3 doctors<br>• 5 nurses<br>• 2 psychosocial case workers<br>• 1 outreach worker<br>• 1 psychotherapist<br>- Qualitative, individual 45-60 minute semi-structured interviews<br>-Los Angeles, USA<br>-Study length unspecified | No specific digital platform used | Aimed to assess HCP attitudes regarding a hypothetical mobile app (for people living with HIV) and online dashboards (for HCPs) for HIV management. Addressed topics including usability, acceptability and barriers to digital platform use. |
| **Studies concerning prototype digital platforms** | | | |
| ***Author(s)*** | ***Participants, design, location, study length*** | ***Digital platform*** | ***Overview*** |
| **Schnall et al. (2011)** | - N = 37 Case Managers working with people living with HIV<br>- Qualitative, one-hour focus group discussions<br>-New York, USA<br>- March-December 2008 | SelectHealth Continuity of Care Document; includes key health information including latest medical test results, pharmaceutical record, visits to healthcare facilities and patient contact information | Aimed to determine factors influencing usage and acceptability of online “SelectHealth Continuity of Care Document” for people living with HIV, before the platform’s release. Addressed topics including facilitators and barriers to use of the continuity of care record. |


For peer review only - http://bmjopen.bmj.com/site/about/guidelines.xhtml

| | | | |
|---|---|---|---|
| **Schnall et al. (2016)** | -N = 15<br>• 10 people living with HVI<br>• 5 informaticians<br>- Mixed: quantitative, qualitative<br>-New York, USA<br>-Study length unspecified | No description of app prototype provided | Aimed to test a prototype mobile app designed to facilitate HIV management in people living with HIV. Participants received a description of a mobile app prototype for HIV management. Informaticians tested the model for 45-90 minutes and completed a Heuristic Evaluation Checklist<br>people living with HIV evaluated prototype app screenings and completed the Post Study System Usability Questionnaire. |
| **Stonbraker et al. (2018)** | -N= 25<br>• 20 people living with HIV who possessed at least one HIV-associated non-AIDS condition<br>• 5 informaticians<br>-Mixed: quantitative; qualitative<br>-New York, USA | -Novel mobile app "VIP-HANA" (Video Information Provider (VIP) for HIV-associated non-AIDS conditions) | Aimed to determine usability of novel mobile app "VIP-HANA" designed to aid people living with HIV with management of HIV/non-HIV-related symptoms, and to determine where improvements to the app were needed. After receiving descriptions of the app participants were assigned tasks on a beta version of the app and had to describe aloud their thoughts and actions whilst their activities were tracked. People living with HIV completed one questionnaire to assess their health literacy level, and two questionnaires relating to the app's usability. |
| **Studies concerning pre-established digital platforms** | | | |
| ***Author(s)*** | ***Participants, design,***<br>***location, study length*** | ***Digital platform*** | ***Overview*** |
| **DeSilva et al. (2012)** | -N= 10 people living with HIV who used injection drugs<br>-Individual qualitative interviews<br>-Nanning, China<br>-Study length unspecified | *Wisepill* medication storage container; once opened a wireless signal immediately transmits to a server, the data of which can be obtained by HCPs | Aimed to determine the feasibility and acceptability of using a "Wisepill" device in injecting drug users who were HIV positive. Participants used the "Wisepill" device to monitor their antiretroviral medication adherence for one month. Addressed topics including acceptability, feasibility and usability of the device. |
| **Luque et al. (2013)** | -N=29 people living with HIV<br>-Quantitative<br>-Rochester, New York, USA<br>-6 weeks | *MyMedical* app, (downloaded from iTunes Store). | Aimed to determine acceptability and usability of using the *MyMedical* app as a personal health record on an iPod touch device, and to observe the effect of using a digital personal health record upon HIV treatment self-efficacy. Participants underwent six 90-minute training sessions and were given 'homework' tasks to rehearse between training sessions. |



For peer review only - http://bmjopen.bmj.com/site/about/guidelines.xhtml

| | | | |
|---|---|---|---|
| **Odlum et al. (2014)** | -N= 57 people living with HIV<br>• Quantitative: 42 people living with HIV<br>• Qualitative: 15 people living with HIV; 8 users of the *MyHealthProfile* app, 7 non-users of the *MyHealthProfile* app<br>-Mixed: quantitative, qualitative<br>-New York, USA<br>-Study length unspecified | *MyHealthProfile* app; acts as a continuity of care people for living with HIV to track their personal health record via an internet connection | Aimed to determine usability of the *MyHealthProfile* app and to determine where improvements could be made to inform a new version of the app named *MyHealthProfile-plus*. Participants completed 2 surveys relating to the usefulness and content of the pre-established *My Health Profile* app. Participants completed 60 minute focus groups regarding the development of new *My Health Profile-plus* app |

*Table 2.* **Summary of included articles**

**Overview of Key Themes**

Our analysis highlighted four primary cross-cutting themes and three sub-themes, including: perceived acceptability, feasibility and usability of PGData; opportunities presented by PGData; anticipated barriers to PGData use; and how PGData may impact HIV care and HCP-patient relationships.

**Acceptability, feasibility and usability of digital platforms for PGData**

Participants' views regarding the acceptability, feasibility and usability of PGData differed. Some people living with HIV were receptive to digitally tracking their PGData, though this was contingent upon data security. [30] Widespread smartphone access, absence of PGData platform cost, clear design and easy navigability of digital PGData platforms increased the perceived feasibility of PGData use. [26] [33] [24] [32]

Perceptions regarding the perceived suitability of PGData use for people living with HIV varied; some HCPs suggested PGData use would be better suited to newly-diagnosed individuals, those with multi-morbidities, or higher education levels [24] [34] whilst others proposed newly-diagnosed people living with HIV may be unsuitable. [34] Some HCPs questioned whether individual differences including motivation may affect PGData collection



For peer review only - http://bmjopen.bmj.com/site/about/guidelines.xhtml

and engagement. [24] No data from people living with HIV regarding suitability were reported.

**Opportunities presented by PGData for HIV care**

Participants cited several perceived benefits of PGData use within HIV care, including reduced financial and time burdens, [31] convenience, and increased health proactivity. [26] [31] [33] Some people living with HIV perceived PGData collection could increase their medication adherence, perceived control, understanding and engagement surrounding their HIV care [33] due to their awareness of ongoing PGData monitoring. [24] [32] [30]

**Anticipated barriers and challenges associated with PGData**

Across the studies, several perceived challenges to PGData use emerged, including privacy and security, accessibility and reliability of PGData, and impact on HCPs.

Privacy and security

Participants expressed substantial privacy and security concerns regarding PGData collection and analysis, [31] [30] [25] [26] which some authors attributed to persisting HIV stigmatisation. [31] [32] [34] Participants voiced concerns that poor security and privacy may result in unwanted disclosures of sensitive data including HIV status, although how this might occur was not explicitly articulated. [36] [38] [32] [40] Several people living with HIV emphasised desires for security mechanisms including encryption, [33] [32] [30] [34] and some people living with HIV favoured paper-based PGData tracking methods due to digital platform apprehension. [33] [30]

Accessibility and reliability of digital infrastructure and collected PGData

There were prevalent concerns regarding the reliability of digital platforms and PGData itself. Some studies reported concerns that technological disruptions and financial instability may hinder digital platform accessibility and usage. [24] [32] Additionally, some



For peer review only - http://bmjopen.bmj.com/site/about/guidelines.xhtml

participants highlighted PGData reliability could be impaired by incorrect PGData recording. [31] Some people living with HIV warned of intentional retrospective editing to deliberately manipulate PGData. [30] [33]

Impact upon HCPs

HCPs were apprehensive that pre-existing time constraints and large workloads would hinder PGData-reviewing and that PGData involvement would only magnify these burdens. [24] [34] People living with HIV did not comment on the impact of PGData upon HCPs.

**Perceived impacts of PGData upon patient-HCP relationships**

Participants indicated engaging with PGData within clinical consultations could allow a more comprehensive picture of how a person may be managing their HIV to be communicated. [33] [40] [39] [36] Whilst people living with HIV desired PGData platforms allowing two-way communication with HCPs, HCPs feared increased workloads. [24]

Some people living with HIV perceived PGData use may provide opportunities to strengthen patient-HCP relationships. [34] However contrastingly, other participants expressed concern that PGData misinterpretation could impair pre-established face-to-face patient-HCP interactions. [30] Both people living with HIV and HCPs believed PGData use could complement, rather than supersede, traditional in-person HIV consultations, although details of such a hybrid model were unaddressed. [34]

## DISCUSSION

The current review provides a critical synthesis of the nascent literature base surrounding PGData use specifically for HIV care, and highlights several outstanding questions. Across the eleven articles, study designs and methodologies were varied and a spectrum of digital PGData platforms were considered. The views of people living with HIV and HCPs were broadly consistent, with exceptions regarding the impact of PGData upon patient-HCP relationships.



For peer review only - http://bmjopen.bmj.com/site/about/guidelines.xhtml

Individual and contextual differences

Our findings suggest that PGdata use may not be suitable for all people living with HIV; indeed, perceptions of PGData and its use within HIV care may differ greatly across patients and contexts. [35] [36] For example, whilst some patients felt reassured by monitoring their long-term health condition, for others, health monitoring could be tiring, intrusive, anxiety-provoking and excessively time-consuming. [32] [34] [37] [38] It is important that digital health interventions to support HIV care and self-management do not further reinforce stigma and use language that is accessible to all users.[39] As the majority of studies did not include participants from across key HIV populations, such as women, people who are transgender, or injecting drug users, knowledge of PGData use in these communities remains limited. [15] The current review's bias towards high-income settings further limits its generalisability given the global HIV burden lies predominantly in low and middle-income settings. [40] Furthermore, digital inequalities due to scarce digital access and poor digital literacy [41] [42] may impact PGData acceptability, feasibility and inequalities in PGData use, with the COVID-19 pandemic starkly highlighting these digital divides. [43] [44] Further research is needed to better understand these individual and contextual nuances in PGData use.

Impact upon patient-HCP relationships

The findings highlight dichotomies between people living with HIV and HCPs in this area; whilst people living with HIV appeared to welcome increased HCP contact and HCP-analysed PGData, there were concerns of placing greater burdens and expectations upon HCPs. [11] [14] [16] [45] [46] A PGData guidance framework may be necessary to counteract competing priorities between HCPs and patients regarding such communication preferences.[10] West and colleagues suggest one possible workflow model to address these concerns.[47] PGData use within HIV care may also impact patients' levels of responsibility, autonomy and control over their healthcare relative to HCPs. [38] [48] Thus, it seems plausible



For peer review only - http://bmjopen.bmj.com/site/about/guidelines.xhtml

that PGData will impact relationships between people living with HIV and HCPs. The extent of this impact is presently unknown, but it may vary in its effect on both patients and HCPs, as well as across settings and cultural contexts.

Data privacy, security and reliability

Although mobile apps were the most-used digital platform, across the literature such apps have received repeated criticism for lacking regulation and approval for medical use, potentially threatening the quality, validity and reliability of mobile-collected data. [37] [49] [50] [51] [52] Furthermore, many apps lack privacy policies or data encryption measures, risking data exploitation, theft or unauthorised access. [49] [53] [54] Given this review's prominent PGData privacy and security concerns, without adequate regulatory, privacy and security protocols people living with HIV may be less inclined to engage with PGData. Furthermore, both data security mistrust and social desirability, wherein patients may under or over-report actions to appear more socially acceptable, may increase the likelihood of deliberate PGData manipulation, degrading its reliability. [48] [55] Ultimately, PGData adoption into HIV care may be jeopardised if concerns regarding safety, privacy and trustworthiness prevail. [49]

Limitations

The small article yield has several possible explanations. Many articles were out of scope and considered ineligible; inconsistencies in PGData terminology across the literature [56] may have exacerbated this and potentially rendered relevant articles unidentifiable using the current review's search strategy. Excluding conference proceedings, coupled with challenges in locating full-text articles, may mean some studies were overlooked. Consequently, the structured search strategy proved less fruitful in yielding articles relative to manual reference-searching, a finding other authors have observed. [57] Additionally, author repetition across the studies calls into question whether the same, or similarly-recruited, samples were used between studies, raising further generalisability concerns.



For peer review only - http://bmjopen.bmj.com/site/about/guidelines.xhtml

As the majority of articles involved hypothetical or pilot platforms, they lack ecological validity and applicability. Additionally, the heterogeneity of PGData platforms used prevents their direct comparison and evaluation. Although the digital platforms addressed were biased to mobile phones, this reflects the wider digital health literature and this platform is more established than other recent technological developments (e.g. wearable devices). [40] Finally, although PGData use may require training, the one training study [25] involved such high training intensity that its real-world feasibility is unlikely.

**Future research recommendations**

Based upon this review's findings, the following four areas warrant further research:

1. Real-world and longitudinal evidence

Resulting from the absence of real-world, longitudinal evidence of PGData use in HIV care, a full-scale pragmatic trial is necessary to enhance the research's ecological validity and enable greater insight into practical issues surrounding PGData usage within HIV care.

2. Impact of PGData use upon clinical outcomes

The direct relationship between PGData use and clinical outcomes in people living with HIV, such as medication adherence, quality of life or mental wellbeing, remains unknown and requires investigation.

3. Digital platforms and PGData types

The effectiveness and optimal digital platform type(s) for PGData collection and monitoring requires evaluation. Additionally, exploration of the most useful types of PGData to track is needed.

4. Different contexts and HIV populations

Research exploring PGData use within HIV care in more diverse populations of people living with HIV is critically needed to increase the literature's representativeness for



For peer review only - http://bmjopen.bmj.com/site/about/guidelines.xhtml

HIV key populations and low and middle-income settings. Additionally, a greater understanding of how PGData use may fluctuate over time is needed. Future research must improve the scope of geographical and patient contexts to avoid further magnifying research inequities regarding PGData use within HIV care. [5] [58]

## CONCLUSIONS

The emerging PGData field presents opportunities to improve patient self-management and personalised care, which are both increasingly needed due to health system resource constraints and the complexities of HIV care. In recent months, HIV care, services and people living with HIV worldwide have been negatively impacted by the COVID-19 pandemic, causing decreased HIV testing, disrupted antiretroviral treatment procurement and poorer psychological wellbeing of people living with HIV. [59] [60] However, the pandemic has highlighted the value of, and significant shift towards, digital healthcare delivery, [61] [62] which may provide expedited leverage for further PGData research. Going forwards, the real-world applicability of PGData within HIV care must be elucidated, including navigating the heterogeneity and optimal suitability of PGData platforms for people living with HIV within different contexts. Additionally, PGData integration within HIV care will be conditional upon adequately addressing outstanding privacy, data security, reliability and HCP-patient relationship concerns prior to implementation. [14] [55] Overall, PGData may hold the potential to transform HIV care, in turn realising improvements in supporting not only people living with HIV but also the wider health systems in which they are cared for.



For peer review only - http://bmjopen.bmj.com/site/about/guidelines.xhtml

- **Contributors**: CH, KCL, ST, AD, and JG contributed to the conception and/or design of the study. CH led, and KCL and JG contributed to the establishment of the search strategy and method of analysis. Article reviewing and data analysis was led by CH with contribution from KCL and JG. CH drafted the original manuscript with supervision and guidance from JG, KL and ST. All authors have made contributions to the drafting and revising of the article, and have approved the final version.

- **Funding**: This research received no specific grant from any funding agency in the public, commercial or not-for-profit sectors. All authors (with the exception of Clara Hewitt) receive research funding from the INTUIT (Interaction Design for Trusted Sharing of Personal Health Data to Live Well with HIV) research project, which is funded by the Engineering and Physical Sciences Research Council (EP/R033900/1).

- **Data availability statement:** This manuscript reports on a scoping review. All articles included in the sample are included in the article and/or as supplementary material.
- **Competing interests:** None declared.
- **Patient consent:** Not applicable.
- **Ethics approval:** Not applicable.



For peer review only - http://bmjopen.bmj.com/site/about/guidelines.xhtml

## REFERENCES

[1] Deeks, S. G., Lewin, S. R. & Havlir, D. V. (2013). The end of AIDS: HIV infection as a chronic disease. *The Lancet*, 382 (9903): pp. 1525-1533.
[2] Claborn, K. R., Fernandez, A., Wray, T. & Ramsey S. (2015). Computer-based HIV adherence promotion interventions: a systematic review. *Translation Behavioral Medicine*, 5 (3): pp. 294–306.
[3] Hunt, P. W. (2014). HIV and aging: Emerging research issues. *Current Opinion in HIV and AIDS*, 9 (4): pp. 302–308.
[4] Marcus, J. L., Leyden, W. A., Alexeeff, S. E., Anderson, A. N., Hechter, R. C., Hu, H., Lam, J. O., Towner, W. J., Yuan ,Q., Horberg, M. A. & Silverberg, M. J. (2020). Comparison of Overall and Comorbidity-Free Life Expectancy Between Insured Adults With and Without HIV Infection, 2000-2016. *JAMA Network* Open, 3 (6): e207954.
[5] Claborn, K. R., Meier, E., Miller, M. B., Leavens, E. L., Brett, E. I. & Leffingwell, T. (2018). Improving adoption and acceptability of digital health interventions for HIV disease management: a qualitative study. *Translational Behavioral Medicine,* 8 (2): pp. 268-279.
[6] Cooper, V., Clatworthy, J. & Whetham, J. (2017). mHealth Interventions To Support Self- Management In HIV: A Systematic Review. *The Open AIDS Journal,* 11: pp. 119-132.
[7] Nittas, V., Mütsch, M., Ehrler, F. & Puhan, M. A. (2018). Electronic patient-generated health data to facilitate prevention and health promotion: a scoping review protocol. *BMJ Open,* 8: pp. e021245.
[8] Park, Y. R., Lee, Y., Kim, J. Y., Kim, J., Kim, H. R., Kim, Y. H., Kim, W. S. & Lee, J. H. (2018). Managing Patient-Generated Health Data Through Mobile Personal Health Records: Analysis of Usage Data. *JMIR Mhealth and Uhealth*, 6 (4): pp. e89.
[9] Shapiro, M., Johnston, D., Wald, J. & Mon, D. (2012). Patient-Generated Health Data. White Paper prepared for Office of Policy and Planning Office of the National Coordinator for Health Information Technology. Available from: https://www.healthit.gov/sites/default/files/rti_pghd_whitepaper_april_2012.pdf. [Accessed 5th June 2019].
[10] Reading, M. & Merrill, J. (2018). Converging and diverging needs between patients and providers who are collecting and using patient-generated health data: An integrative review. *Journal of the American Medical Informatics Association,* 25 (6): pp. 759-771.
[11] Wood, W. A., Bennett, A. V. & Basch, E. (2015). Emerging uses of patient generated health data in clinical research. *Molecular Oncology*, 9 (5): pp. 1018–1024.
[12] Cohen, D. J., Keller, S. R., Hayes, G. R., Dorr, D. A., Ash, J. S. & Sittig, D. F. (2016). Integrating Patient-Generated Health Data into Clinical Care Settings or Clinical Decision-Making: Lessons Learned from Project HealthDesign. *JMIR Human Factors*, 3 (2): pp. e26.
[13] Chung, A. E. & Basch, E. M. (2015). Potential and Challenges of Patient-Generated Health Data for High-Quality Cancer Care. *Journal of Oncology Practice,* 11 (3): pp. 195-197.
[14] Murthy, H. & Wood, S. (2015). The Value of Patient Reported Outcomes and Other Patient-Generated Health Data in Clinical Hematology. *Current Hematologic Malignancy Reports,* 10 (3): pp. 213-224.
[15] Burns, K. (2017). Engagement, empowerment and patient generated health data. PhD thesis, Queensland University of Technology. Available from: https://eprints.qut.edu.au/114076/1/Kara%20Burns%20Thesis.pdf.



For peer review only - http://bmjopen.bmj.com/site/about/guidelines.xhtml

[16] Zhu, H., Colgan, J., Reddy, M. & Choe, E. K. (2017). Sharing Patient-Generated Data in Clinical Practices: An Interview Study. *AMIA Annual Symposium Proceedings Archive*. 2016: pp. 1303–1312.
[17] Torkamani, A., Andersen, K. G., Steinhubl, S. R. & Topol, E. J. (2017). High-definition medicine. *Cell,* 170 (5): pp. 828-843.
[18] Nundy, S., Lu, C.-Y. E., Hogan, P., Mishra, A. & Peek, M. E. (2014). Using Patient-Generated Health Data from Mobile Technologies for Diabetes Self-Management Support: Provider Perspectives from an Academic Medical Center. *Journal of Diabetes Science and Technology*, 8 (1): pp. 74-82.
[19] Pham et al., 2014 Pham, M. T., Rajić, A., Greig, J. D., Sargeant, J. M., Papadopoulos, A., & McEwen, S. A. (2014). A scoping review of scoping reviews: advancing the approach and enhancing the consistency. *Research synthesis methods*, *5*(4), 371–385
[20] Tricco, A. C., Lillie, E., Zarin, W., O'Brien, K. K., Colquhoun, H., Levac, D., Moher, D., Peters, M., Horsley, T., Weeks, L., Hempel, S., Akl, E. A., Chang, C., McGowan, J., Stewart, L., Hartling, L., Aldcroft, A., Wilson, M. G., Garritty, C., Lewin, S., Godfrey, C. M., Macdonald, M. T., Lanlois, E. V., Soares-Weiser, K., Moriarty, J., Clifford, T., Tunçalp, Ö & Straus, S. E. (2018). PRISMA Extension for Scoping Reviews (PRISMA-ScR): Checklist and Explanation. *Annals of Internal Medicine*, 169 (7): pp. 467–473. Available from: https://www.equator-network.org/reporting-guidelines/prisma-scr/
[21] Hong, Q. N., Pluye, P., Fàbregues, S., Bartlett, G., Boardman, F., Cargo, M., Dagenais, P., Gagnon, M.-P., Griffiths, F., Nicolau, B., O'Cathain, A., Rousseau, M.-C. & Vedel, I. (2018). Mixed Methods Appraisal Tool Version 2018 User Guide. Available from: http://mixedmethodsappraisaltoolpublic.pbworks.com/w/file/fetch/127916259/MMAT_2018_criteria-manual_2018-08-01_ENG.pdf. [Accessed 10 June 2019].
[22] Braun, V. & Clarke, V. (2006). Using thematic analysis in psychology. *Qualitative Research in Psychology*. 3 (2): pp. 77-101.
[23] Wheeldon, J. & Åhlberg, M. K. (2012). Mapping mixed-methods research: theories, models, and measures. In Wheeldon, J., & Åhlberg, M. K. *Visualizing Social Science Research: Maps, Methods, & Meaning* (pp. 113-148). Thousand Oaks, CA: SAGE Publications, Inc.
[24] Swendeman, D., Farmer, S., Mindry, D., Lee, S. J. & Medich, M. (2016). HIV Care Providers' Attitudes regarding Mobile Phone Applications and Web-Based Dashboards to support Patient Self-Management and Care Coordination: Results from a Qualitative Feasibility Study. *Journal of HIV and AIDS*, 2 (4): pp. 10.
[25] Luque, A. E., Corales, R., Fowler, R. J., Dimarco, J., Van Keken, A., Winters, P., Keefer, M. C. & Fiscella, K. (2013). Bridging the Digital Divide in HIV Care: A Pilot Study of an iPod Personal Health Record. *Journal of the International Association of Providers of AIDS Care (JIAPAC),* 12 (2): pp. 117-121
[26] Odlum, M., Gordon, P., Camhi, E., Valdez, E. & Bakken, S. (2014). Exploring Factors Related to the Adoption and Acceptance of an Internet-based Electronic Personal Health Management Tool (EPHMT) in a Low Income, Special Needs Population of People Living with HIV and AIDS in New York City, *Studies in Health Technology and Informatics,* 201 (1): pp. 145-152.
[27] Nokes, K., Verkuilen, J., Hickey, D., James-Borga, J. & Shan, J. (2013). Developing a personal health record self-efficacy tool. *Applied Nursing Research,* 26 (1): pp. 32-9.
[28] Schnall, R., Bakken, S., Brown Iii, W., Carballo-Dieguez, A. & Iribarren, S. (2016). Usabilty Evaluation of a Prototype Mobile App for Health Management for Persons Living with HIV. *Studies in Health Technology and Informatics*, 225 (1): pp. 481–485.



For peer review only - http://bmjopen.bmj.com/site/about/guidelines.xhtml

[29] Stonbraker, S., Cho, H., Hermosi, G., Pichon, A. & Schnall, R. (2018). Usability Testing of a mHealth App to Support Self-Management of HIV-Associated Non-AIDS Related Symptoms. *Studies in Health Technology and Informatics*, 250 (1): pp. 106–110.
[30] Ramanathan, N., Swendeman, D., Comulada, W. S., Estrin, D. & Rotheram-Borus, M. J. (2013). Identifying preferences for mobile health applications for self-monitoring and self-management: Focus group findings from HIV-positive persons and young mothers. *International Journal of Medical Informatics,* 82 (4): pp. 38-46.
[31] Schnall, R., Gordon, P., Camhi, E. & Bakken, S. (2011). Perceptions of factors influencing use of an electronic record for case management of persons living with HIV. *AIDS Care,* 23 (3): pp. 357-365.
[32] DeSilva, M. B., Gifford, A. L., Xu, K., Zhong, L., Feng, C., Brooks, M., Harrold, M., Yueying, H., Gill, C. J., Wubin, X., Vian, T., Haberer, J., Bangsberg, D. & Sabin, L. (2013). *AIDS Research and Treatment,* Article ID: 957862.
[33] Bussone, A. (2018). Reflection and personal health informatics for people living with HIV. PhD Thesis, City University of London.
[34] Marent, B., Henwood, F. & Darking, M. (2018). Development of an mHealth platform for HIV Care: Gathering User Perspectives Through Co-Design Workshops and Interviews. *JMIR mHealth and uHealth*, 6 (10): pp. e184.
[35] Fettig, J., Swaminathan, M., Murrill, C. S. & Kaplan, J. E. (2014). Global epidemiology of HIV. *Infectious Disease Clinics of North America*. 28 (3): pp. 323-337.
[36] Robinson, L., Cotten, S., Ono, H., Quan-Haase, A., Mesch, G., Chen, W., Schulz, J., Hale, T. M. & Stern, M. (2015). Digital inequalities and why they matter. *Information, Communication & Society,* 18 (5): pp. 1-14.
[37] Mehraeen, E., Safdari, R., Mohammadzadeh, N., Seyedalinaghi, S., Forootan, S. & Mohran, M. (2018). Mobile-Based Applications and Functionalities for Self-Management of People Living with HIV. *Health Informatics Meets eHealth,* 248: pp. 172-179.
[38] Lupton, D. & Maslen, S. (2019). How Women Use Digital Technologies for Health: Qualitative Interview and Focus Group Study. *Journal of Medical Internet Research*, 21 (1): pp. e11481.
[39] Singh, A., Gibbs, J., Estcourt, C., Sonnenberg, P. & Blandford, A. (2017). DH '17: Proceedings of the 2017 International Conference on Digital Health July 2017 Pages 6–15. DOI: https://doi.org/10.1145/3079452.3079469.
[40] Piwek, L., Ellis, D. A., Andrews, S. & Joinson, A. (2016). The Rise of Consumer Health Wearables: Promises and Barriers. *PLoS Medicine,* 13 (2): pp. e1001953.
[41] McAuley, A. (2014). Digital health interventions: widening access or widening inequalities? *Public Health,* 128 (12): pp. 1118-1120.
[42] Jongbloed, K., Parmar, S., van der Kop, M., Spittal, P. M., & Lester, R. T. (2015). Recent Evidence for Emerging Digital Technologies to Support Global HIV Engagement in Care. *Current HIV/AIDS reports*, 12 (4): pp. 451–461.
[43] Beaunoyer, E., Dupéré, S. & Guitton, M. J. (2020). COVID-19 and digital inequalities: Reciprocal impacts and mitigation strategies. *Computers in Human Behaviour,* 111.
[44] Watts, G. (2020). COVID-19 and the digital divide in the UK. *The Lancet Digital Health,* 2 (8): E395-396.
[45] Liu, A., Coleman, K., Bojan, K., Serrano, P. A., Oyedele, T., Garcia, A., Enriquez-Bruce, E., Emmanuel, P., Jones, J., Sullivan, P., Hightow-Weidman, L., Buchbinder, S. & Scott, H. (2019). Developing a Mobile App (LYNX) to Support Linkage to HIV/Sexually Transmitted Infection Testing and Pre-Exposure Prophylaxis for Young Men Who Have Sex with Men: Protocol for a Randomized Controlled Trial. *JMIR research Protocols*, 8 (1): pp. e10659.



For peer review only - http://bmjopen.bmj.com/site/about/guidelines.xhtml

[46] McGraw, D., Belfort, R., Pfister, H. & Ingargiola, S. (2013). Going digital with patients: Managing potential liability risks of patient-generated electronic health information. *Journal of Participatory Medicine*. 18 (5): pp. e41. Available from: http://www.jopm.org/perspective/narratives/2013/12/18/going-digital-with-patients-managing-potential-liability-risks-of-patient-generated-electronic-health-information. [Accessed 20 July 2019].
[47] West, P., Van Kleek, M., Giordano, R., Weal, M. J. & Shadbolt, N. (2018). CHI '18: Proceedings of the 2018 CHI Conference on Human Factors in Computing SystemsApril 2018 Paper No.: 484 Pages 1–13, DOI: https://doi.org/10.1145/3173574.3174058
[48] Huba, N. & Zhang, Y. (2012). Designing patient-centred personal health records (PHRs): Health care professionals' perspective on patient-generated data. *Journal of Medical Systems,* 36 (6): pp. 3893-3905.
[49] Torous, J., Roberts, L. W. (2017). Needed Innovation in Digital Health and Smartphone Applications for Mental Health: Transparency and Trust. *JAMA Psychiatry*, 74 (5): pp. 437-438.
[50] Effy, V., Tobias, H., Afua, A. & Alessandro, B. (2018). Digital health: meeting the ethical and policy challenges. *Swiss Medical Weekly,* 148: pp. w14571.
[51] Huckvale, K., Prieto, J. T., Tilney, M., Benghozi, P. J. & Car, J. (2015). Unaddressed privacy risks in accredited health and wellness apps: a cross-sectional systematic assessment. *BMC Medicine*, 13: pp. 214.
[52] Gibbs, J., Gkatzidou, V., Tickle, L., Manning, S. R., Tilakkumar, T., Hone, K., Ashcroft, R. E., Sonnenberg, P., Sadiq, S. T. & Estcourt, C. S. (2017). 'Can you recommend any good STI apps?' A review of content, accuracy and comprehensiveness of current mobile medical applications for STIs and related genital infections. *Sexually Transmitted Infections,* 93 (4): pp. 234-235.
[53] Beauclair, R., Meng, F., Deprez, N., Temmerman, M., Welte, A., Hens, N. & Delva, W. (2013). Evaluating audio computer assisted self-interviews in urban south African communities: Evidence for good suitability and reduced social desirability bias of a cross-sectional survey on sexual behaviour. *BMC Medical Research Methodology,* 13 (1): pp.11.
[54] Powell, A. C., Landman, A. B. & Bates, D. W. In search of a few good apps. (2014). *JAMA,* 311 (18): pp. 1851-1852.
[55] Charani, E., Castro-Sánchez, E., Moore, L. S. & Holmes, A. (2014). Do smartphone applications in healthcare require a governance and legal framework? It depends on the application! *BMC Medicine,* 12 (1): pp. 29.
[56] Lordon, R. J., Mikles, S. P., Kneale, L., Evans, H. L., Munson, S. A., Backonja, U. & Lober, W. B. (2020). How patient-generated health data and patient-reported outcomes affect patient-clinician relationships: A systematic review. *Health Informatics Journal,* 1460458220928184
[57] Greenhalgh, T. & Peacock, R. (2005). Effectiveness and efficiency of search methods in systematic reviews of complex evidence: audit of primary sources. *BMJ,* 331: pp. 1064
[58] Sinha, C. & Schryer-Roy, A. M. (2018). Digital health, gender and health equity: invisible imperatives. (2018). *Journal of Public Health (Oxford)*. 40 (2): pp. 21-25.
[59] Jiang, H., Zhou, Y. & Tang, W. (2020). Maintining HIV care during the COVID-19 pandemic. *The Lancet HIV,* 7 (5): pp. e308-e309.
[60] Kowalska, J. D., Skrzat-Klapaczyńska, A., Bursa, D., Balayan, T., Begovac, J., Chkhartishvili, N., Gokengin, D., Harxhi, A., Jilich, D., Jevtovic, D., Kase, K., Lakatos, B., Matulionyte, R., Mulabdic, V., Nagit, A., Papadopoulos, A., Stefanovic, M., Vassilenko, A., Vasylyev, M., Yancheva, N., Yurin, O. & Horban, A., for the ECEE Network Group. (2020). HIV care in times of the COVID-19 crisis - Where are we now in Central and Eastern Europe? *International Journal of Infectious Diseases,* 96: pp. 311–314.



For peer review only - http://bmjopen.bmj.com/site/about/guidelines.xhtml

[61] Watson, A. R., Wah, R. & Thamman, R. (2020). The Value of Remote Monitoring for the COVID-19 Pandemic. *Telemedicine and e-Health. Ahead of print.*
[62] Peek, N., Sujan, M. & Scott, P. (2020). Digital health and care in pandemic times: impact of COVID-19. *BMJ Health & Care Informatics,* 27: e100166.



For peer review only - http://bmjopen.bmj.com/site/about/guidelines.xhtml

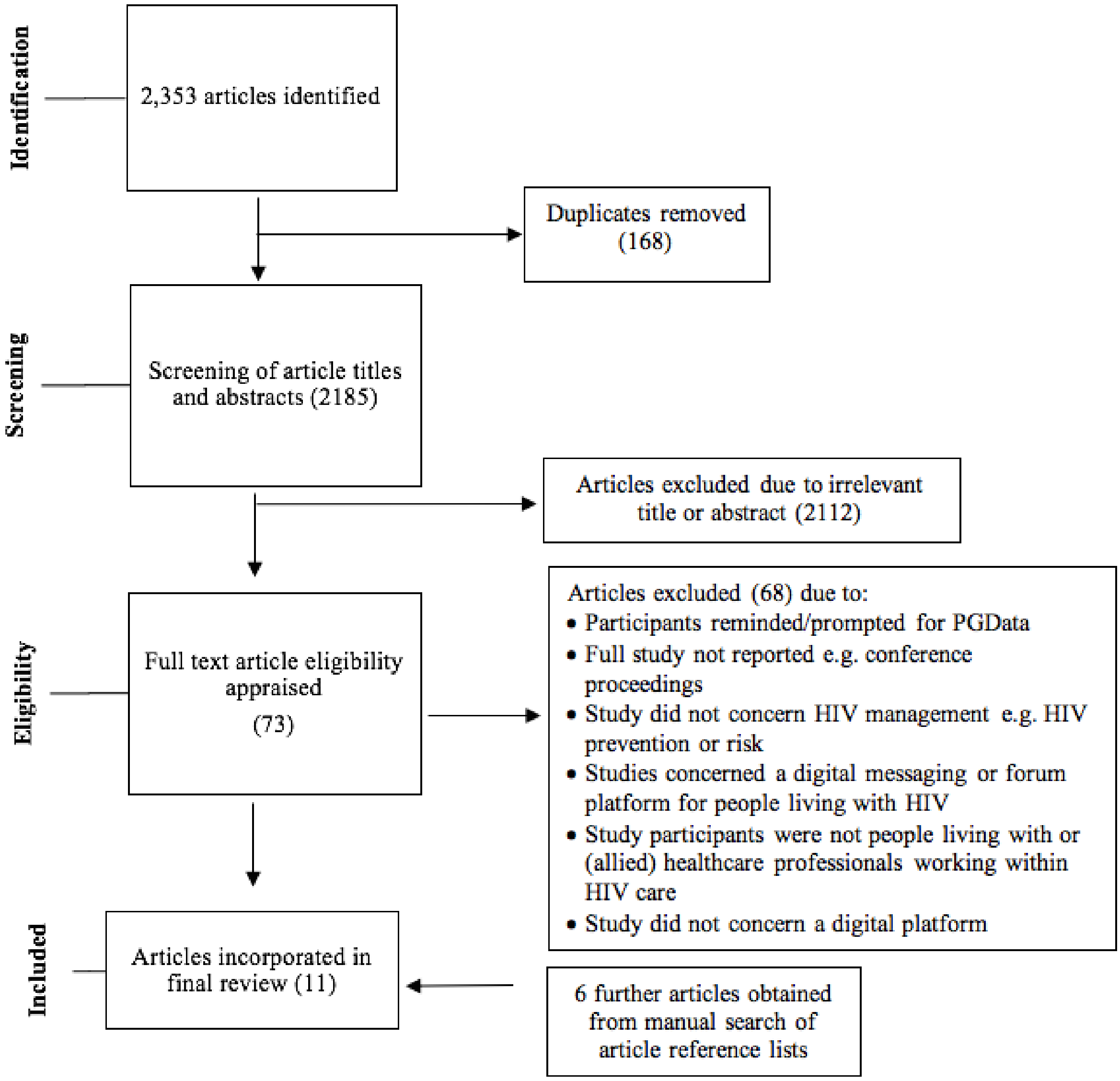


**Figure 1.** ***PRISMA flow diagram.***

**Preferred Reporting Items for Systematic reviews and Meta-Analyses extension for Scoping Reviews (PRISMA-ScR) Checklist**

| SECTION | ITEM | PRISMA-ScR CHECKLIST ITEM | REPORTED ON PAGE # |
|---|---|---|---|
| **TITLE** | | | |
| Title | 1 | Identify the report as a scoping review. | |
| **ABSTRACT** | | | |
| Structured summary | 2 | Provide a structured summary that includes (as applicable): background, objectives, eligibility criteria, sources of evidence, charting methods, results, and conclusions that relate to the review questions and objectives. | |
| **INTRODUCTION** | | | |
| Rationale | 3 | Describe the rationale for the review in the context of what is already known. Explain why the review questions/objectives lend themselves to a scoping review approach. | |
| Objectives | 4 | Provide an explicit statement of the questions and objectives being addressed with reference to their key elements (e.g., population or participants, concepts, and context) or other relevant key elements used to conceptualize the review questions and/or objectives. | |
| **METHODS** | | | |
| Protocol and registration | 5 | Indicate whether a review protocol exists; state if and where it can be accessed (e.g., a Web address); and if available, provide registration information, including the registration number. | |
| Eligibility criteria | 6 | Specify characteristics of the sources of evidence used as eligibility criteria (e.g., years considered, language, and publication status), and provide a rationale. | |
| Information sources* | 7 | Describe all information sources in the search (e.g., databases with dates of coverage and contact with authors to identify additional sources), as well as the date the most recent search was executed. | |
| Search | 8 | Present the full electronic search strategy for at least 1 database, including any limits used, such that it could be repeated. | |
| Selection of sources of evidence† | 9 | State the process for selecting sources of evidence (i.e., screening and eligibility) included in the scoping review. | |
| Data charting process‡ | 10 | Describe the methods of charting data from the included sources of evidence (e.g., calibrated forms or forms that have been tested by the team before their use, and whether data charting was done independently or in duplicate) and any processes for obtaining and confirming data from investigators. | |
| Data items | 11 | List and define all variables for which data were sought and any assumptions and simplifications made. | |
| Critical appraisal of individual sources of evidence§ | 12 | If done, provide a rationale for conducting a critical appraisal of included sources of evidence; describe the methods used and how this information was used in any data synthesis (if appropriate). | |
| Synthesis of results | 13 | Describe the methods of handling and summarizing the data that were charted. | |



For peer review only - http://bmjopen.bmj.com/site/about/guidelines.xhtml

| SECTION | ITEM | PRISMA-ScR CHECKLIST ITEM | REPORTED ON PAGE # |
|---|---|---|---|
| **RESULTS** | | | |
| Selection of sources of evidence | 14 | Give numbers of sources of evidence screened, assessed for eligibility, and included in the review, with reasons for exclusions at each stage, ideally using a flow diagram. | |
| Characteristics of sources of evidence | 15 | For each source of evidence, present characteristics for which data were charted and provide the citations. | |
| Critical appraisal within sources of evidence | 16 | If done, present data on critical appraisal of included sources of evidence (see item 12). | |
| Results of individual sources of evidence | 17 | For each included source of evidence, present the relevant data that were charted that relate to the review questions and objectives. | |
| Synthesis of results | 18 | Summarize and/or present the charting results as they relate to the review questions and objectives. | |
| **DISCUSSION** | | | |
| Summary of evidence | 19 | Summarize the main results (including an overview of concepts, themes, and types of evidence available), link to the review questions and objectives, and consider the relevance to key groups. | |
| Limitations | 20 | Discuss the limitations of the scoping review process. | |
| Conclusions | 21 | Provide a general interpretation of the results with respect to the review questions and objectives, as well as potential implications and/or next steps. | |
| **FUNDING** | | | |
| Funding | 22 | Describe sources of funding for the included sources of evidence, as well as sources of funding for the scoping review. Describe the role of the funders of the scoping review. | |

JBI = Joanna Briggs Institute; PRISMA-ScR = Preferred Reporting Items for Systematic reviews and Meta-Analyses extension for Scoping Reviews.

* Where *sources of evidence* (see second footnote) are compiled from, such as bibliographic databases, social media platforms, and Web sites.

† A more inclusive/heterogeneous term used to account for the different types of evidence or data sources (e.g., quantitative and/or qualitative research, expert opinion, and policy documents) that may be eligible in a scoping review as opposed to only studies. This is not to be confused with *information sources* (see first footnote).

‡ The frameworks by Arksey and O'Malley (6) and Levac and colleagues (7) and the JBI guidance (4, 5) refer to the process of data extraction in a scoping review as data charting.

§ The process of systematically examining research evidence to assess its validity, results, and relevance before using it to inform a decision. This term is used for items 12 and 19 instead of "risk of bias" (which is more applicable to systematic reviews of interventions) to include and acknowledge the various sources of evidence that may be used in a scoping review (e.g., quantitative and/or qualitative research, expert opinion, and policy document).

*From:* Tricco AC, Lillie E, Zarin W, O'Brien KK, Colquhoun H, Levac D, et al. PRISMA Extension for Scoping Reviews (PRISMAScR): Checklist and Explanation. Ann Intern Med. 2018;169:467–473. doi: 10.7326/M18-0850.



For peer review only - http://bmjopen.bmj.com/site/about/guidelines.xhtml

**Appendix B-** Search terms used within the literature search strategy.

Keywords for each subject heading of ‘HIV’ and ‘patient-generated health data’ respectively were combined using the search command ‘OR’. Articles yielded within each of these searches were combined using the search command ‘AND’ to produce the final article results.

## Keywords used

***Subject heading: HIV***

HIV OR human immunodeficiency virus OR HIV/AIDS OR HIV-infected OR HIV-associated OR HIV-positive OR HIV positive OR HIV+ OR people living with HIV OR persons living with HIV OR HIV patient* OR HIV infection

***Subject heading: Patient-generated health data***

Patient generated data OR patient-generated data OR patient generated health data OR patient-generated health data OR patient generated health information OR patient-generated health information OR personal health record* OR patient portal* OR personal data sharing OR patient collected data OR patient-collected data OR personal health informati* OR personal health data OR electronic health record* OR electronic personal health record* OR electronic health data OR self-recorded health data OR self-recorded data OR self-generated data or self-reported data OR patient collated data OR patient-collected data OR patient-captured data OR patient-controlled data OR patient-reported data OR electronic record.

An example complete database search strategy, for Medline, is shown below:

For peer review only

| # ▲ | Searches | Results | Type | Actions | Annotations |
|---|---|---|---|---|---|
| 1 | exp HIV Infections/ or HIV/ or exp HIV Long-Term Survivors/ | 277245 | Advanced | Display Results More ▾ | |
| 2 | (HIV or human immunodeficiency virus).mp. or HIV/aids or HIV-infected.mp. or HIV-associated.mp. or HIV-positive.mp. or HIV positive.mp. or HIV+.mp. or people living with HIV.mp. or persons living with HIV.mp. or HIV patient*.mp. or HIV infection.mp. [mp=title, abstract, original title, name of substance word, subject heading word, floating sub-heading word, keyword heading word, organism supplementary concept word, protocol supplementary concept word, rare disease supplementary concept word, unique identifier, synonyms] | 351883 | Advanced | Display Results More ▾ | |
| 3 | exp health records, personal/ or exp patient generated health data/ or exp patient portals/ | 1636 | Advanced | Display Results More ▾ | |
| 4 | (Patient generated data or patient-generated data or patient generated health data or patient-generated health data or patient generated health information or patient-generated health information or personal health record* or patient portal* or personal data sharing or patient collected data or patient-collected data or personal health informati* or personal health data or electronic health record* or electronic personal health record* or electronic health data or self-recorded health data or self-recorded data or self-generated data or self-reported data or patient collated data or patient-collected data or patient-captured data or patient-controlled data or patient-reported data or electronic record).mp. [mp=title, abstract, original title, name of substance word, subject heading word, floating sub-heading word, keyword heading word, organism supplementary concept word, protocol supplementary concept word, rare disease supplementary concept word, unique identifier, synonyms] | 28979 | Advanced | Display Results More ▾ | |
| 5 | 1 or 2 | 391186 | Advanced | Display Results More ▾ | |
| 6 | 3 or 4 | 29523 | Advanced | Display Results More ▾ | |
| 7 | 5 and 6 | 489 | Advanced | Display Results More ▾ | |

## Appendix C- Data extraction table of included articles

| Studies concerning a hypothetical digital platform for PGData collection | | | | | | | |
|---|---|---|---|---|---|---|---|
| **Authors (date)** | **Location** | **Participants** | **Study design** | **Length of study** | **Study aims and summary** | **Digital platform summary** | **Measures** |
| Bussone (2018, Study One of three) | England, UK | N = 16 people living with HIV<br><br>Age: range 18-57 years, M = 39.87<br><br>Gender: 8 male, 8 female<br><br>Ethnicity: 1 White British, 1 White other, White/Black African, 8 Black African, 2 Latin, 1 Asian Pakistani, 1 White/Black/Caribbean, 1 Mixed other<br><br>Orientation: 5 homosexual, 9 heterosexual, 1 bisexual, 1 asexual | Qualitative, individual semi-structured interviews of approximately 30 minutes | N/A | • **Aim**: to understand how people living with HIV monitor their personal health data<br>**Topics addressed:** participants' current self-management behaviours and issues surrounding current, previous or desired means of tracking personal health information | N/A as no specific digital platform used | Verbal feedback from participants regarding self-tracking of their personal health information |

| | | | | | | | |
|---|---|---|---|---|---|---|---|
| Marent, Henwood & Darking (2018) | Brighton (UK), Lisbon (Portugal), Barcelona (Spain), Antwerp (Belgium) and Zagreb (Croatia) | N = 160<br>• 97 people living with HIV<br>• 63 HCPs working in HIV care<br>-40 doctors<br>-10 nurses<br>-4 psychologists<br>-4 pharmacists<br>-2 social workers<br>-2 nutritionists<br>-1 sexologist<br><br>Age (of people living with HIV): range = 23-78 years<br><br>Gender (people living with HIV): 78 male<br><br>Ethnicity: authors report that people living with HIV had 14 different nationalities, with 13 identifying as belonging to a migrant community<br><br>Orientation (people living with HIV): 65 gay or lesbian, 26 heterosexual, 3 bisexual, 3 other. | Qualitative, 14 recorded workshops and 22 semi-structured interviews | January-June 2016 | • **Aim**: to determine perceptions of, and advantages and concerns surrounding, the development of a mobile app for HIV management<br>• **Topics addressed:** perceived challenges and barriers to use of a mobile app for HIV management, current mobile app usage and perceived useful features of a mobile app for HIV management | A hypothetical mobile app was discussed. | Verbal feedback from participants regarding factors related to a hypothetical mobile app for HIV management |

For peer review only

| | | | | | | | |
|---|---|---|---|---|---|---|---|
| Nokes et al. (2013) | New York, USA | N = 100 people living with HIV/AIDS<br><br>Age: M= 48.5<br><br>Gender: 94 male<br><br>Orientation: unreported<br><br>Ethnicity: 7 Asian/Pacific Islander, 51 African American/Black, 34 Hispanic/Latino, 1 Native American, 4 White/Anglo, 3 other<br><br>Education: 21 <11th grade, 45 high school/general education development, 25 2-years college, 9 4+ years of college | Quantitative | N/A | • **Aim:** to assess self-efficacy of people living with HIV regarding use of a hypothetical personal health record<br>• Participants received an explanation of a personal health record and completed questionnaires measuring their self-efficacy in relation to digital and paper-based personal health records, condom use and chronic disease management | N/A as no specific digital platform used | • Self-efficacy for creation and use of a paper-based personal health record<br>• Self-efficacy for creation and use of a digital personal health record<br>• Self-efficacy regarding medication adherence (HIV-Treatment Adherence Self-Efficacy Scale)<br>• Self-efficacy for chronic disease management scale<br>Self-efficacy for condom usage scale |

For peer review only

| | | | | | | | |
|---|---|---|---|---|---|---|---|
| Ramanathan et al. (2013) | Los Angeles, USA | N = 29 people living with HIV<br><br>Age: range= 30-60 years<br><br>Gender: 75% male<br><br>-Ethnicity: reported approximately one third of participants in each ethnic group of African American, Latino and White<br><br>Orientation: 55% men who have sex with men, 10% transgender | Qualitative, focus group discussions | N/A | • **Aim:** to assess feature preferences for a mobile app for HIV self-management<br>• **Topics addressed:** privacy, goal-setting, data capturing methods, feedback regarding app-user behaviours and the role of reminders | A hypothetical mobile app was discussed. | • Verbal feedback regarding key issues pertaining to development of a mobile app for HIV self-management |

For peer review only

| | | | | | | | |
|---|---|---|---|---|---|---|---|
| Swendeman et al. (2016) | Los Angeles, USA | N = 12 HCPs from a Los Angeles County HIV clinic<br>• 3 doctors<br>• 1 nurse practitioner<br>• 2 nurse case managers<br>• 2 psychosocial case managers<br>• 1 psychosocial case manager<br>• 1 psychotherapist<br><br>Age: unreported<br><br>Gender: unreported<br><br>Ethnicity: unreported<br><br>Orientation: unreported<br><br>Education: unreported | Qualitative, individual 45-60 minute semi-structured interviews | N/A | • **Aim:** to assess HCP attitudes regarding a hypothetical mobile app (for people living with HIV) and online dashboards (for HCPs) for HIV management<br>• **Topics addressed:** usability, acceptability and barriers to digital platform use | A hypothetical mobile app and online dashboard were discussed. | • Verbal feedback from participants regarding the use of hypothetical web-based dashboards for HCPs and hypothetical mobile apps for people living with HIV, both to facilitate self-management in patients |

| **Studies concerning a novel digital platform for PGData collection** | | | | | | | |
|---|---|---|---|---|---|---|---|
| Schnall et al. (2011) | New York, USA | N = 37 Case Managers working with people living with HIV<br><br>Age: unreported<br><br>Gender: unreported<br><br>Ethnicity: unreported<br><br>Orientation: unreported | Qualitative, one-hour focus group discussions | March-December 2008 | • **Aim:** to determine factors influencing usage and acceptability of online "SelectHealth Continuity of Care Document" for people living with HIV, before the platform's release<br>• **Topics addressed:** facilitators and barriers to use of the continuity of care record | The "SelectHealth Continuity of Care Document" includes key health information including latest medical test results, pharmaceutical record, visits to healthcare facilities and patient contact information | • Verbal feedback regarding perceived facilitators and barriers to use of the "SelectHealth Continuity of Care Document" |
| Schnall et al. (2016) | New York, USA | N = 15<br>• 10 people living with HIV<br>• 5 informaticians<br><br>Age: unreported<br><br>Gender (people living with HIV): 6 male<br><br>Ethnicity: unreported<br><br>Orientation: unreported | Mixed: quantitative, qualitative | [illegible] | • **Aim:** to test a prototype mobile app designed to facilitate HIV management in people living with HIV Participants received a description of a mobile app prototype for HIV management<br>• Informaticians tested the model for 45-90 minutes and completed a Heuristic Evaluation Checklist<br>• people living with HIV evaluated prototype app screenings and completed the Post Study System Usability Questionnaire | No description of mobile app prototype provided | • For informaticians: score on Heuristic Evaluation Checklist<br>• For people living with HIV: score on Post Study System Usability Questionnaire as a measurement of the app's usability |

| | | | | | | | | |
|---|---|---|---|---|---|---|---|---|
| Stonbraker et al. (2018) | New York, USA | N= 25<br>• 20 people living with HIV who possessed at least one HIV-associated non-AIDS condition<br>• 5 informaticians<br><br>Age: M= 54.4 years<br><br>Gender: 40% male<br><br>Ethnicity: only reported 85% African American<br><br>Orientation: unreported<br><br>Education: 50% <high school, 35% high school, 15% more than high school | Mixed: quantitative, qualitative | N/A | • **Aim:** to determine usability of novel mobile app *VIP-HANA* (Video Information Provider for HIV-associated non-AIDS conditions) designed to aid people living with HIV with management of HIV/non-HIV-related symptoms, and to determine where improvements to the app were needed<br>• After receiving descriptions of the app participants were assigned tasks on a beta version of the app and had to describe aloud their thoughts and actions whilst their activities were tracked<br>• people living with HIV completed one questionnaire to assess their health literacy level, and two questionnaires relating to the app's usability | • Within the app individuals must indicate which HIV-associated non-AIDS conditions they possess out of nine options<br>• Individuals can then categorise associated symptoms of the condition(s) out of 28 options, according to their severity<br>Upon severity categorisation, the app-user receives tailored strategies to facilitate self-management behaviours | • For informaticians: assigned mobile app task activity and score on Heuristic Evaluation Checklist<br>• For people living with HIV: assigned mobile app task activity, score on the Newest Vital Sign assessment (to determine health literacy level) and scores on two questionnaires relating to app usability (Health Information Technology Usability Evaluation Scale and Post Study System Usability Questionnaire) |

For peer review only

**Studies concerning an existing, established digital platform for PGData collection**

| Authors (date) | Location | Participants | Study design | Length of study | • Study aims and summary | • Digital platform summary | • Measures |
|---|---|---|---|---|---|---|---|
| DeSilva et al. (2013) | Nanning, China | N= 10 people living with HIV who were injection drug users<br><br>Age: M = 32.7 years<br><br>Gender: 7 male<br><br>Orientation: unreported<br><br>Ethnicity: unreported<br><br>Education: 9 participants <middle school | Qualitative, individual interviews before and after using the device | One month | • **Aim:** to determine the feasibility and acceptability of using a *Wisepill* device in injecting drug users who were HIV positive<br>• Participants used the *Wisepill* device to monitor their antiretroviral medication adherence for one month<br>• **Topics addressed:** acceptability, feasibility and usability of the device | • The *Wisepill* container stores medication<br>• Upon opening, a wireless signal is immediately transmitted to a server, the data of which can be obtained by HCPs<br>• Opening the *Wisepill* container provides a proxy measurement of medication adherence | • Verbal feedback from participants regarding feasibility, acceptability and usability of the *Wisepill* device to track medication adherence<br>• Self-reported adherence to antiretroviral medication after one month of using the *Wisepill* device |

| | | | | | | | |
|---|---|---|---|---|---|---|---|
| Luque et al. (2013) | Rochester, New York, USA | N = 29 people living with HIV<br><br>Age: M= 48<br><br>Gender: 55% male, 41% female, 3% transgender<br><br>Ethnicity: 41% African American, 21% Latino, 28% white, 10% other<br><br>Orientation: unreported<br><br>Education level: 31% <high school, 28 % high school or equivalent, 41% >high school<br><br>Annual household income: 83% <$20,000, 17% >$20,000<br><br>Use of electronic technology: 18% never used a computer, 21% never used the internet, 36% never used email | Quantitative | 6 weeks | • **Aim:** to determine acceptability and usability of a personal health record on an iPod touch device, and to observe the effect of using a digital personal health record upon HIV treatment self-efficacy<br>• Participants underwent six 90-minute training sessions and were given 'homework' tasks to rehearse between training sessions | • The *MyMedical* app, (downloaded from iTunes Store) was trialled as a personal health record on an iPod touch device<br><br>• No more information regarding the app was provided within the study | • HIV-Treatment Adherence Self-Efficacy Scale (baseline and one month post-training)<br>• Post-training questionnaire score for iPod device usability<br>• Post-training questionnaire score for personal health record usability<br>• Attitudes Towards Computers Scale |

For peer review only

| | | | | | | | |
|---|---|---|---|---|---|---|---|
| Odlum et al. (2014) | New York, USA | N= 57 people living with HIV<br>• Quantitative: 41 people living with HIV<br>-M age= 47 years, range 24-63 years<br><br>-Gender: 27 male<br><br>-Ethnicity: 27 Black/African American, 11 other/mixed race<br><br>-Orientation: unreported<br><br>-Education: 25 <high school diploma, 4 associate degrees, 2 Bachelor's degrees<br><br>• Qualitative: 15 people living with HIV,<br>8 users of the *MyHealthProfile* app<br><br>-Gender: 2 male<br><br>-Ethnicity: 7 Black African American, 1 Latino<br><br>-Orientation: unreported | Mixed: quantitative (System Usefulness Survey), qualitative (focus groups of 1 hour) | N/A | • **Aim:** to determine usability of the *MyHealthProfile* app and to determine where improvements could be made to inform a new version of the app named *MyHealthProfile-plus*<br>• Participants completed 2 surveys relating to the usefulness and content of the pre-established *My Health Profile* app<br>• Participants completed 60 minute focus groups regarding the development of new *My Health Profile-plus* app | • The *MyHealthProfile* app acts as a continuity of care document for people living with HIV to track their personal health record via an internet connection | • System Usefulness Survey<br>• Ease of Use Scale<br>• App usefulness score<br>• App components score<br>• Verbal feedback regarding perceived app needs for novel app *My Health Profile-plus* |

For peer review only

| | | | | | | | |
|---|---|---|---|---|---|---|---|
| | | 7 non-users of the *MyHealthProfile* app<br>-Gender: 5 male<br>-Ethnicity: 6 Black African American, 1 Caucasian<br><br>-Orientation: unreported | | | | | |

For peer review only

## Appendix D- Results from the Mixed Methods Appraisal Tool assessment

**Bussone (2018)**

| | | Responses | | |
|---|---|---|---|---|
| **Study design** | **Methodological quality criteria** | **Yes** | **No** | **Can't tell** |
| **Screening questions (all studies)** | S1. Are there clear research questions? | X | | |
| | S2. Do the collected data allow to address the research questions? | X | | |
| **Qualitative** | 1.1. Is the qualitative approach appropriate to answer the research question? | X | | |
| | 1.2. Are the qualitative data collection methods adequate to address the research question? | X | | |
| | 1.3. Are the findings adequately derived from the data? | X | | |
| | 1.4. Is the interpretation of results sufficiently substantiated by data? | X | | |
| | 1.5. Is there coherence between qualitative data sources, collection, analysis and interpretation? | X | | |

For peer review only

**DeSilva et al. (2013)**

| | | Responses | | |
|---|---|---|---|---|
| **Study design** | **Methodological quality criteria** | **Yes** | **No** | **Can't tell** |
| **Screening questions (all studies)** | S1. Are there clear research questions? | X | | |
| | S2. Do the collected data allow to address the research questions? | X | | |
| **Qualitative** | 1.1. Is the qualitative approach appropriate to answer the research question? | X | | |
| | 1.2. Are the qualitative data collection methods adequate to address the research question? | X | | |
| | 1.3. Are the findings adequately derived from the data? | | | X |
| | 1.4. Is the interpretation of results sufficiently substantiated by data? | | | X |
| | 1.5. Is there coherence between qualitative data sources, collection, analysis and interpretation? | | | X |

**Luque et al. (2013)**

| | | Responses | | |
|---|---|---|---|---|
| **Study design** | **Methodological quality criteria** | **Yes** | **No** | **Can't tell** |
| **Screening questions (all studies)** | S1. Are there clear research questions? | X | | |
| | S2. Do the collected data allow to address the research questions? | X | | |
| **Quantitative descriptive** | 4.1. Is the sampling strategy relevant to address the research question? | X | | |
| | 4.2. Is the sample representative of the target population? | | X | |
| | 4.3. Are the measurements appropriate? | X | | |
| | 4.4. Is the risk of nonresponse bias low? | X | | |
| | 4.5. Is the statistical analysis appropriate to answer the research question? | X | | |

For peer review only

**Marent, Henwood & Darking (2018)**

| | | Responses | | |
|---|---|---|---|---|
| **Study design** | **Methodological quality criteria** | **Yes** | **No** | **Can't tell** |
| **Screening questions (all studies)** | S1. Are there clear research questions? | X | | |
| | S2. Do the collected data allow to address the research questions? | X | | |
| **Qualitative** | 1.1. Is the qualitative approach appropriate to answer the research question? | X | | |
| | 1.2. Are the qualitative data collection methods adequate to address the research question? | X | | |
| | 1.3. Are the findings adequately derived from the data? | X | | |
| | 1.4. Is the interpretation of results sufficiently substantiated by data? | X | | |
| | 1.5. Is there coherence between qualitative data sources, collection, analysis and interpretation? | X | | |

For peer review only

**Nokes et al. (2013)**

| Study design | Methodological quality criteria | Responses | | |
|---|---|---|---|---|
| | | Yes | No | Can't tell |
| **Screening questions (all studies)** | S1. Are there clear research questions? | X | | |
| | S2. Do the collected data allow to address the research questions? | X | | |
| **Quantitative descriptive** | 4.1. Is the sampling strategy relevant to address the research question? | X | | |
| | 4.2. Is the sample representative of the target population? | X | | |
| | 4.3. Are the measurements appropriate? | X | | |
| | 4.4. Is the risk of nonresponse bias low? | X | | |
| | 4.5. Is the statistical analysis appropriate to answer the research question? | X | | |

For peer review only

**Odlum et al. (2014)**

| | | Responses | | |
|---|---|---|---|---|
| **Study design** | **Methodological quality criteria** | **Yes** | **No** | **Can't tell** |
| **Screening questions (all studies)** | S1. Are there clear research questions? | X | | |
| | S2. Do the collected data allow to address the research questions? | X | | |
| **Qualitative** | 1.1. Is the qualitative approach appropriate to answer the research question? | X | | |
| | 1.2. Are the qualitative data collection methods adequate to address the research question? | X | | |
| | 1.3. Are the findings adequately derived from the data? | X | | |
| | 1.4. Is the interpretation of results sufficiently substantiated by data? | X | | |
| | 1.5. Is there coherence between qualitative data sources, collection, analysis and interpretation? | X | | |
| **Quantitative descriptive** | 4.1. Is the sampling strategy relevant to address the research question? | | | X |

For peer review only

| | | | | |
|---|---|---|---|---|
| | 4.2. Is the sample representative of the target population? | | X | |
| | 4.3. Are the measurements appropriate? | | | X |
| | 4.4. Is the risk of nonresponse bias low? | | | X |
| | 4.5. Is the statistical analysis appropriate to answer the research question? | | | X |
| **Mixed methods** | 5.1. Is there an adequate rationale for using a mixed methods design to address the research question? | | X | |
| | 5.2. Are the different components of the study effectively integrated to answer the research question? | | X | |
| | 5.3. Are the outputs of the integration of qualitative and quantitative components adequately interpreted? | | X | |
| | 5.4. Are divergences and inconsistencies between quantitative and qualitative results adequately addressed? | | X | |
| | 5.5. Do the different components of the study adhere to the quality criteria of each tradition of the methods involved? | | | X |

For peer review only

**Ramanathan et al. (2013)**

| | | Responses | | |
|---|---|---|---|---|
| **Study design** | **Methodological quality criteria** | **Yes** | **No** | **Can't tell** |
| **Screening questions (all studies)** | S1. Are there clear research questions? | X | | |
| | S2. Do the collected data allow to address the research questions? | X | | |
| **Qualitative** | 1.1. Is the qualitative approach appropriate to answer the research question? | X | | |
| | 1.2. Are the qualitative data collection methods adequate to address the research question? | X | | |
| | 1.3. Are the findings adequately derived from the data? | X | | |
| | 1.4. Is the interpretation of results sufficiently substantiated by data? | X | | |
| | 1.5. Is there coherence between qualitative data sources, collection, analysis and interpretation? | X | | |

For peer review only

**Swendeman et al. (2016)**

| | | Responses | | |
|---|---|---|---|---|
| **Study design** | **Methodological quality criteria** | **Yes** | **No** | **Can't tell** |
| **Screening questions (all studies)** | S1. Are there clear research questions? | X | | |
| | S2. Do the collected data allow to address the research questions? | X | | |
| **Qualitative** | 1.1. Is the qualitative approach appropriate to answer the research question? | X | | |
| | 1.2. Are the qualitative data collection methods adequate to address the research question? | X | | |
| | 1.3. Are the findings adequately derived from the data? | X | | |
| | 1.4. Is the interpretation of results sufficiently substantiated by data? | X | | |
| | 1.5. Is there coherence between qualitative data sources, collection, analysis and interpretation? | X | | |

For peer review only

**Schnall et al. (2011)**

| | | Responses | | |
|---|---|---|---|---|
| **Study design** | **Methodological quality criteria** | **Yes** | **No** | **Can't tell** |
| **Screening questions (all studies)** | S1. Are there clear research questions? | X | | |
| | S2. Do the collected data allow to address the research questions? | X | | |
| **Qualitative** | 1.1. Is the qualitative approach appropriate to answer the research question? | X | | |
| | 1.2. Are the qualitative data collection methods adequate to address the research question? | X | | |
| | 1.3. Are the findings adequately derived from the data? | X | | |
| | 1.4. Is the interpretation of results sufficiently substantiated by data? | X | | |
| | 1.5. Is there coherence between qualitative data sources, collection, analysis and interpretation? | X | | |

For peer review only

**Schnall et al. (2016)**

| | | Responses | | |
|---|---|---|---|---|
| **Study design** | **Methodological quality criteria** | **Yes** | **No** | **Can't tell** |
| **Screening questions (all studies)** | S1. Are there clear research questions? | X | | |
| | S2. Do the collected data allow to address the research questions? | | X | |
| **Qualitative** | 1.1. Is the qualitative approach appropriate to answer the research question? | | | X |
| | 1.2. Are the qualitative data collection methods adequate to address the research question? | | | X |
| | 1.3. Are the findings adequately derived from the data? | | | X |
| | 1.4. Is the interpretation of results sufficiently substantiated by data? | | | X |
| | 1.5. Is there coherence between qualitative data sources, collection, analysis and interpretation? | | | X |
| **Quantitative descriptive** | 4.1. Is the sampling strategy relevant to address the research question? | | X | |

For peer review only

| | | | | |
|---|---|---|---|---|
| | 4.2. Is the sample representative of the target population? | | X | |
| | 4.3. Are the measurements appropriate? | | | X |
| | 4.4. Is the risk of nonresponse bias low? | | | X |
| | 4.5. Is the statistical analysis appropriate to answer the research question? | | | N/A |
| **Mixed methods** | 5.1. Is there an adequate rationale for using a mixed methods design to address the research question? | | X | |
| | 5.2. Are the different components of the study effectively integrated to answer the research question? | | X | |
| | 5.3. Are the outputs of the integration of qualitative and quantitative components adequately interpreted? | | X | |
| | 5.4. Are divergences and inconsistencies between quantitative and qualitative results adequately addressed? | | X | |
| | 5.5. Do the different components of the study adhere to the quality criteria of each tradition of the methods involved? | | X | |

For peer review only

**Stonbraker et al. (2018)**

| | | Responses | | |
|---|---|---|---|---|
| **Study design** | **Methodological quality criteria** | **Yes** | **No** | **Can't tell** |
| **Screening questions (all studies)** | S1. Are there clear research questions? | X | | |
| | S2. Do the collected data allow to address the research questions? | X | | |
| **Qualitative** | 1.1. Is the qualitative approach appropriate to answer the research question? | | | X |
| | 1.2. Are the qualitative data collection methods adequate to address the research question? | | | X |
| | 1.3. Are the findings adequately derived from the data? | X | | |
| | 1.4. Is the interpretation of results sufficiently substantiated by data? | X | | |
| | 1.5. Is there coherence between qualitative data sources, collection, analysis and interpretation? | X | | |
| **Quantitative descriptive** | 4.1. Is the sampling strategy relevant to address the research question? | | | X |

For peer review only

| | | | | |
|---|---|---|---|---|
| | 4.2. Is the sample representative of the target population? | | X | |
| | 4.3. Are the measurements appropriate? | | | X |
| | 4.4. Is the risk of nonresponse bias low? | | | X |
| | 4.5. Is the statistical analysis appropriate to answer the research question? | | | X |
| **Mixed methods** | 5.1. Is there an adequate rationale for using a mixed methods design to address the research question? | | X | |
| | 5.2. Are the different components of the study effectively integrated to answer the research question? | | X | |
| | 5.3. Are the outputs of the integration of qualitative and quantitative components adequately interpreted? | | X | |
| | 5.4. Are divergences and inconsistencies between quantitative and qualitative results adequately addressed? | | X | |
| | 5.5. Do the different components of the study adhere to the quality criteria of each tradition of the methods involved? | | X | |

For peer review only

## Preferred Reporting Items for Systematic reviews and Meta-Analyses extension for Scoping Reviews (PRISMA-ScR) Checklist

| SECTION | ITEM | PRISMA-ScR CHECKLIST ITEM | REPORTED ON PAGE # |
|---|---|---|---|
| **TITLE** | | | |
| Title | 1 | Identify the report as a scoping review. | |
| **ABSTRACT** | | | |
| Structured summary | 2 | Provide a structured summary that includes (as applicable): background, objectives, eligibility criteria, sources of evidence, charting methods, results, and conclusions that relate to the review questions and objectives. | |
| **INTRODUCTION** | | | |
| Rationale | 3 | Describe the rationale for the review in the context of what is already known. Explain why the review questions/objectives lend themselves to a scoping review approach. | |
| Objectives | 4 | Provide an explicit statement of the questions and objectives being addressed with reference to their key elements (e.g., population or participants, concepts, and context) or other relevant key elements used to conceptualize the review questions and/or objectives. | |
| **METHODS** | | | |
| Protocol and registration | 5 | Indicate whether a review protocol exists; state if and where it can be accessed (e.g., a Web address); and if available, provide registration information, including the registration number. | |
| Eligibility criteria | 6 | Specify characteristics of the sources of evidence used as eligibility criteria (e.g., years considered, language, and publication status), and provide a rationale. | |
| Information sources* | 7 | Describe all information sources in the search (e.g., databases with dates of coverage and contact with authors to identify additional sources), as well as the date the most recent search was executed. | |
| Search | 8 | Present the full electronic search strategy for at least 1 database, including any limits used, such that it could be repeated. | |
| Selection of sources of evidence† | 9 | State the process for selecting sources of evidence (i.e., screening and eligibility) included in the scoping review. | |
| Data charting process‡ | 10 | Describe the methods of charting data from the included sources of evidence (e.g., calibrated forms or forms that have been tested by the team before their use, and whether data charting was done independently or in duplicate) and any processes for obtaining and confirming data from investigators. | |
| Data items | 11 | List and define all variables for which data were sought and any assumptions and simplifications made. | |
| Critical appraisal of individual sources of evidence§ | 12 | If done, provide a rationale for conducting a critical appraisal of included sources of evidence; describe the methods used and how this information was used in any data synthesis (if appropriate). | |
| Synthesis of results | 13 | Describe the methods of handling and summarizing the data that were charted. | |

For peer review only

| SECTION | ITEM | PRISMA-ScR CHECKLIST ITEM | REPORTED ON PAGE # |
|---|---|---|---|
| **RESULTS** | | | |
| Selection of sources of evidence | 14 | Give numbers of sources of evidence screened, assessed for eligibility, and included in the review, with reasons for exclusions at each stage, ideally using a flow diagram. | |
| Characteristics of sources of evidence | 15 | For each source of evidence, present characteristics for which data were charted and provide the citations. | |
| Critical appraisal within sources of evidence | 16 | If done, present data on critical appraisal of included sources of evidence (see item 12). | |
| Results of individual sources of evidence | 17 | For each included source of evidence, present the relevant data that were charted that relate to the review questions and objectives. | |
| Synthesis of results | 18 | Summarize and/or present the charting results as they relate to the review questions and objectives. | |
| **DISCUSSION** | | | |
| Summary of evidence | 19 | Summarize the main results (including an overview of concepts, themes, and types of evidence available), link to the review questions and objectives, and consider the relevance to key groups. | |
| Limitations | 20 | Discuss the limitations of the scoping review process. | |
| Conclusions | 21 | Provide a general interpretation of the results with respect to the review questions and objectives, as well as potential implications and/or next steps. | |
| **FUNDING** | | | |
| Funding | 22 | Describe sources of funding for the included sources of evidence, as well as sources of funding for the scoping review. Describe the role of the funders of the scoping review. | |

JBI = Joanna Briggs Institute; PRISMA-ScR = Preferred Reporting Items for Systematic reviews and Meta-Analyses extension for Scoping Reviews.

* Where *sources of evidence* (see second footnote) are compiled from, such as bibliographic databases, social media platforms, and Web sites.

† A more inclusive/heterogeneous term used to account for the different types of evidence or data sources (e.g., quantitative and/or qualitative research, expert opinion, and policy documents) that may be eligible in a scoping review as opposed to only studies. This is not to be confused with *information sources* (see first footnote).

‡ The frameworks by Arksey and O'Malley (6) and Levac and colleagues (7) and the JBI guidance (4, 5) refer to the process of data extraction in a scoping review as data charting.

§ The process of systematically examining research evidence to assess its validity, results, and relevance before using it to inform a decision. This term is used for items 12 and 19 instead of "risk of bias" (which is more applicable to systematic reviews of interventions) to include and acknowledge the various sources of evidence that may be used in a scoping review (e.g., quantitative and/or qualitative research, expert opinion, and policy document).

*From:* Tricco AC, Lillie E, Zarin W, O'Brien KK, Colquhoun H, Levac D, et al. PRISMA Extension for Scoping Reviews (PRISMAScR): Checklist and Explanation. Ann Intern Med. 2018;169:467–473. doi: 10.7326/M18-0850.



For peer review only

BMJ Open

# Patient-generated data in the management of HIV: a scoping review

| | |
|---|---|
| Journal: | *BMJ Open* |
| Manuscript ID | bmjopen-2020-046393.R1 |
| Article Type: | Original research |
| Date Submitted by the Author: | 08-Mar-2021 |
| Complete List of Authors: | Hewitt, Clara; University College London, Institute for Global Health<br>Lloyd, Karen; University College London, Centre for Population Research in Sexual Health and HIV, Institute for Global Health<br>Tariq, Shema; University College London, Institute for Global Health; University College London<br>Durrant, Abigail; Newcastle University, Open Lab, School of Computing<br>Claisse, Caroline; Newcastle University, Open Lab, School of Computing<br>Kasadha, Bakita; Terrence Higgins Trust<br>Gibbs, Jo; University College London, Research Department of Infection and Population Health; |
| <b>Primary Subject Heading</b>: | HIV/AIDS |
| Secondary Subject Heading: | Health informatics, Infectious diseases |
| Keywords: | HIV & AIDS < INFECTIOUS DISEASES, Telemedicine < BIOTECHNOLOGY & BIOINFORMATICS, GENITOURINARY MEDICINE |
| | |

SCHOLARONE™
Manuscripts

*I, the Submitting Author has the right to grant and does grant on behalf of all authors of the Work (as defined in the below author licence), an exclusive licence and/or a non-exclusive licence for contributions from authors who are: i) UK Crown employees; ii) where BMJ has agreed a CC-BY licence shall apply, and/or iii) in accordance with the terms applicable for US Federal Government officers or employees acting as part of their official duties; on a worldwide, perpetual, irrevocable, royalty-free basis to BMJ Publishing Group Ltd ("BMJ") its licensees and where the relevant Journal is co-owned by BMJ to the co-owners of the Journal, to publish the Work in this journal and any other BMJ products and to exploit all rights, as set out in our licence.*

*The Submitting Author accepts and understands that any supply made under these terms is made by BMJ to the Submitting Author unless you are acting as an employee on behalf of your employer or a postgraduate student of an affiliated institution which is paying any applicable article publishing charge ("APC") for Open Access articles. Where the Submitting Author wishes to make the Work available on an Open Access basis (and intends to pay the relevant APC), the terms of reuse of such Open Access shall be governed by a Creative Commons licence – details of these licences and which Creative Commons licence will apply to this Work are set out in our licence referred to above.*

*Other than as permitted in any relevant BMJ Author's Self Archiving Policies, I confirm this Work has not been accepted for publication elsewhere, is not being considered for publication elsewhere and does not duplicate material already published. I confirm all authors consent to publication of this Work and authorise the granting of this licence.*

For peer review only

**BMJ Open**

# Patient-generated data in the management of HIV: a scoping review

**Authors and affiliations**

Clara Hewitt[1]
Karen C Lloyd[1]
Shema Tariq[1]
Abigail Durrant[2]
Caroline Claisse[2]
Bakita Kasadha[3]
Jo Gibbs[1]

1 Institute for Global Health, University College London, UK
2 Open Lab, School of Computing, Newcastle University, Newcastle upon Tyne, UK
3 Terrence Higgins Trust, London, UK

**Correspondence to:**
Dr. Karen C. Lloyd, Mortimer Market Centre, Off Capper St, Bloomsbury, London WC1E 6JB; K.lloyd@ucl.ac.uk

**Word count** (excluding title page, abstract, tables, contributions and references): 3528

## ABSTRACT

**Objectives:** Patient-generated data (PGData) is an emergent research area and may improve HIV care. The objectives of this scoping review were to synthesise, evaluate and make recommendations based upon the available literature regarding PGData use in HIV care.

**Design:** Scoping review

**Data sources:** Embase, Medline, CINAHL Plus, Web of Science, Scopus, PsycINFO and Emcare databases.

**Eligibility Criteria:** Studies involving PGData use within HIV care for people living with HIV and/or healthcare professionals (HCPs) published before February 2021.

**Data extraction and synthesis:** Data were extracted using a table and the Mixed Methods Appraisal Tool was used to assess empirical rigour. We used Thematic Analysis to evaluate content.

**Results:** 11 articles met the eligibility criteria. Studies were observational, predominantly concerned hypothetical or novel digital platforms, mainly conducted in high-income settings, and had small sample sizes (range=10-160). There were multiple definitions of PGData. In the majority of studies (n=9) participants were people living with HIV, with a few studies including HCPs, informatics specialists, or mixed participant groups. Participants living with HIV were aged 23-78, mostly male, of diverse ethnicities, and had low educational, health literacy and income levels.
We identified four key themes: 1. Perceptions of PGData and associated digital platforms; 2. Opportunities; 3. Anticipated barriers; and 4. Potential impact upon patient-HCP relationships.**Conclusions:** Use of PGData within HIV care warrants further study, especially with regard to digital inequalities, data privacy and security. There is a need for longitudinal data on use within HIV in a variety of settings with a broad range of users, including impact on clinical outcomes. This will allow greater understanding of the role of PGData use in improving the health and wellbeing of people living with HIV, which is increasingly pertinent as digital healthcare becomes more widespread as a result of COVID-19.


**Keywords:** HIV; digital health; self-management; patient generated health data; remote monitoring.

**Strengths and limitations of this study:**

- This scoping review provides an overview and synthesis of the emergent literature of PGData use within HIV care, providing a foundation of understanding to inform future research.
- The Mixed Methods Appraisal Tool allowed assessment of mixed study designs.
- Literature searching yielded a limited number of articles, with most articles obtained via manual referencing.
- Only English studies were reviewed, which may bias and thus narrow the scope of the findings to English speaking settings.



For peer review only - http://bmjopen.bmj.com/site/about/guidelines.xhtml

Antiretroviral therapy (ART) has extended the life expectancy of people living with HIV, transforming HIV into a chronic condition. [1] Effective HIV care requires both long-term clinical follow-up as well as high levels of adherence to ART. This facilitates viral suppression, reducing HIV-related morbidity and mortality, as well as preventing onward HIV transmission. [2] However, HIV care is often complex; people living with HIV have a greater risk of co-morbid conditions such as poor mental health and cardiovascular disease compared to their HIV-negative counterparts. [3] [4] These co-morbidities (often a result of the virus, side effects of ART, and/or complex socioeconomic factors) may require considerable input from a wide range of healthcare professionals (HCPs), which has a consequential impact on healthcare resources, even in high income settings. [1] [5] With health systems already financially and time-poor, it is important to identify innovative approaches to HIV care that can support quality of life, while addressing the challenges posed by increasing co-morbidities and an ageing population. Digital technologies present opportunities through which to improve HIV care, and in turn, the health and quality of life of people living with HIV. [6]

The role of self-management in the care of long-term conditions such as HIV is becoming increasingly important. [6] The ubiquity of digital devices and applications used for health purposes can support self-management and has created fertile grounds for patient generated data (PGData) collection. [7] [8] Although no universal PGData definition exists, multiple authors across the literature have adopted the broad definition [9] that PGData constitute any health-related data which are created or collected by patients or designated proxies to address a health concern. [7] [10] [11] [12] [13] [14] As such, PGData include medication dosages, physical activity, dietary intake, sleep and mood patterns, and can be collected in different modalities including text, pictures, voice or video recordings, and numerical information e.g. questionnaire scores or physiological measurements. [11] [15] PGData allow



For peer review only - http://bmjopen.bmj.com/site/about/guidelines.xhtml

more frequent, remote and longitudinal tracking of multiple types of health information, and can be rapidly shared between patients and HCPs, or between patients themselves, usually using wireless connections. [13] [16] Additionally, PGData can serve as an adjunct to health information communicated during clinical consultations; they can demonstrate baseline health measures bespoke to each individual, which may ultimately facilitate more patient-centred, personalised care. [8] [14] [17] Consequently, PGData allow greater real-time insight into health fluctuations or anomalies relative to a baseline, permitting holistic appreciation of patient health for both patients and their HCPs, and potentially enabling more appropriate and timely interventions. [5] [9] [10] [11] [15] [16] [18]

Whilst the use of PGData for the management of other long-term conditions such as cancer [14] and diabetes [18] has been investigated, to our knowledge, no review of PGData use within HIV care has been conducted to date. This scoping review is timely, given that COVID-19 has led to rapidly increased adoption of remote healthcare and telemedicine practices within clinical care, which will likely remain in place beyond the current pandemic.

**Objectives:**

This scoping review aimed to 1. Synthesise and evaluate the existing emergent literature on PGData use within HIV care; 2. Identify the opportunities and challenges it presents; and 3. Consider the impact of PGData use upon people living with HIV and their HCPs. For the purposes of this review we interpreted 'HIV care' as being synonymous with HIV management, and to mean all HIV-related healthcare from the point of diagnosis.



For peer review only - http://bmjopen.bmj.com/site/about/guidelines.xhtml

# METHODS

We conducted a scoping review of literature on PGData use within HIV care. We adopted this approach to enable a broad focus and preliminary synthesis of available evidence [19] in this new, emerging research area. We have reported the review in accordance with the Preferred Reporting Items for Systematic Reviews and Meta-analysis extension for Scoping Reviews (PRISMA-ScR) guidelines [20] (see Appendix A). A review protocol has not been registered.

## Search strategy

During July 2019, we searched Medline, Embase, Emcare, CINAHL, PsycINFO, Scopus and Web of Science databases for articles. Additional grey literature databases were searched using the same strategy: National Institute of Clinical Excellence: Health and Social Care, TRIP medical database, ProQuest Dissertations and Theses, OpenAIRE, Bielefeld Academic Search Engine (BASE), OpenDOAR, Open Access Dissertations and Theses, DART-Europe, Open Grey and Semantic Scholar. Searches were restricted to English language and the terms used were adapted to meet the requirements of each database. Detailed search terms can be found in Appendix B. Article reference lists were also manually searched to obtain additional literature which the formal search strategy may have omitted.

CH conducted the original screenings in conjunction with JG and KL. ST and CH re-ran literature searches of all databases in February 2021 using the same search strategy.No further literature within scope was identified.



For peer review only - http://bmjopen.bmj.com/site/about/guidelines.xhtml

**Eligibility Criteria**

Study inclusion was contingent on the terms outlined in Table 1 and temporal limits were not imposed to keep the scope of literature generated as broad as possible given the infancy of the literature base.

We chose to exclude PGData which is collected in response to prompts from external sources e.g. mobile app or SMS reminders. Whilst PGdata prompts are not specifically addressed in the PGdata definition we employed, we made this exclusion on the basis that we believed PGData collected following prompts is not truly 'generated' autonomously by patients. We also employed this exclusion based on our consideration that prompted PGData may mean an individual has a qualitatively different experience of PGData collection and use, and that any prompted PGData collected may not be a true reflection of natural, uninfluenced patient engagement with PGdata e.g. the PGData which individuals would naturally collect unprompted.



For peer review only - http://bmjopen.bmj.com/site/about/guidelines.xhtml

| Inclusion Criteria |
|---|
| • Studies which considered the perspectives of HCPs or people living with HIV regarding PGData use within HIV management<br>• Studies where PGData use met the definition adopted [10]<br>• Where studies involved patients, these needed to be people living with HIV<br>• PGData collection by people living with HIV had to be conducted via a digital platform<br>• PGData collected in relation to living with HIV<br>• Any PGData created had to be collected independently by people living with HIV, and not in response to a reminder or prompt |
| **Exclusion Criteria** |
| • Studies involving PGData which were not generated independently by people living with HIV, or where PGData were created in response to the receipt of a reminder or prompt<br>• Studies concerning PGData use in the diagnosis, prevention or risk reduction of HIV<br>• Studies solely involving a two-way communication system, including studies involving digital messaging or forum platforms<br>• Conference proceedings |

***Table 1.*** **Summary of inclusion and exclusion criteria.**



For peer review only - http://bmjopen.bmj.com/site/about/guidelines.xhtml

## Selection of sources of evidence

The retrieved articles were exported into Mendeley Reference Manager and duplicates were removed. Titles and abstracts were screened, and full texts were assessed using the eligibility criteria. A data extraction table (see Appendix C) was used to summarise data from articles meeting the eligibility criteria for analysis.

## Empirical appraisal and thematic analysis of included articles

Due to heterogeneity of study designs, empirical quality was assessed using the Mixed Methods Appraisal Tool (MMAT, [21] see Appendix D). Thematic analysis [22] was conducted based upon the qualitative findings from the identified articles. We employed an inductive approach due to the nascent nature of this research field.

For the thematic analysis, primarily individual article-specific mind maps were created to highlight key concepts identified per article. [23] Subsequently, a comprehensive mind map was created to synthesise key topics across all 11 reviewed articles. Topics recurring twice or more between articles were highlighted as having thematic importance and these were finally organised into four overarching themes and three sub-themes. Themes were discussed by CH, KL, HG and ST until consensus was reached.

## Patient and public involvement

This manuscript reports on a review of the literature. As such, no patients or members of the public were explicitly involved in the scoping process, however, one co-author is a peer-researcher (with lived experience of HIV) within a third sector organisation.

# RESULTS

## Search results

Original literature searches were conducted in June 2019. A total of 2,353 articles were identified through academic databases and grey literature keyword searching (Figure 1). Seventy-three full-text articles were assessed, of which five fulfilled the eligibility criteria for



For peer review only - http://bmjopen.bmj.com/site/about/guidelines.xhtml

inclusion. A further six eligible studies were identified from a manual search of reference lists. A total of 11 articles were included for final review (see article summaries, Table 2). An updated search conducted in February 2021 did not identify any further eligible studies.

**[Figure 1. PRISMA Flow Diagram]**

**Participants and demographics**

Sample sizes of included studies ranged from 10-160 participants. Participants were predominantly people living with HIV, however, some studies involved HCPs, allied HCPs, informatics specialists, or mixed participant groups (see Appendix C). Apart from one study,[24] where nurses and doctors were participants, role specialisms were not identified. Where reported, studies primarily included men who have sex with men (MSM) and heterosexual men and women. The age range of people living with HIV across the studies was 23-78 years. Participants were mostly male and of Black (including Black African and African American) ethnicity. Only two studies involved transgender people living with HIV, despite this being a HIV key population. Where reported, people living with HIV were most often from low-income, low-educational backgrounds.

**Study characteristics**

The empirical quality of the 11 articles reviewed was varied (see MMAT assessment, Appendix D). Studies were predominantly conducted in the USA (eight studies [24] [25] [26] [27] [28] [29] [30] [31]), with others based in China [32], England [33] and other parts of Europe [34]. All PGData platforms were mobile or web-based except for one study involving a medication storage device. [32] Prior to analysis, articles were grouped into three categories according to the type of digital platform used; hypothetical, prototype or pre-established (see Table 2) in order to distinguish between the different natures of digital platform types. Hypothetical platforms were those where researchers had developed a PGData platform concept, with no tangible product in existence (5 studies), [24] [27] [30] [33] [34] prototype platforms involved



For peer review only - http://bmjopen.bmj.com/site/about/guidelines.xhtml

PGData platforms being piloted (3 studies), [28] [29] [31] and pre-established platforms referred to PGData platforms already in existence which were being trialled (3 studies). [25] [26] [32] Study designs included user testing, [25] [28] [29][32] focus groups [26] [30] [31] and semi-structured interview sessions. [24] [33] [34] One study also included patient-HCP co-design workshops. [34]

User testing studies varied in structure; two studies trialled beta mobile application (app) prototypes for PGData collection wherein people living with HIV and informaticians completed tasks whilst simultaneously voicing feedback, [28] [29] whilst others examined the impact of medication storage device use [32] and a six-week personal health record app training programme for people living with HIV. [25] User testing studies focused on the perceived usability and feasibility of PGData collection and associated digital platforms; some studies employed quantitative assessments to measure these [25] [28] [29] (see Appendix C). No studies conducted user testing with HCPs. Studies conducting interviews and focus group discussions tended to use open-ended questions or follow discussion guides, and centred around issues of PGData platform acceptability, feasibility, and anticipated barriers and facilitators to PGData adoption.

| **Studies concerning hypothetical digital platforms** | | | |
|---|---|---|---|
| ***Author(s)*** | ***Participants, design, location, study length*** | ***Digital platform*** | ***Overview*** |
| **Bussone (2018)** | -N = 16 people living with HIV<br>- Qualitative, individual semi-structured interviews of approximately 30 minutes<br>-England (UK)<br>-Study length unspecified | No specific digital platform used | Aimed to understand how people living with HIV monitor their personal health data. Addressed topics including participants' current self-management behaviours and issues surrounding current, previous or desired means of tracking personal health information. |



For peer review only - http://bmjopen.bmj.com/site/about/guidelines.xhtml

| | | | |
|---|---|---|---|
| **Marent, Henwood & Darking (2018)** | -N = 160 participants<br>• 97 people living with HIV<br>• 63 HCPs working in HIV care<br>-40 doctors<br>-10 nurses<br>-4 psychologists<br>-4 pharmacists<br>-2 social workers<br>-2 nutritionists<br>• 1 sexologist<br>- Qualitative, 14 recorded workshops and 22 semi-structured interviews<br>-Brighton (UK), Lisbon (Portugal), Barcelona (Spain), Antwerp (Belgium) and Zagreb (Croatia)<br>-January-June 2016 | No specific digital platform used | Aimed to determine perceptions of, and advantages and concerns surrounding, the development of a mobile app for HIV management. Addressed topics including perceived challenges and barriers to use of a mobile app for HIV management, current mobile app usage and perceived useful features of a mobile app for HIV management. |
| **Nokes et al. (2013)** | -N = 100 people living with HIV or AIDS<br>-Quantitative<br>-New York, USA<br>-Study length unspecified | No specific digital platform used | Aimed to assess self-efficacy of people living with HIV regarding use of a hypothetical personal health record. Participants received an explanation of a personal health record and completed questionnaires measuring their self-efficacy in relation to digital and paper-based personal health records, condom use and chronic disease management. |
| **Ramanathan et al. (2013)** | -N= 29 people living with HIV<br>-Qualitative: focus group discussions<br>-Los Angeles, USA<br>-Study length unspecified | No specific digital platform used | Aimed to assess feature preferences for a mobile app for HIV self-management. Addressed topics including privacy, goal-setting, data capturing methods, feedback regarding app-user behaviours and the role of reminders. |
| **Swendeman et al. (2016)** | - N = 12 HCPs working in HIV care<br>• 3 doctors<br>• 5 nurses<br>• 2 psychosocial case workers<br>• 1 outreach worker<br>• 1 psychotherapist<br>- Qualitative, individual 45-60 minute semi-structured interviews<br>-Los Angeles, USA<br>-Study length unspecified | No specific digital platform used | Aimed to assess HCP attitudes regarding a hypothetical mobile app (for people living with HIV) and online dashboards (for HCPs) for HIV management. Addressed topics including usability, acceptability and barriers to digital platform use. |
| **Studies concerning prototype digital platforms** | | | |

| *Author(s)* | *Participants, design, location, study length* | *Digital platform* | *Overview* |
|---|---|---|---|
| **Schnall et al. (2011)** | - N = 37 Case Managers working with people living with HIV<br>- Qualitative, one-hour focus group discussions<br>-New York, USA<br>- March-December 2008 | SelectHealth Continuity of Care Document; includes key health information including latest medical test results, pharmaceutical record, visits to healthcare facilities and patient contact information | Aimed to determine factors influencing usage and acceptability of online "SelectHealth Continuity of Care Document" for people living with HIV, before the platform's release. Addressed topics including facilitators and barriers to use of the continuity of care record. |
| **Schnall et al. (2016)** | -N = 15<br>• 10 people living with HVI<br>• 5 informaticians<br>- Mixed: quantitative, qualitative<br>-New York, USA<br>-Study length unspecified | No description of app prototype provided | Aimed to test a prototype mobile app designed to facilitate HIV management in people living with HIV. Participants received a description of a mobile app prototype for HIV management. Informaticians tested the model for 45-90 minutes and completed a Heuristic Evaluation Checklist<br>people living with HIV evaluated prototype app screenings and completed the Post Study System Usability Questionnaire. |
| **Stonbraker et al. (2018)** | -N= 25<br>• 20 people living with HIV who possessed at least one HIV-associated non-AIDS condition<br>• 5 informaticians<br>-Mixed: quantitative; qualitative<br>-New York, USA | -Novel mobile app "VIP-HANA" (Video Information Provider (VIP) for HIV-associated non-AIDS conditions) | Aimed to determine usability of novel mobile app "VIP-HANA" designed to aid people living with HIV with management of HIV/non-HIV-related symptoms, and to determine where improvements to the app were needed. After receiving descriptions of the app participants were assigned tasks on a beta version of the app and had to describe aloud their thoughts and actions whilst their activities were tracked. People living with HIV completed one questionnaire to assess their health literacy level, and two questionnaires relating to the app's usability. |
| **Studies concerning pre-established digital platforms** | | | |
| *Author(s)* | *Participants, design,*<br>*location, study length* | *Digital platform* | *Overview* |



For peer review only - http://bmjopen.bmj.com/site/about/guidelines.xhtml

| | | | |
|---|---|---|---|
| **DeSilva et al. (2012)** | -N= 10 people living with HIV who used injection drugs<br>-Individual qualitative interviews<br>-Nanning, China<br>-Study length unspecified | *Wisepill* medication storage container; once opened a wireless signal immediately transmits to a server, the data of which can be obtained by HCPs | Aimed to determine the feasibility and acceptability of using a "Wisepill" device in injecting drug users who were HIV positive. Participants used the "Wisepill" device to monitor their antiretroviral medication adherence for one month. Addressed topics including acceptability, feasibility and usability of the device. |
| **Luque et al. (2013)** | -N=29 people living with HIV<br>-Quantitative<br>-Rochester, New York, USA<br>-6 weeks | *MyMedical* app, (downloaded from iTunes Store). | Aimed to determine acceptability and usability of using the *MyMedical* app as a personal health record on an iPod touch device, and to observe the effect of using a digital personal health record upon HIV treatment self-efficacy. Participants underwent six 90-minute training sessions and were given 'homework' tasks to rehearse between training sessions. |
| **Odlum et al. (2014)** | -N= 57 people living with HIV<br>• Quantitative: 42 people living with HIV<br>• Qualitative: 15 people living with HIV; 8 users of the *MyHealthProfile* app, 7 non-users of the *MyHealthProfile* app<br>-Mixed: quantitative, qualitative<br>-New York, USA<br>-Study length unspecified | *MyHealthProfile* app; acts as a continuity of care people for living with HIV to track their personal health record via an internet connection | Aimed to determine usability of the *MyHealthProfile* app and to determine where improvements could be made to inform a new version of the app named *MyHealthProfile-plus*. Participants completed 2 surveys relating to the usefulness and content of the pre-established *My Health Profile* app. Participants completed 60 minute focus groups regarding the development of new *My Health Profile-plus* app |

***Table 2.*** **Summary of included articles**

## Overview of Key Themes

Our analysis highlighted four primary cross-cutting themes and three sub-themes, including: perceived acceptability, feasibility and usability of PGData; opportunities presented by PGData; anticipated barriers to PGData use; and how PGData may impact HIV care and HCP-patient relationships.

## Acceptability, feasibility and usability of digital platforms for PGData



For peer review only - http://bmjopen.bmj.com/site/about/guidelines.xhtml

Participants' views regarding the acceptability, feasibility and usability of PGData differed. Some people living with HIV were receptive to digitally tracking their PGData, though this was contingent upon data security. [30] Widespread smartphone access, absence of PGData platform cost, clear design and easy navigability of digital PGData platforms increased the perceived feasibility of PGData use. [24] [26] [32] [33]

Perceptions regarding the perceived suitability of PGData use for people living with HIV varied; some HCPs suggested PGData use would be better suited to newly-diagnosed individuals, those with multi-morbidities, or higher education levels [24] [34] whilst others proposed newly-diagnosed people living with HIV may be unsuitable. [34] Some HCPs questioned whether individual differences including motivation may affect PGData collection and engagement. [24] No data from people living with HIV regarding suitability were reported.

**Opportunities presented by PGData for HIV care**

Participants cited several perceived benefits of PGData use within HIV care, including reduced financial and time burdens, [31] convenience, and increased health proactivity. [26] [31] [33] Some people living with HIV perceived PGData collection could increase their medication adherence, perceived control, understanding and engagement surrounding their HIV care [33] due to their awareness of ongoing PGData monitoring. [24] [30] [32]

**Anticipated barriers and challenges associated with PGData**

Across the studies, several perceived challenges to PGData use emerged, including privacy and security, accessibility and reliability of PGData, and impact on HCPs.

Privacy and security

Participants expressed substantial privacy and security concerns regarding PGData collection and analysis, [25] [26] [30] [31] which some authors attributed to persisting HIV stigma.

[31] [32] [34] Participants voiced concerns that poor security and privacy may result in unwanted disclosures of sensitive data including HIV status, although how this might occur was not explicitly articulated. [30] [31][32] [34] Several people living with HIV emphasised desires for security mechanisms including encryption, [30] [33] [34] and some people living with HIV favoured paper-based PGData tracking methods due to digital platform apprehension. [33]

Accessibility and reliability of digital infrastructure and collected PGData

There were prevalent concerns regarding the reliability of digital platforms and PGData itself. Some studies reported concerns that technological disruptions and financial instability may hinder digital platform accessibility and usage. [24] [32] Additionally, some participants highlighted PGData reliability could be impaired by incorrect PGData recording. [31] Some people living with HIV warned of intentional retrospective editing to deliberately manipulate PGData. [30] [33]

Impact upon HCPs

HCPs were apprehensive that pre-existing time constraints and large workloads would hinder PGData-reviewing and that PGData involvement would only magnify these burdens. [24] [34] People living with HIV did not comment on the impact of PGData upon HCPs.

**Perceived impacts of PGData upon patient-HCP relationships**

Participants felt that engaging with PGData within clinical consultations could facilitate a more comprehensive picture of how a person may be managing their HIV. [24] [33] [34] Whilst people living with HIV desired PGData platforms allowing two-way communication with HCPs, HCPs feared increased workloads. [34]

Some people living with HIV thought that PGData use may provide opportunities to strengthen patient-HCP relationships. [34] However contrastingly, other participants expressed concern that PGData misinterpretation could impair pre-established face-to-face patient-HCP interactions. [30] [34] Both people living with HIV and HCPs believed PGData use could



For peer review only - http://bmjopen.bmj.com/site/about/guidelines.xhtml

complement, rather than supersede, traditional in-person HIV consultations, although details of such a hybrid model were absent. [34]

## DISCUSSION

This review provides a critical synthesis of the nascent literature on PGData use specifically for HIV care, and highlights several unanswered questions. Across the eleven articles, study designs and methodologies were varied and a spectrum of digital PGData platforms were considered. The views of people living with HIV and HCPs were broadly consistent, apart from on the potential impact of PGData upon patient-HCP relationships.

**Individual and contextual differences**

Our findings suggest that PGdata use may not be suitable for all people living with HIV; indeed, perceptions of PGData and its use within HIV care may differ greatly across patients and contexts. [35] For example, whilst some patients felt reassured by monitoring their long-term health condition, for others, health monitoring could be tiring, intrusive, anxiety-provoking and excessively time-consuming. [9][30] [36] [37] It is important that digital health interventions to support HIV care and self-management do not further reinforce stigma and use language that is accessible to all users. [38] As the majority of studies did not include participants from key HIV populations, such as women, people who are transgender, or people who inject drugs, knowledge of PGData use in these communities remains limited. [15] The current review's bias towards high-income settings further limits its generalisability given the global HIV burden lies predominantly in low and middle-income settings. [39] Furthermore, digital inequalities due to limited access and poor digital literacy [40] [41] will impact acceptability, feasibility and inequalities in PGData use, with the COVID-19 pandemic starkly highlighting these digital divides.[42] [43] Further research is needed to better understand these individual and contextual nuances.

### Impact upon patient-HCP relationships

Our findings highlight dichotomies between people living with HIV and HCPs; whilst people living with HIV appeared to welcome increased HCP contact and HCP-analysed PGData, HCPs expressed concerns around additional workload and expectations. [11] [14] [16] [24] [34] [44] [45] A PGData guidance framework may be necessary to counteract competing priorities between HCPs and patients regarding such communication preferences. [9] [12] West and colleagues suggest one possible workflow model to address these concerns.[46] PGData use within HIV care may also impact patients' levels of responsibility, autonomy and control over their healthcare relative to HCPs. [37] [47] Thus, it seems plausible that PGData will impact relationships between people living with HIV and HCPs. The extent of this impact is presently unknown, but it may vary in its effect on both patients and HCPs, as well as across settings and cultural contexts.

### Barriers to PGData uptake

Across the studies concerns from both patients and HCPs regarding the privacy, security and reliability of PGData were common, indicating considerable apprehension around the use of PGData. Regarding PGData platforms, in this review mobile apps were the most-used digital platform; however, more broadly, apps have received repeated criticism for lacking regulation and approval for medical use, potentially threatening the quality, validity and reliability of mobile-collected data. [36] [48] [49] [50] [51] [52] Furthermore, many apps lack privacy policies or data encryption measures, potentially risking data exploitation, theft or unauthorised access. [49] [53] [54]

Given the prominent PGData privacy and security concerns highlighted in this review, without adequate regulatory, privacy and security protocols, people living with HIV may be less inclined to engage with PGData. Furthermore, both data security mistrust and social desirability, wherein patients may under or over-report actions to appear more socially



For peer review only - http://bmjopen.bmj.com/site/about/guidelines.xhtml

acceptable, may increase the likelihood of deliberate PGData manipulation, degrading its reliability. [47] [53] Ultimately, PGData adoption into HIV care may be jeopardised if concerns regarding the safety, privacy and trustworthiness of the platforms used to collect PGData, or the PGData itself, prevail amongst people living with HIV and HCPs. [50] [54]

**Limitations**

Terminology and Search Strategy

The small article yield of 11 articles has several possible explanations. Many articles were out of scope and considered ineligible; inconsistencies in PGData terminology across the literature [55] may have exacerbated this and potentially rendered relevant articles unidentifiable using the current review's search strategy. Excluding conference proceedings, coupled with challenges in locating full-text articles, may mean some studies were overlooked. Consequently, the structured search strategy proved less fruitful in yielding articles relative to manual reference-searching, a finding other authors have observed. [56] Additionally, author repetition across the studies calls into question whether the same, or similarly-recruited, samples were used between studies, raising further generalisability concerns.

Lack of real-world evidence

The majority of studies were exploratory, involving hypothetical or pilot platforms, which limits their ecological validity and applicability. Additionally, the heterogeneity of PGData platforms used prevents their direct comparison and evaluation. Although the digital platforms addressed were biased to mobile phones, this reflects the wider digital health literature and this platform is more established than other recent technological developments (e.g. wearable devices). [36] Finally, although PGData use may require training, the one training study [25] involved such high training intensity that its real-world feasibility is unlikely.



For peer review only - http://bmjopen.bmj.com/site/about/guidelines.xhtml

**Future research recommendations**

Based upon this review, the following four areas warrant further research:

1. Real-world and longitudinal evidence

Resulting from the absence of real-world, longitudinal evidence of PGData use in HIV care, a full-scale pragmatic trial is necessary to enhance the research's ecological validity and enable greater insight into practical issues surrounding PGData usage within HIV care. The impact of any of the PGData platform categories we identified- pilot, hypothetical or novel- could be explored in trial settings.

2. Impact of PGData use upon clinical outcomes

The direct relationship between PGData use and clinical outcomes in people living with HIV, such as medication adherence, quality of life or mental wellbeing, remains unknown and requires investigation.

3. Digital platforms and PGData types

The effectiveness and optimal digital platform type(s) for PGData collection and monitoring requires evaluation. Additionally, exploration of the most useful types of PGData to track is needed.

4. Different contexts and HIV populations

Research exploring PGData use within HIV care in more diverse populations of people living with HIV is critically needed to increase the literature's representativeness for HIV key populations and low and middle-income settings. Additionally, a greater understanding of how PGData use may fluctuate over time is needed. Future research must improve the scope of geographical and patient contexts to avoid further magnifying research inequities regarding PGData use within HIV care. [5] [57]



For peer review only - http://bmjopen.bmj.com/site/about/guidelines.xhtml

## CONCLUSIONS

The emerging PGData field presents opportunities to improve patient self-management and personalised care, which are both increasingly needed due to health system resource constraints and the complexities of HIV care. In recent months, HIV care, services and people living with HIV worldwide have been negatively impacted by the COVID-19 pandemic, causing decreased HIV testing, disrupted antiretroviral treatment procurement and poorer psychological wellbeing of people living with HIV. [58] [59] However, the pandemic has highlighted the value of, and significant shift towards, digital healthcare delivery, [60] [61] which may provide expedited leverage for further PGData research. Going forwards, the real-world applicability of PGData within HIV care must be elucidated, including navigating the heterogeneity and optimal suitability of PGData platforms for people living with HIV within different contexts. Additionally, PGData integration within HIV care will be conditional upon adequately addressing outstanding privacy, data security, reliability and HCP-patient relationship concerns prior to implementation. [14] [62] Overall, PGData may hold the potential to transform HIV care, in turn realising improvements in supporting not only people living with HIV but also the wider health systems in which this care is provided.



For peer review only - http://bmjopen.bmj.com/site/about/guidelines.xhtml

- **Contributors**: CH, KCL, ST, AD, CC, BK and JG contributed to the conception and/or design of the study. CH led, and KCL and JG contributed to the establishment of the search strategy and method of analysis. Article reviewing and data analysis was led by CH with contribution from KCL and JG. CH drafted the original manuscript with supervision and guidance from JG, KCL and ST. All authors (CC, KCL, ST, AD, CC, BK and JG) have made contributions to the drafting and revising of the article, and have approved the final version.

- **Funding**: This research received no specific grant from any funding agency in the public, commercial or not-for-profit sectors. All authors (with the exception of Clara Hewitt) receive research funding from the INTUIT (Interaction Design for Trusted Sharing of Personal Health Data to Live Well with HIV) research project, which is funded by the Engineering and Physical Sciences Research Council (EP/R033900/1).

- **Data availability statement:** This manuscript reports on a scoping review. All articles included in the sample are included in the article and/or as supplementary material.
- **Competing interests:** None declared.
- **Patient consent:** Not applicable.
- **Ethics approval:** Not applicable.



For peer review only - http://bmjopen.bmj.com/site/about/guidelines.xhtml

# REFERENCES

[1] Deeks, S. G., Lewin, S. R. & Havlir, D. V. (2013). The end of AIDS: HIV infection as a chronic disease. *The Lancet*, 382 (9903): pp. 1525-1533.
[2] Claborn, K. R., Fernandez, A., Wray, T. & Ramsey S. (2015). Computer-based HIV adherence promotion interventions: a systematic review. *Translation Behavioral Medicine*, 5 (3): pp. 294–306.
[3] Hunt, P. W. (2014). HIV and aging: Emerging research issues. *Current Opinion in HIV and AIDS*, 9 (4): pp. 302–308.
[4] Marcus, J. L., Leyden, W. A., Alexeeff, S. E., Anderson, A. N., Hechter, R. C., Hu, H., Lam, J. O., Towner, W. J., Yuan ,Q., Horberg, M. A. & Silverberg, M. J. (2020). Comparison of Overall and Comorbidity-Free Life Expectancy Between Insured Adults With and Without HIV Infection, 2000-2016. *JAMA Network* Open, 3 (6): e207954.
[5] Claborn, K. R., Meier, E., Miller, M. B., Leavens, E. L., Brett, E. I. & Leffingwell, T. (2018). Improving adoption and acceptability of digital health interventions for HIV disease management: a qualitative study. *Translational Behavioral Medicine,* 8 (2): pp. 268-279.
[6] Cooper, V., Clatworthy, J. & Whetham, J. (2017). mHealth Interventions To Support Self- Management In HIV: A Systematic Review. *The Open AIDS Journal,* 11: pp. 119-132.
[7] Nittas, V., Mütsch, M., Ehrler, F. & Puhan, M. A. (2018). Electronic patient-generated health data to facilitate prevention and health promotion: a scoping review protocol. *BMJ Open,* 8: pp. e021245.
[8] Park, Y. R., Lee, Y., Kim, J. Y., Kim, J., Kim, H. R., Kim, Y. H., Kim, W. S. & Lee, J. H. (2018). Managing Patient-Generated Health Data Through Mobile Personal Health Records: Analysis of Usage Data. *JMIR Mhealth and Uhealth*, 6 (4): pp. e89.
[9] Shapiro, M., Johnston, D., Wald, J. & Mon, D. (2012). Patient-Generated Health Data. White Paper prepared for Office of Policy and Planning Office of the National Coordinator for Health Information Technology. Available from: https://www.healthit.gov/sites/default/files/rti_pghd_whitepaper_april_2012.pdf. [Accessed 5th June 2019].
[10] Reading, M. & Merrill, J. (2018). Converging and diverging needs between patients and providers who are collecting and using patient-generated health data: An integrative review. *Journal of the American Medical Informatics Association,* 25 (6): pp. 759-771.
[11] Wood, W. A., Bennett, A. V. & Basch, E. (2015). Emerging uses of patient generated health data in clinical research. *Molecular Oncology*, 9 (5): pp. 1018–1024.
[12] Cohen, D. J., Keller, S. R., Hayes, G. R., Dorr, D. A., Ash, J. S. & Sittig, D. F. (2016). Integrating Patient-Generated Health Data into Clinical Care Settings or Clinical Decision-Making: Lessons Learned from Project HealthDesign. *JMIR Human Factors*, 3 (2): pp. e26.
[13] Chung, A. E. & Basch, E. M. (2015). Potential and Challenges of Patient-Generated Health Data for High-Quality Cancer Care. *Journal of Oncology Practice,* 11 (3): pp. 195-197.
[14] Murthy, H. & Wood, S. (2015). The Value of Patient Reported Outcomes and Other Patient-Generated Health Data in Clinical Hematology. *Current Hematologic Malignancy Reports,* 10 (3): pp. 213-224.
[15] Burns, K. (2017). Engagement, empowerment and patient generated health data. PhD thesis, Queensland University of Technology. Available from: https://eprints.qut.edu.au/114076/1/Kara%20Burns%20Thesis.pdf.
[16] Zhu, H., Colgan, J., Reddy, M. & Choe, E. K. (2017). Sharing Patient-Generated Data in Clinical Practices: An Interview Study. *AMIA Annual Symposium Proceedings Archive*. 2016: pp. 1303–1312.



For peer review only - http://bmjopen.bmj.com/site/about/guidelines.xhtml

[17] Torkamani, A., Andersen, K. G., Steinhubl, S. R. & Topol, E. J. (2017). High-definition medicine. *Cell,* 170 (5): pp. 828-843.
[18] Nundy, S., Lu, C.-Y. E., Hogan, P., Mishra, A. & Peek, M. E. (2014). Using Patient-Generated Health Data from Mobile Technologies for Diabetes Self-Management Support: Provider Perspectives from an Academic Medical Center. *Journal of Diabetes Science and Technology*, 8 (1): pp. 74-82.
[19] Pham et al., 2014 Pham, M. T., Rajić, A., Greig, J. D., Sargeant, J. M., Papadopoulos, A., & McEwen, S. A. (2014). A scoping review of scoping reviews: advancing the approach and enhancing the consistency. *Research synthesis methods*, *5*(4), 371–385
[20] Tricco, A. C., Lillie, E., Zarin, W., O'Brien, K. K., Colquhoun, H., Levac, D., Moher, D., Peters, M., Horsley, T., Weeks, L., Hempel, S., Akl, E. A., Chang, C., McGowan, J., Stewart, L., Hartling, L., Aldcroft, A., Wilson, M. G., Garritty, C., Lewin, S., Godfrey, C. M., Macdonald, M. T., Lanlois, E. V., Soares-Weiser, K., Moriarty, J., Clifford, T., Tunçalp, Ö & Straus, S. E. (2018). PRISMA Extension for Scoping Reviews (PRISMA-ScR): Checklist and Explanation. *Annals of Internal Medicine*, 169 (7): pp. 467–473. Available from: https://www.equator-network.org/reporting-guidelines/prisma-scr/
[21] Hong, Q. N., Pluye, P., Fàbregues, S., Bartlett, G., Boardman, F., Cargo, M., Dagenais, P., Gagnon, M.-P., Griffiths, F., Nicolau, B., O'Cathain, A., Rousseau, M.-C. & Vedel, I. (2018). Mixed Methods Appraisal Tool Version 2018 User Guide. Available from: http://mixedmethodsappraisaltoolpublic.pbworks.com/w/file/fetch/127916259/MMAT_2018_criteria-manual_2018-08-01_ENG.pdf. [Accessed 10 June 2019].
[22] Braun, V. & Clarke, V. (2006). Using thematic analysis in psychology. *Qualitative Research in Psychology*. 3 (2): pp. 77-101.
[23] Wheeldon, J. & Åhlberg, M. K. (2012). Mapping mixed-methods research: theories, models, and measures. In Wheeldon, J., & Åhlberg, M. K. *Visualizing Social Science Research: Maps, Methods, & Meaning* (pp. 113-148). Thousand Oaks, CA: SAGE Publications, Inc.
[24] Swendeman, D., Farmer, S., Mindry, D., Lee, S. J. & Medich, M. (2016). HIV Care Providers' Attitudes regarding Mobile Phone Applications and Web-Based Dashboards to support Patient Self-Management and Care Coordination: Results from a Qualitative Feasibility Study. *Journal of HIV and AIDS*, 2 (4): pp. 10.
[25] Luque, A. E., Corales, R., Fowler, R. J., Dimarco, J., Van Keken, A., Winters, P., Keefer, M. C. & Fiscella, K. (2013). Bridging the Digital Divide in HIV Care: A Pilot Study of an iPod Personal Health Record. *Journal of the International Association of Providers of AIDS Care (JIAPAC),* 12 (2): pp. 117-121
[26] Odlum, M., Gordon, P., Camhi, E., Valdez, E. & Bakken, S. (2014). Exploring Factors Related to the Adoption and Acceptance of an Internet-based Electronic Personal Health Management Tool (EPHMT) in a Low Income, Special Needs Population of People Living with HIV and AIDS in New York City, *Studies in Health Technology and Informatics,* 201 (1): pp. 145-152.
[27] Nokes, K., Verkuilen, J., Hickey, D., James-Borga, J. & Shan, J. (2013). Developing a personal health record self-efficacy tool. *Applied Nursing Research,* 26 (1): pp. 32-9.
[28] Schnall, R., Bakken, S., Brown Iii, W., Carballo-Dieguez, A. & Iribarren, S. (2016). Usabilty Evaluation of a Prototype Mobile App for Health Management for Persons Living with HIV. *Studies in Health Technology and Informatics*, 225 (1): pp. 481–485.
[29] Stonbraker, S., Cho, H., Hermosi, G., Pichon, A. & Schnall, R. (2018). Usability Testing of a mHealth App to Support Self-Management of HIV-Associated Non-AIDS Related Symptoms. *Studies in Health Technology and Informatics*, 250 (1): pp. 106–110.
[30] Ramanathan, N., Swendeman, D., Comulada, W. S., Estrin, D. & Rotheram-Borus, M. J. (2013). Identifying preferences for mobile health applications for self-monitoring and self-



For peer review only - http://bmjopen.bmj.com/site/about/guidelines.xhtml

management: Focus group findings from HIV-positive persons and young mothers. *International Journal of Medical Informatics,* 82 (4): pp. 38-46.
[31] Schnall, R., Gordon, P., Camhi, E. & Bakken, S. (2011). Perceptions of factors influencing use of an electronic record for case management of persons living with HIV. *AIDS Care,* 23 (3): pp. 357-365.
[32] DeSilva, M. B., Gifford, A. L., Xu, K., Zhong, L., Feng, C., Brooks, M., Harrold, M., Yueying, H., Gill, C. J., Wubin, X., Vian, T., Haberer, J., Bangsberg, D. & Sabin, L. (2013). *AIDS Research and Treatment,* Article ID: 957862.
[33] Bussone, A. (2018). Reflection and personal health informatics for people living with HIV. PhD Thesis, City University of London.
[34] Marent, B., Henwood, F. & Darking, M. (2018). Development of an mHealth platform for HIV Care: Gathering User Perspectives Through Co-Design Workshops and Interviews. *JMIR mHealth and uHealth*, 6 (10): pp. e184.
[35] Robinson, L., Cotten, S., Ono, H., Quan-Haase, A., Mesch, G., Chen, W., Schulz, J., Hale, T. M. & Stern, M. (2015). Digital inequalities and why they matter. *Information, Communication & Society,* 18 (5): pp. 1-14.
[36] Piwek, L., Ellis, D. A., Andrews, S. & Joinson, A. (2016). The Rise of Consumer Health Wearables: Promises and Barriers. *PLoS Medicine,* 13 (2): pp. e1001953.
[37] Lupton, D. & Maslen, S. (2019). How Women Use Digital Technologies for Health: Qualitative Interview and Focus Group Study. *Journal of Medical Internet Research*, 21 (1): pp. e11481.
[38] Singh, A., Gibbs, J., Estcourt, C., Sonnenberg, P. & Blandford, A. (2017). DH '17: Proceedings of the 2017 International Conference on Digital Health July 2017 Pages 6–15. DOI: https://doi.org/10.1145/3079452.3079469.
[39] Fettig, J., Swaminathan, M., Murrill, C. S. & Kaplan, J. E. (2014). Global epidemiology of HIV. *Infectious Disease Clinics of North America*. 28 (3): pp. 323-337.
[40] McAuley, A. (2014). Digital health interventions: widening access or widening inequalities? *Public Health,* 128 (12): pp. 1118-1120.
[41] Jongbloed, K., Parmar, S., van der Kop, M., Spittal, P. M., & Lester, R. T. (2015). Recent Evidence for Emerging Digital Technologies to Support Global HIV Engagement in Care. *Current HIV/AIDS reports*, 12 (4): pp. 451–461.
[42] Beaunoyer, E., Dupéré, S. & Guitton, M. J. (2020). COVID-19 and digital inequalities: Reciprocal impacts and mitigation strategies. *Computers in Human Behaviour,* 111.
[43] Watts, G. (2020). COVID-19 and the digital divide in the UK. *The Lancet Digital Health,* 2 (8): E395-396.
[44] Liu, A., Coleman, K., Bojan, K., Serrano, P. A., Oyedele, T., Garcia, A., Enriquez-Bruce, E., Emmanuel, P., Jones, J., Sullivan, P., Hightow-Weidman, L., Buchbinder, S. & Scott, H. (2019). Developing a Mobile App (LYNX) to Support Linkage to HIV/Sexually Transmitted Infection Testing and Pre-Exposure Prophylaxis for Young Men Who Have Sex with Men: Protocol for a Randomized Controlled Trial. *JMIR research Protocols*, 8 (1): pp. e10659.
[45] McGraw, D., Belfort, R., Pfister, H. & Ingargiola, S. (2013). Going digital with patients: Managing potential liability risks of patient-generated electronic health information. *Journal of Participatory Medicine*. 18 (5): pp. e41. Available from: http://www.jopm.org/perspective/narratives/2013/12/18/going-digital-with-patients-managing-potential-liability-risks-of-patient-generated-electronic-health-information. [Accessed 20 July 2019].
[46] West, P., Van Kleek, M., Giordano, R., Weal, M. J. & Shadbolt, N. (2018). CHI '18: Proceedings of the 2018 CHI Conference on Human Factors in Computing SystemsApril 2018 Paper No.: 484 Pages 1–13, DOI: https://doi.org/10.1145/3173574.3174058



For peer review only - http://bmjopen.bmj.com/site/about/guidelines.xhtml

[47] Huba, N. & Zhang, Y. (2012). Designing patient-centred personal health records (PHRs): Health care professionals' perspective on patient-generated data. *Journal of Medical Systems,* 36 (6): pp. 3893-3905.
[48] Mehraeen, E., Safdari, R., Mohammadzadeh, N., Seyedalinaghi, S., Forootan, S. & Mohran, M. (2018). Mobile-Based Applications and Functionalities for Self-Management of People Living with HIV. *Health Informatics Meets eHealth,* 248: pp. 172-179.
[49] Torous, J., Roberts, L. W. (2017). Needed Innovation in Digital Health and Smartphone Applications for Mental Health: Transparency and Trust. *JAMA Psychiatry*, 74 (5): pp. 437-438.
[50] Huckvale, K., Prieto, J. T., Tilney, M., Benghozi, P. J. & Car, J. (2015). Unaddressed privacy risks in accredited health and wellness apps: a cross-sectional systematic assessment. *BMC Medicine*, 13: pp. 214.
[51] Gibbs, J., Gkatzidou, V., Tickle, L., Manning, S. R., Tilakkumar, T., Hone, K., Ashcroft, R. E., Sonnenberg, P., Sadiq, S. T. & Estcourt, C. S. (2017). 'Can you recommend any good STI apps?' A review of content, accuracy and comprehensiveness of current mobile medical applications for STIs and related genital infections. *Sexually Transmitted Infections,* 93 (4): pp. 234-235.
[52] Effy, V., Tobias, H., Afua, A. & Alessandro, B. (2018). Digital health: meeting the ethical and policy challenges. *Swiss Medical Weekly,* 148: pp. w14571.
[53] Beauclair, R., Meng, F., Deprez, N., Temmerman, M., Welte, A., Hens, N. & Delva, W. (2013). Evaluating audio computer assisted self-interviews in urban south African communities: Evidence for good suitability and reduced social desirability bias of a cross-sectional survey on sexual behaviour. *BMC Medical Research Methodology,* 13 (1): pp.11.
[54] Powell, A. C., Landman, A. B. & Bates, D. W. In search of a few good apps. (2014). *JAMA,* 311 (18): pp. 1851-1852.
[55] Lordon, R. J., Mikles, S. P., Kneale, L., Evans, H. L., Munson, S. A., Backonja, U. & Lober, W. B. (2020). How patient-generated health data and patient-reported outcomes affect patient-clinician relationships: A systematic review. *Health Informatics Journal,* 1460458220928184
[56] Greenhalgh, T. & Peacock, R. (2005). Effectiveness and efficiency of search methods in systematic reviews of complex evidence: audit of primary sources. *BMJ,* 331: pp. 1064
[57] Sinha, C. & Schryer-Roy, A. M. (2018). Digital health, gender and health equity: invisible imperatives. (2018). *Journal of Public Health (Oxford)*. 40 (2): pp. 21-25.
[58] Jiang, H., Zhou, Y. & Tang, W. (2020). Maintining HIV care during the COVID-19 pandemic. *The Lancet HIV,* 7 (5): pp. e308-e309.
[59] Kowalska, J. D., Skrzat-Klapaczyńska, A., Bursa, D., Balayan, T., Begovac, J., Chkhartishvili, N., Gokengin, D., Harxhi, A., Jilich, D., Jevtovic, D., Kase, K., Lakatos, B., Matulionyte, R., Mulabdic, V., Nagit, A., Papadopoulos, A., Stefanovic, M., Vassilenko, A., Vasylyev, M., Yancheva, N., Yurin, O. & Horban, A., for the ECEE Network Group. (2020). HIV care in times of the COVID-19 crisis - Where are we now in Central and Eastern Europe? *International Journal of Infectious Diseases,* 96: pp. 311–314.
[60] Watson, A. R., Wah, R. & Thamman, R. (2020). The Value of Remote Monitoring for the COVID-19 Pandemic. *Telemedicine and e-Health. Ahead of print.*
[61] Peek, N., Sujan, M. & Scott, P. (2020). Digital health and care in pandemic times: impact of COVID-19. *BMJ Health & Care Informatics,* 27: e100166.
[62] Charani, E., Castro-Sánchez, E., Moore, L. S. & Holmes, A. (2014). Do smartphone applications in healthcare require a governance and legal framework? It depends on the application! *BMC Medicine,* 12 (1): pp. 29.



For peer review only - http://bmjopen.bmj.com/site/about/guidelines.xhtml

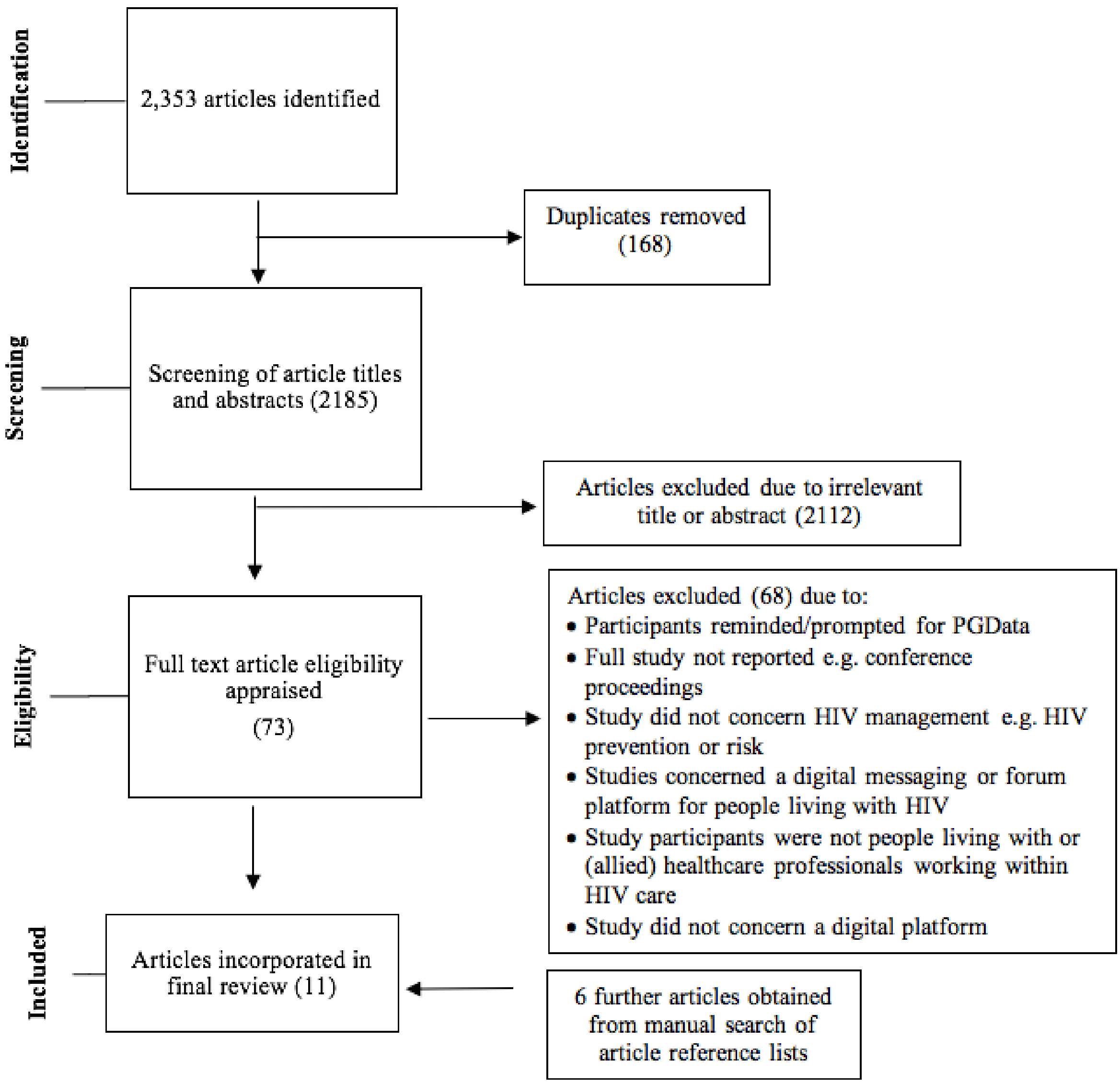


**Figure 1.** ***PRISMA flow diagram.***

## Preferred Reporting Items for Systematic reviews and Meta-Analyses extension for Scoping Reviews (PRISMA-ScR) Checklist

| SECTION | ITEM | PRISMA-ScR CHECKLIST ITEM | REPORTED ON PAGE # |
|---|---|---|---|
| **TITLE** | | | |
| Title | 1 | Identify the report as a scoping review. | |
| **ABSTRACT** | | | |
| Structured summary | 2 | Provide a structured summary that includes (as applicable): background, objectives, eligibility criteria, sources of evidence, charting methods, results, and conclusions that relate to the review questions and objectives. | |
| **INTRODUCTION** | | | |
| Rationale | 3 | Describe the rationale for the review in the context of what is already known. Explain why the review questions/objectives lend themselves to a scoping review approach. | |
| Objectives | 4 | Provide an explicit statement of the questions and objectives being addressed with reference to their key elements (e.g., population or participants, concepts, and context) or other relevant key elements used to conceptualize the review questions and/or objectives. | |
| **METHODS** | | | |
| Protocol and registration | 5 | Indicate whether a review protocol exists; state if and where it can be accessed (e.g., a Web address); and if available, provide registration information, including the registration number. | |
| Eligibility criteria | 6 | Specify characteristics of the sources of evidence used as eligibility criteria (e.g., years considered, language, and publication status), and provide a rationale. | |
| Information sources* | 7 | Describe all information sources in the search (e.g., databases with dates of coverage and contact with authors to identify additional sources), as well as the date the most recent search was executed. | |
| Search | 8 | Present the full electronic search strategy for at least 1 database, including any limits used, such that it could be repeated. | |
| Selection of sources of evidence† | 9 | State the process for selecting sources of evidence (i.e., screening and eligibility) included in the scoping review. | |
| Data charting process‡ | 10 | Describe the methods of charting data from the included sources of evidence (e.g., calibrated forms or forms that have been tested by the team before their use, and whether data charting was done independently or in duplicate) and any processes for obtaining and confirming data from investigators. | |
| Data items | 11 | List and define all variables for which data were sought and any assumptions and simplifications made. | |
| Critical appraisal of individual sources of evidence§ | 12 | If done, provide a rationale for conducting a critical appraisal of included sources of evidence; describe the methods used and how this information was used in any data synthesis (if appropriate). | |
| Synthesis of results | 13 | Describe the methods of handling and summarizing the data that were charted. | |

St. Michael's
Inspired Care.
Inspiring Science.

For peer review only - http://bmjopen.bmj.com/site/about/guidelines.xhtml

| SECTION | ITEM | PRISMA-ScR CHECKLIST ITEM | REPORTED ON PAGE # |
|---|---|---|---|
| **RESULTS** | | | |
| Selection of sources of evidence | 14 | Give numbers of sources of evidence screened, assessed for eligibility, and included in the review, with reasons for exclusions at each stage, ideally using a flow diagram. | |
| Characteristics of sources of evidence | 15 | For each source of evidence, present characteristics for which data were charted and provide the citations. | |
| Critical appraisal within sources of evidence | 16 | If done, present data on critical appraisal of included sources of evidence (see item 12). | |
| Results of individual sources of evidence | 17 | For each included source of evidence, present the relevant data that were charted that relate to the review questions and objectives. | |
| Synthesis of results | 18 | Summarize and/or present the charting results as they relate to the review questions and objectives. | |
| **DISCUSSION** | | | |
| Summary of evidence | 19 | Summarize the main results (including an overview of concepts, themes, and types of evidence available), link to the review questions and objectives, and consider the relevance to key groups. | |
| Limitations | 20 | Discuss the limitations of the scoping review process. | |
| Conclusions | 21 | Provide a general interpretation of the results with respect to the review questions and objectives, as well as potential implications and/or next steps. | |
| **FUNDING** | | | |
| Funding | 22 | Describe sources of funding for the included sources of evidence, as well as sources of funding for the scoping review. Describe the role of the funders of the scoping review. | |

JBI = Joanna Briggs Institute; PRISMA-ScR = Preferred Reporting Items for Systematic reviews and Meta-Analyses extension for Scoping Reviews.

* Where *sources of evidence* (see second footnote) are compiled from, such as bibliographic databases, social media platforms, and Web sites.

† A more inclusive/heterogeneous term used to account for the different types of evidence or data sources (e.g., quantitative and/or qualitative research, expert opinion, and policy documents) that may be eligible in a scoping review as opposed to only studies. This is not to be confused with *information sources* (see first footnote).

‡ The frameworks by Arksey and O'Malley (6) and Levac and colleagues (7) and the JBI guidance (4, 5) refer to the process of data extraction in a scoping review as data charting.

§ The process of systematically examining research evidence to assess its validity, results, and relevance before using it to inform a decision. This term is used for items 12 and 19 instead of "risk of bias" (which is more applicable to systematic reviews of interventions) to include and acknowledge the various sources of evidence that may be used in a scoping review (e.g., quantitative and/or qualitative research, expert opinion, and policy document).

*From:* Tricco AC, Lillie E, Zarin W, O'Brien KK, Colquhoun H, Levac D, et al. PRISMA Extension for Scoping Reviews (PRISMAScR): Checklist and Explanation. Ann Intern Med. 2018;169:467–473. doi: 10.7326/M18-0850.


For peer review only - http://bmjopen.bmj.com/site/about/guidelines.xhtml

**Appendix B-** Search terms used within the literature search strategy.

Keywords for each subject heading of ‘HIV’ and ‘patient-generated health data’ respectively were combined using the search command ‘OR’. Articles yielded within each of these searches were combined using the search command ‘AND’ to produce the final article results.

## Keywords used

***Subject heading: HIV***

HIV OR human immunodeficiency virus OR HIV/AIDS OR HIV-infected OR HIV-associated OR HIV-positive OR HIV positive OR HIV+ OR people living with HIV OR persons living with HIV OR HIV patient* OR HIV infection

***Subject heading: Patient-generated health data***

Patient generated data OR patient-generated data OR patient generated health data OR patient-generated health data OR patient generated health information OR patient-generated health information OR personal health record* OR patient portal* OR personal data sharing OR patient collected data OR patient-collected data OR personal health informati* OR personal health data OR electronic health record* OR electronic personal health record* OR electronic health data OR self-recorded health data OR self-recorded data OR self-generated data or self-reported data OR patient collated data OR patient-collected data OR patient-captured data OR patient-controlled data OR patient-reported data OR electronic record.

An example complete database search strategy, for Medline, is shown below:

For peer review only

| | # ▲ | Searches | Results | Type | Actions | Annotations |
|---|---|---|---|---|---|---|
| | 1 | exp HIV Infections/ or HIV/ or exp HIV Long-Term Survivors/ | 277245 | Advanced | Display Results More ▾ | |
| | 2 | (HIV or human immunodeficiency virus).mp. or HIV/aids or HIV-infected.mp. or HIV-associated.mp. or HIV-positive.mp. or HIV positive.mp. or HIV+.mp. or people living with HIV.mp. or persons living with HIV.mp. or HIV patient*.mp. or HIV infection.mp. [mp=title, abstract, original title, name of substance word, subject heading word, floating sub-heading word, keyword heading word, organism supplementary concept word, protocol supplementary concept word, rare disease supplementary concept word, unique identifier, synonyms] | 351883 | Advanced | Display Results More ▾ | |
| | 3 | exp health records, personal/ or exp patient generated health data/ or exp patient portals/ | 1636 | Advanced | Display Results More ▾ | |
| | 4 | (Patient generated data or patient-generated data or patient generated health data or patient-generated health data or patient generated health information or patient-generated health information or personal health record* or patient portal* or personal data sharing or patient collected data or patient-collected data or personal health informati* or personal health data or electronic health record* or electronic personal health record* or electronic health data or self-recorded health data or self-recorded data or self-generated data or self-reported data or patient collated data or patient-collected data or patient-captured data or patient-controlled data or patient-reported data or electronic record).mp. [mp=title, abstract, original title, name of substance word, subject heading word, floating sub-heading word, keyword heading word, organism supplementary concept word, protocol supplementary concept word, rare disease supplementary concept word, unique identifier, synonyms] | 28979 | Advanced | Display Results More ▾ | |
| | 5 | 1 or 2 | 391186 | Advanced | Display Results More ▾ | |
| | 6 | 3 or 4 | 29523 | Advanced | Display Results More ▾ | |
| | 7 | 5 and 6 | 489 | Advanced | Display Results More ▾ | |

## Appendix C- Data extraction table of included articles

| **Studies concerning a hypothetical digital platform for PGData collection** | | | | | | | |
|---|---|---|---|---|---|---|---|
| **Authors (date)** | **Location** | **Participants** | **Study design** | **Length of study** | **Study aims and summary** | **Digital platform summary** | **Measures** |
| Bussone (2018, Study One of three) | England, UK | N = 16 people living with HIV<br><br>Age: range 18-57 years, M = 39.87<br><br>Gender: 8 male, 8 female<br><br>Ethnicity: 1 White British, 1 White other, White/Black African, 8 Black African, 2 Latin, 1 Asian Pakistani, 1 White/Black/Caribbean, 1 Mixed other<br><br>Orientation: 5 homosexual, 9 heterosexual, 1 bisexual, 1 asexual | Qualitative, individual semi-structured interviews of approximately 30 minutes | N/A | • **Aim**: to understand how people living with HIV monitor their personal health data<br>**Topics addressed:** participants' current self-management behaviours and issues surrounding current, previous or desired means of tracking personal health information | N/A as no specific digital platform used | Verbal feedback from participants regarding self-tracking of their personal health information |

For peer review only

| | | | | | | | |
|---|---|---|---|---|---|---|---|
| Marent, Henwood & Darking (2018) | Brighton (UK), Lisbon (Portugal), Barcelona (Spain), Antwerp (Belgium) and Zagreb (Croatia) | N = 160<br>• 97 people living with HIV<br>• 63 HCPs working in HIV care<br>-40 doctors<br>-10 nurses<br>-4 psychologists<br>-4 pharmacists<br>-2 social workers<br>-2 nutritionists<br>-1 sexologist<br><br>Age (of people living with HIV): range = 23-78 years<br><br>Gender (people living with HIV): 78 male<br><br>Ethnicity: authors report that people living with HIV had 14 different nationalities, with 13 identifying as belonging to a migrant community<br><br>Orientation (people living with HIV): 65 gay or lesbian, 26 heterosexual, 3 bisexual, 3 other. | Qualitative, 14 recorded workshops and 22 semi-structured interviews | January-June 2016 | • **Aim**: to determine perceptions of, and advantages and concerns surrounding, the development of a mobile app for HIV management<br>• **Topics addressed:** perceived challenges and barriers to use of a mobile app for HIV management, current mobile app usage and perceived useful features of a mobile app for HIV management | A hypothetical mobile app was discussed. | Verbal feedback from participants regarding factors related to a hypothetical mobile app for HIV management |

For peer review only

| | | | | | | | |
|---|---|---|---|---|---|---|---|
| Nokes et al. (2013) | New York, USA | N = 100 people living with HIV/AIDS<br><br>Age: M= 48.5<br><br>Gender: 94 male<br><br>Orientation: unreported<br><br>Ethnicity: 7 Asian/Pacific Islander, 51 African American/Black, 34 Hispanic/Latino, 1 Native American, 4 White/Anglo, 3 other<br><br>Education: 21 <11th grade, 45 high school/general education development, 25 2-years college, 9 4+ years of college | Quantitative | N/A | • **Aim:** to assess self-efficacy of people living with HIV regarding use of a hypothetical personal health record<br>• Participants received an explanation of a personal health record and completed questionnaires measuring their self-efficacy in relation to digital and paper-based personal health records, condom use and chronic disease management | N/A as no specific digital platform used | • Self-efficacy for creation and use of a paper-based personal health record<br>• Self-efficacy for creation and use of a digital personal health record<br>• Self-efficacy regarding medication adherence (HIV-Treatment Adherence Self-Efficacy Scale)<br>• Self-efficacy for chronic disease management scale<br>Self-efficacy for condom usage scale |

For peer review only

| | | | | | | | |
|---|---|---|---|---|---|---|---|
| Ramanathan et al. (2013) | Los Angeles, USA | N = 29 people living with HIV<br><br>Age: range= 30-60 years<br><br>Gender: 75% male<br><br>-Ethnicity: reported approximately one third of participants in each ethnic group of African American, Latino and White<br><br>Orientation: 55% men who have sex with men, 10% transgender | Qualitative, focus group discussions | N/A | • **Aim:** to assess feature preferences for a mobile app for HIV self-management<br>• **Topics addressed:** privacy, goal-setting, data capturing methods, feedback regarding app-user behaviours and the role of reminders | A hypothetical mobile app was discussed. | • Verbal feedback regarding key issues pertaining to development of a mobile app for HIV self-management |

For peer review only

| | | | | | | | |
|---|---|---|---|---|---|---|---|
| Swendeman et al. (2016) | Los Angeles, USA | N = 12 HCPs from a Los Angeles County HIV clinic<br>• 3 doctors<br>• 1 nurse practitioner<br>• 2 nurse case managers<br>• 2 psychosocial case managers<br>• 1 psychosocial case manager<br>• 1 psychotherapist<br><br>Age: unreported<br><br>Gender: unreported<br><br>Ethnicity: unreported<br><br>Orientation: unreported<br><br>Education: unreported | Qualitative, individual 45-60 minute semi-structured interviews | N/A | • **Aim:** to assess HCP attitudes regarding a hypothetical mobile app (for people living with HIV) and online dashboards (for HCPs) for HIV management<br>• **Topics addressed:** usability, acceptability and barriers to digital platform use | A hypothetical mobile app and online dashboard were discussed. | • Verbal feedback from participants regarding the use of hypothetical web-based dashboards for HCPs and hypothetical mobile apps for people living with HIV, both to facilitate self-management in patients |

| | | | | | | | |
|---|---|---|---|---|---|---|---|
| **Studies concerning a novel digital platform for PGData collection** | | | | | | | |
| Schnall et al. (2011) | New York, USA | N = 37 Case Managers working with people living with HIV<br><br>Age: unreported<br><br>Gender: unreported<br><br>Ethnicity: unreported<br><br>Orientation: unreported | Qualitative, one-hour focus group discussions | March-December 2008 | • **Aim:** to determine factors influencing usage and acceptability of online "SelectHealth Continuity of Care Document" for people living with HIV, before the platform's release<br>• **Topics addressed:** facilitators and barriers to use of the continuity of care record | The "SelectHealth Continuity of Care Document" includes key health information including latest medical test results, pharmaceutical record, visits to healthcare facilities and patient contact information | • Verbal feedback regarding perceived facilitators and barriers to use of the "SelectHealth Continuity of Care Document" |
| Schnall et al. (2016) | New York, USA | N = 15<br>• 10 people living with HIV<br>• 5 informaticians<br><br>Age: unreported<br><br>Gender (people living with HIV): 6 male<br><br>Ethnicity: unreported<br><br>Orientation: unreported | Mixed: quantitative, qualitative | [illegible] | • **Aim:** to test a prototype mobile app designed to facilitate HIV management in people living with HIV Participants received a description of a mobile app prototype for HIV management<br>• Informaticians tested the model for 45-90 minutes and completed a Heuristic Evaluation Checklist<br>• people living with HIV evaluated prototype app screenings and completed the Post Study System Usability Questionnaire | No description of mobile app prototype provided | • For informaticians: score on Heuristic Evaluation Checklist<br>• For people living with HIV: score on Post Study System Usability Questionnaire as a measurement of the app's usability |

For peer review only

| | | | | | | | |
|---|---|---|---|---|---|---|---|
| Stonbraker et al. (2018) | New York, USA | N= 25<br>• 20 people living with HIV who possessed at least one HIV-associated non-AIDS condition<br>• 5 informaticians<br><br>Age: M= 54.4 years<br><br>Gender: 40% male<br><br>Ethnicity: only reported 85% African American<br><br>Orientation: unreported<br><br>Education: 50% <high school, 35% high school, 15% more than high school | Mixed: quantitative, qualitative | N/A | • **Aim:** to determine usability of novel mobile app *VIP-HANA* (Video Information Provider for HIV-associated non-AIDS conditions) designed to aid people living with HIV with management of HIV/non-HIV-related symptoms, and to determine where improvements to the app were needed<br>• After receiving descriptions of the app participants were assigned tasks on a beta version of the app and had to describe aloud their thoughts and actions whilst their activities were tracked<br>• people living with HIV completed one questionnaire to assess their health literacy level, and two questionnaires relating to the app's usability | • Within the app individuals must indicate which HIV-associated non-AIDS conditions they possess out of nine options<br>• Individuals can then categorise associated symptoms of the condition(s) out of 28 options, according to their severity<br>Upon severity categorisation, the app-user receives tailored strategies to facilitate self-management behaviours | • For informaticians: assigned mobile app task activity and score on Heuristic Evaluation Checklist<br>• For people living with HIV: assigned mobile app task activity, score on the Newest Vital Sign assessment (to determine health literacy level) and scores on two questionnaires relating to app usability (Health Information Technology Usability Evaluation Scale and Post Study System Usability Questionnaire) |

| **Studies concerning an existing, established digital platform for PGData collection** | | | | | | | |
|---|---|---|---|---|---|---|---|
| **Authors (date)** | **Location** | **Participants** | **Study design** | **Length of study** | • **Study aims and summary** | • **Digital platform summary** | • **Measures** |
| DeSilva et al. (2013) | Nanning, China | N= 10 people living with HIV who were injection drug users<br><br>Age: M = 32.7 years<br><br>Gender: 7 male<br><br>Orientation: unreported<br><br>Ethnicity: unreported<br><br>Education: 9 participants <middle school | Qualitative, individual interviews before and after using the device | One month | • **Aim:** to determine the feasibility and acceptability of using a *Wisepill* device in injecting drug users who were HIV positive<br>• Participants used the *Wisepill* device to monitor their antiretroviral medication adherence for one month<br>• **Topics addressed:** acceptability, feasibility and usability of the device | • The *Wisepill* container stores medication<br>• Upon opening, a wireless signal is immediately transmitted to a server, the data of which can be obtained by HCPs<br>• Opening the *Wisepill* container provides a proxy measurement of medication adherence | • Verbal feedback from participants regarding feasibility, acceptability and usability of the *Wisepill* device to track medication adherence<br>• Self-reported adherence to antiretroviral medication after one month of using the *Wisepill* device |

For peer review only

| | | | | | | | |
|---|---|---|---|---|---|---|---|
| Luque et al. (2013) | Rochester, New York, USA | N = 29 people living with HIV<br><br>Age: M= 48<br><br>Gender: 55% male, 41% female, 3% transgender<br><br>Ethnicity: 41% African American, 21% Latino, 28% white, 10% other<br><br>Orientation: unreported<br><br>Education level: 31% <high school, 28 % high school or equivalent, 41% >high school<br><br>Annual household income: 83% <$20,000, 17% >$20,000<br><br>Use of electronic technology: 18% never used a computer, 21% never used the internet, 36% never used email | Quantitative | 6 weeks | • **Aim:** to determine acceptability and usability of a personal health record on an iPod touch device, and to observe the effect of using a digital personal health record upon HIV treatment self-efficacy<br>• Participants underwent six 90-minute training sessions and were given 'homework' tasks to rehearse between training sessions | • The *MyMedical* app, (downloaded from iTunes Store) was trialled as a personal health record on an iPod touch device<br>• No more information regarding the app was provided within the study | • HIV-Treatment Adherence Self-Efficacy Scale (baseline and one month post-training)<br>• Post-training questionnaire score for iPod device usability<br>• Post-training questionnaire score for personal health record usability<br>• Attitudes Towards Computers Scale |

For peer review only

| | | | | | | | |
|---|---|---|---|---|---|---|---|
| Odlum et al. (2014) | New York, USA | N= 57 people living with HIV<br>• Quantitative: 41 people living with HIV<br>-M age= 47 years, range 24-63 years<br><br>-Gender: 27 male<br><br>-Ethnicity: 27 Black/African American, 11 other/mixed race<br><br>-Orientation: unreported<br><br>-Education: 25 <high school diploma, 4 associate degrees, 2 Bachelor's degrees<br><br>• Qualitative: 15 people living with HIV,<br>8 users of the *MyHealthProfile* app<br><br>-Gender: 2 male<br><br>-Ethnicity: 7 Black African American, 1 Latino<br><br>-Orientation: unreported | Mixed: quantitative (System Usefulness Survey), qualitative (focus groups of 1 hour) | N/A | • **Aim:** to determine usability of the *MyHealthProfile* app and to determine where improvements could be made to inform a new version of the app named *MyHealthProfile-plus*<br>• Participants completed 2 surveys relating to the usefulness and content of the pre-established *My Health Profile* app<br>• Participants completed 60 minute focus groups regarding the development of new *My Health Profile-plus* app | • The *MyHealthProfile* app acts as a continuity of care document for people living with HIV to track their personal health record via an internet connection | • System Usefulness Survey<br>• Ease of Use Scale<br>• App usefulness score<br>• App components score<br>• Verbal feedback regarding perceived app needs for novel app *My Health Profile-plus* |

For peer review only

| | | | | | | | |
|---|---|---|---|---|---|---|---|
| | | 7 non-users of the *MyHealthProfile* app<br>-Gender: 5 male<br>-Ethnicity: 6 Black African American, 1 Caucasian<br><br>-Orientation: unreported | | | | | |

For peer review only

# Appendix D- Results from the Mixed Methods Appraisal Tool assessment

## Bussone (2018)

| Study design | Methodological quality criteria | Responses: Yes | No | Can't tell |
|---|---|---|---|---|
| **Screening questions (all studies)** | S1. Are there clear research questions? | X | | |
| | S2. Do the collected data allow to address the research questions? | X | | |
| **Qualitative** | 1.1. Is the qualitative approach appropriate to answer the research question? | X | | |
| | 1.2. Are the qualitative data collection methods adequate to address the research question? | X | | |
| | 1.3. Are the findings adequately derived from the data? | X | | |
| | 1.4. Is the interpretation of results sufficiently substantiated by data? | X | | |
| | 1.5. Is there coherence between qualitative data sources, collection, analysis and interpretation? | X | | |

**DeSilva et al. (2013)**

| | | Responses | | |
|---|---|---|---|---|
| **Study design** | **Methodological quality criteria** | **Yes** | **No** | **Can't tell** |
| **Screening questions (all studies)** | S1. Are there clear research questions? | X | | |
| | S2. Do the collected data allow to address the research questions? | X | | |
| **Qualitative** | 1.1. Is the qualitative approach appropriate to answer the research question? | X | | |
| | 1.2. Are the qualitative data collection methods adequate to address the research question? | X | | |
| | 1.3. Are the findings adequately derived from the data? | | | X |
| | 1.4. Is the interpretation of results sufficiently substantiated by data? | | | X |
| | 1.5. Is there coherence between qualitative data sources, collection, analysis and interpretation? | | | X |

**Luque et al. (2013)**

| Study design | Methodological quality criteria | Responses | | |
|---|---|---|---|---|
| | | Yes | No | Can't tell |
| **Screening questions (all studies)** | S1. Are there clear research questions? | X | | |
| | S2. Do the collected data allow to address the research questions? | X | | |
| **Quantitative descriptive** | 4.1. Is the sampling strategy relevant to address the research question? | X | | |
| | 4.2. Is the sample representative of the target population? | | X | |
| | 4.3. Are the measurements appropriate? | X | | |
| | 4.4. Is the risk of nonresponse bias low? | X | | |
| | 4.5. Is the statistical analysis appropriate to answer the research question? | X | | |

For peer review only

**Marent, Henwood & Darking (2018)**

| | | Responses | | |
|---|---|---|---|---|
| **Study design** | **Methodological quality criteria** | **Yes** | **No** | **Can't tell** |
| **Screening questions (all studies)** | S1. Are there clear research questions? | X | | |
| | S2. Do the collected data allow to address the research questions? | X | | |
| **Qualitative** | 1.1. Is the qualitative approach appropriate to answer the research question? | X | | |
| | 1.2. Are the qualitative data collection methods adequate to address the research question? | X | | |
| | 1.3. Are the findings adequately derived from the data? | X | | |
| | 1.4. Is the interpretation of results sufficiently substantiated by data? | X | | |
| | 1.5. Is there coherence between qualitative data sources, collection, analysis and interpretation? | X | | |

For peer review only

**Nokes et al. (2013)**

| Study design | Methodological quality criteria | Responses | | |
|---|---|---|---|---|
| | | **Yes** | **No** | **Can't tell** |
| **Screening questions (all studies)** | S1. Are there clear research questions? | X | | |
| | S2. Do the collected data allow to address the research questions? | X | | |
| **Quantitative descriptive** | 4.1. Is the sampling strategy relevant to address the research question? | X | | |
| | 4.2. Is the sample representative of the target population? | X | | |
| | 4.3. Are the measurements appropriate? | X | | |
| | 4.4. Is the risk of nonresponse bias low? | X | | |
| | 4.5. Is the statistical analysis appropriate to answer the research question? | X | | |

For peer review only

**<u>Odlum et al. (2014)</u>**

| | | Responses | | |
|---|---|---|---|---|
| **Study design** | **Methodological quality criteria** | **Yes** | **No** | **Can't tell** |
| **Screening questions (all studies)** | S1. Are there clear research questions? | X | | |
| | S2. Do the collected data allow to address the research questions? | X | | |
| **Qualitative** | 1.1. Is the qualitative approach appropriate to answer the research question? | X | | |
| | 1.2. Are the qualitative data collection methods adequate to address the research question? | X | | |
| | 1.3. Are the findings adequately derived from the data? | X | | |
| | 1.4. Is the interpretation of results sufficiently substantiated by data? | X | | |
| | 1.5. Is there coherence between qualitative data sources, collection, analysis and interpretation? | X | | |
| **Quantitative descriptive** | 4.1. Is the sampling strategy relevant to address the research question? | | | X |

For peer review only

| | | | | |
|---|---|---|---|---|
| | 4.2. Is the sample representative of the target population? | | X | |
| | 4.3. Are the measurements appropriate? | | | X |
| | 4.4. Is the risk of nonresponse bias low? | | | X |
| | 4.5. Is the statistical analysis appropriate to answer the research question? | | | X |
| **Mixed methods** | 5.1. Is there an adequate rationale for using a mixed methods design to address the research question? | | X | |
| | 5.2. Are the different components of the study effectively integrated to answer the research question? | | X | |
| | 5.3. Are the outputs of the integration of qualitative and quantitative components adequately interpreted? | | X | |
| | 5.4. Are divergences and inconsistencies between quantitative and qualitative results adequately addressed? | | X | |
| | 5.5. Do the different components of the study adhere to the quality criteria of each tradition of the methods involved? | | | X |

For peer review only

**Ramanathan et al. (2013)**

| | | Responses | | |
|---|---|---|---|---|
| **Study design** | **Methodological quality criteria** | **Yes** | **No** | **Can't tell** |
| **Screening questions (all studies)** | S1. Are there clear research questions? | X | | |
| | S2. Do the collected data allow to address the research questions? | X | | |
| **Qualitative** | 1.1. Is the qualitative approach appropriate to answer the research question? | X | | |
| | 1.2. Are the qualitative data collection methods adequate to address the research question? | X | | |
| | 1.3. Are the findings adequately derived from the data? | X | | |
| | 1.4. Is the interpretation of results sufficiently substantiated by data? | X | | |
| | 1.5. Is there coherence between qualitative data sources, collection, analysis and interpretation? | X | | |

For peer review only

**Swendeman et al. (2016)**

| Study design | Methodological quality criteria | Responses | | |
|---|---|---|---|---|
| | | Yes | No | Can't tell |
| **Screening questions (all studies)** | S1. Are there clear research questions? | X | | |
| | S2. Do the collected data allow to address the research questions? | X | | |
| **Qualitative** | 1.1. Is the qualitative approach appropriate to answer the research question? | X | | |
| | 1.2. Are the qualitative data collection methods adequate to address the research question? | X | | |
| | 1.3. Are the findings adequately derived from the data? | X | | |
| | 1.4. Is the interpretation of results sufficiently substantiated by data? | X | | |
| | 1.5. Is there coherence between qualitative data sources, collection, analysis and interpretation? | X | | |

For peer review only

**Schnall et al. (2011)**

| | | Responses | | |
|---|---|---|---|---|
| **Study design** | **Methodological quality criteria** | **Yes** | **No** | **Can't tell** |
| **Screening questions (all studies)** | S1. Are there clear research questions? | X | | |
| | S2. Do the collected data allow to address the research questions? | X | | |
| **Qualitative** | 1.1. Is the qualitative approach appropriate to answer the research question? | X | | |
| | 1.2. Are the qualitative data collection methods adequate to address the research question? | X | | |
| | 1.3. Are the findings adequately derived from the data? | X | | |
| | 1.4. Is the interpretation of results sufficiently substantiated by data? | X | | |
| | 1.5. Is there coherence between qualitative data sources, collection, analysis and interpretation? | X | | |

For peer review only

Schnall et al. (2016)

| | | Responses | | |
|---|---|---|---|---|
| **Study design** | **Methodological quality criteria** | **Yes** | **No** | **Can't tell** |
| **Screening questions (all studies)** | S1. Are there clear research questions? | X | | |
| | S2. Do the collected data allow to address the research questions? | | X | |
| **Qualitative** | 1.1. Is the qualitative approach appropriate to answer the research question? | | | X |
| | 1.2. Are the qualitative data collection methods adequate to address the research question? | | | X |
| | 1.3. Are the findings adequately derived from the data? | | | X |
| | 1.4. Is the interpretation of results sufficiently substantiated by data? | | | X |
| | 1.5. Is there coherence between qualitative data sources, collection, analysis and interpretation? | | | X |
| **Quantitative descriptive** | 4.1. Is the sampling strategy relevant to address the research question? | | X | |

For peer review only

| | | | | |
|---|---|---|---|---|
| | 4.2. Is the sample representative of the target population? | | X | |
| | 4.3. Are the measurements appropriate? | | | X |
| | 4.4. Is the risk of nonresponse bias low? | | | X |
| | 4.5. Is the statistical analysis appropriate to answer the research question? | | | N/A |
| **Mixed methods** | 5.1. Is there an adequate rationale for using a mixed methods design to address the research question? | | X | |
| | 5.2. Are the different components of the study effectively integrated to answer the research question? | | X | |
| | 5.3. Are the outputs of the integration of qualitative and quantitative components adequately interpreted? | | X | |
| | 5.4. Are divergences and inconsistencies between quantitative and qualitative results adequately addressed? | | X | |
| | 5.5. Do the different components of the study adhere to the quality criteria of each tradition of the methods involved? | | X | |

For peer review only

**Stonbraker et al. (2018)**

| | | Responses | | |
|---|---|---|---|---|
| **Study design** | **Methodological quality criteria** | **Yes** | **No** | **Can't tell** |
| **Screening questions (all studies)** | S1. Are there clear research questions? | X | | |
| | S2. Do the collected data allow to address the research questions? | X | | |
| **Qualitative** | 1.1. Is the qualitative approach appropriate to answer the research question? | | | X |
| | 1.2. Are the qualitative data collection methods adequate to address the research question? | | | X |
| | 1.3. Are the findings adequately derived from the data? | X | | |
| | 1.4. Is the interpretation of results sufficiently substantiated by data? | X | | |
| | 1.5. Is there coherence between qualitative data sources, collection, analysis and interpretation? | X | | |
| **Quantitative descriptive** | 4.1. Is the sampling strategy relevant to address the research question? | | | X |

For peer review only

| | | | | |
|---|---|---|---|---|
| | 4.2. Is the sample representative of the target population? | | X | |
| | 4.3. Are the measurements appropriate? | | | X |
| | 4.4. Is the risk of nonresponse bias low? | | | X |
| | 4.5. Is the statistical analysis appropriate to answer the research question? | | | X |
| **Mixed methods** | 5.1. Is there an adequate rationale for using a mixed methods design to address the research question? | | X | |
| | 5.2. Are the different components of the study effectively integrated to answer the research question? | | X | |
| | 5.3. Are the outputs of the integration of qualitative and quantitative components adequately interpreted? | | X | |
| | 5.4. Are divergences and inconsistencies between quantitative and qualitative results adequately addressed? | | X | |
| | 5.5. Do the different components of the study adhere to the quality criteria of each tradition of the methods involved? | | X | |

For peer review only

BMJ Open

# Patient-generated data in the management of HIV: a scoping review

| | |
|---|---|
| Journal: | *BMJ Open* |
| Manuscript ID | bmjopen-2020-046393.R2 |
| Article Type: | Original research |
| Date Submitted by the Author: | 15-Apr-2021 |
| Complete List of Authors: | Hewitt, Clara; University College London, Institute for Global Health<br>Lloyd, Karen; University College London, Centre for Population Research in Sexual Health and HIV, Institute for Global Health<br>Tariq, Shema; University College London, Institute for Global Health; University College London<br>Durrant, Abigail; Newcastle University, Open Lab, School of Computing<br>Claisse, Caroline; Newcastle University, Open Lab, School of Computing<br>Kasadha, Bakita; Terrence Higgins Trust<br>Gibbs, Jo; University College London, Research Department of Infection and Population Health; |
| <b>Primary Subject Heading</b>: | HIV/AIDS |
| Secondary Subject Heading: | Health informatics, Infectious diseases |
| Keywords: | HIV & AIDS < INFECTIOUS DISEASES, Telemedicine < BIOTECHNOLOGY & BIOINFORMATICS, GENITOURINARY MEDICINE |
| | |

SCHOLARONE™
Manuscripts

*I, the Submitting Author has the right to grant and does grant on behalf of all authors of the Work (as defined in the below author licence), an exclusive licence and/or a non-exclusive licence for contributions from authors who are: i) UK Crown employees; ii) where BMJ has agreed a CC-BY licence shall apply, and/or iii) in accordance with the terms applicable for US Federal Government officers or employees acting as part of their official duties; on a worldwide, perpetual, irrevocable, royalty-free basis to BMJ Publishing Group Ltd ("BMJ") its licensees and where the relevant Journal is co-owned by BMJ to the co-owners of the Journal, to publish the Work in this journal and any other BMJ products and to exploit all rights, as set out in our licence.*

*The Submitting Author accepts and understands that any supply made under these terms is made by BMJ to the Submitting Author unless you are acting as an employee on behalf of your employer or a postgraduate student of an affiliated institution which is paying any applicable article publishing charge ("APC") for Open Access articles. Where the Submitting Author wishes to make the Work available on an Open Access basis (and intends to pay the relevant APC), the terms of reuse of such Open Access shall be governed by a Creative Commons licence – details of these licences and which Creative Commons licence will apply to this Work are set out in our licence referred to above.*

*Other than as permitted in any relevant BMJ Author's Self Archiving Policies, I confirm this Work has not been accepted for publication elsewhere, is not being considered for publication elsewhere and does not duplicate material already published. I confirm all authors consent to publication of this Work and authorise the granting of this licence.*

For peer review only

**BMJ Open**

# Patient-generated data in the management of HIV: a scoping review

**Authors and affiliations**

Clara Hewitt[1]
Karen C Lloyd[1]
Shema Tariq[1]
Abigail Durrant[2]
Caroline Claisse[2]
Bakita Kasadha[3]
Jo Gibbs[1]

1 Institute for Global Health, University College London, UK
2 Open Lab, School of Computing, Newcastle University, Newcastle upon Tyne, UK
3 Terrence Higgins Trust, London, UK

**Correspondence to:**
Dr. Karen C. Lloyd, Mortimer Market Centre, Off Capper St, Bloomsbury, London WC1E 6JB; K.lloyd@ucl.ac.uk

**Word count** (excluding title page, abstract, tables, contributions and references): 3528

## ABSTRACT

**Objectives:** Patient-generated data (PGData) is an emergent research area and may improve HIV care. The objectives of this scoping review were to synthesise, evaluate and make recommendations based upon the available literature regarding PGData use in HIV care.

**Design:** Scoping review

**Data sources:** Embase, Medline, CINAHL Plus, Web of Science, Scopus, PsycINFO and Emcare databases.

**Eligibility Criteria:** Studies involving PGData use within HIV care for people living with HIV and/or healthcare professionals (HCPs) published before February 2021.

**Data extraction and synthesis:** Data were extracted using a table and the Mixed Methods Appraisal Tool was used to assess empirical rigour. We used Thematic Analysis to evaluate content.

**Results:** 11 articles met the eligibility criteria. Studies were observational, predominantly concerned hypothetical or novel digital platforms, mainly conducted in high-income settings, and had small sample sizes (range=10-160). There were multiple definitions of PGData. In the majority of studies (n=9) participants were people living with HIV, with a few studies including HCPs, informatics specialists, or mixed participant groups. Participants living with HIV were aged 23-78, mostly male, of diverse ethnicities, and had low educational, health literacy and income levels.

We identified four key themes: 1. Perceptions of PGData and associated digital platforms; 2. Opportunities; 3. Anticipated barriers; and 4. Potential impact upon patient-HCP relationships.**Conclusions:** Use of PGData within HIV care warrants further study, especially with regard to digital inequalities, data privacy and security. There is a need for longitudinal data on use within HIV in a variety of settings with a broad range of users, including impact on clinical outcomes. This will allow greater understanding of the role of PGData use in improving the health and wellbeing of people living with HIV, which is increasingly pertinent as digital healthcare becomes more widespread as a result of COVID-19.


**Keywords:** HIV; digital health; self-management; patient generated health data; remote monitoring.

**Strengths and limitations of this study:**

- This scoping review provides an overview and synthesis of the emergent literature of PGData use within HIV care, providing a foundation of understanding to inform future research.
- The Mixed Methods Appraisal Tool allowed assessment of mixed study designs.
- Literature searching yielded a limited number of articles, with most articles obtained via manual referencing.
- Only English studies were reviewed, which may bias and thus narrow the scope of the findings to English speaking settings.



For peer review only - http://bmjopen.bmj.com/site/about/guidelines.xhtml

Antiretroviral therapy (ART) has extended the life expectancy of people living with HIV, transforming HIV into a chronic condition. [1] Effective HIV care requires both long-term clinical follow-up as well as high levels of adherence to ART. This facilitates viral suppression, reducing HIV-related morbidity and mortality, as well as preventing onward HIV transmission. [2] However, HIV care is often complex; people living with HIV have a greater risk of co-morbid conditions such as poor mental health and cardiovascular disease compared to their HIV-negative counterparts. [3] [4] These co-morbidities (often a result of the virus, side effects of ART, and/or complex socioeconomic factors) may require considerable input from a wide range of healthcare professionals (HCPs), which has a consequential impact on healthcare resources, even in high income settings. [1] [5] With health systems already financially and time-poor, it is important to identify innovative approaches to HIV care that can support quality of life, while addressing the challenges posed by increasing co-morbidities and an ageing population. Digital technologies present opportunities through which to improve HIV care, and in turn, the health and quality of life of people living with HIV. [6]

The role of self-management in the care of long-term conditions such as HIV is becoming increasingly important. [6] The ubiquity of digital devices and applications used for health purposes can support self-management and has created fertile grounds for patient generated data (PGData) collection. [7] [8] Although no universal PGData definition exists, multiple authors across the literature have adopted the broad definition [9] that PGData constitute any health-related data which are created or collected by patients or designated proxies to address a health concern. [7] [10] [11] [12] [13] [14] As such, PGData include medication dosages, physical activity, dietary intake, sleep and mood patterns, and can be collected in different modalities including text, pictures, voice or video recordings, and numerical information e.g. questionnaire scores or physiological measurements. [11] [15] PGData allow



For peer review only - http://bmjopen.bmj.com/site/about/guidelines.xhtml

more frequent, remote and longitudinal tracking of multiple types of health information, and can be rapidly shared between patients and HCPs, or between patients themselves, usually using wireless connections. [13] [16] Additionally, PGData can serve as an adjunct to health information communicated during clinical consultations; they can demonstrate baseline health measures bespoke to each individual, which may ultimately facilitate more patient-centred, personalised care. [8] [14] [17] Consequently, PGData allow greater real-time insight into health fluctuations or anomalies relative to a baseline, permitting holistic appreciation of patient health for both patients and their HCPs, and potentially enabling more appropriate and timely interventions. [5] [9] [10] [11] [15] [16] [18]

Whilst the use of PGData for the management of other long-term conditions such as cancer [14] and diabetes [18] has been investigated, to our knowledge, no review of PGData use within HIV care has been conducted to date. This scoping review is timely, given that COVID-19 has led to rapidly increased adoption of remote healthcare and telemedicine practices within clinical care, which will likely remain in place beyond the current pandemic.

**Objectives:**

This scoping review aimed to 1. Synthesise and evaluate the existing emergent literature on PGData use within HIV care; 2. Identify the opportunities and challenges it presents; and 3. Consider the impact of PGData use upon people living with HIV and their HCPs. For the purposes of this review we interpreted ‘HIV care’ as being synonymous with HIV management, and to mean all HIV-related healthcare from the point of diagnosis.



For peer review only - http://bmjopen.bmj.com/site/about/guidelines.xhtml

# METHODS

We conducted a scoping review of literature on PGData use within HIV care. We adopted this approach to enable a broad focus and preliminary synthesis of available evidence [19] in this new, emerging research area. We have reported the review in accordance with the Preferred Reporting Items for Systematic Reviews and Meta-analysis extension for Scoping Reviews (PRISMA-ScR) guidelines [20] (see Appendix A). A review protocol has not been registered.

## Search strategy

During July 2019, we searched Medline, Embase, Emcare, CINAHL, PsycINFO, Scopus and Web of Science databases for articles. Additional grey literature databases were searched using the same strategy: National Institute of Clinical Excellence: Health and Social Care, TRIP medical database, ProQuest Dissertations and Theses, OpenAIRE, Bielefeld Academic Search Engine (BASE), OpenDOAR, Open Access Dissertations and Theses, DART-Europe, Open Grey and Semantic Scholar. Searches were restricted to English language and the terms used were adapted to meet the requirements of each database. Detailed search terms can be found in Appendix B. Article reference lists were also manually searched to obtain additional literature which the formal search strategy may have omitted.

CH conducted the original screenings in conjunction with JG and KL. ST and CH re-ran literature searches of all databases in February 2021 using the same search strategy.No further literature within scope was identified.



For peer review only - http://bmjopen.bmj.com/site/about/guidelines.xhtml

## Eligibility Criteria

Study inclusion was contingent on the terms outlined in Table 1 and temporal limits were not imposed to keep the scope of literature generated as broad as possible given the infancy of the literature base.

We chose to exclude PGData which is collected in response to prompts from external sources e.g. mobile app or SMS reminders. Whilst PGdata prompts are not specifically addressed in the PGdata definition we employed, we made this exclusion on the basis that we believed PGData collected following prompts is not truly 'generated' autonomously by patients. We also employed this exclusion based on our consideration that prompted PGData may mean an individual has a qualitatively different experience of PGData collection and use, and that any prompted PGData collected may not be a true reflection of natural, uninfluenced patient engagement with PGdata e.g. the PGData which individuals would naturally collect unprompted.



For peer review only - http://bmjopen.bmj.com/site/about/guidelines.xhtml

| Inclusion Criteria |
|---|
| • Studies which considered the perspectives of HCPs or people living with HIV regarding PGData use within HIV management<br>• Studies where PGData use met the definition adopted [10]<br>• Where studies involved patients, these needed to be people living with HIV<br>• PGData collection by people living with HIV had to be conducted via a digital platform<br>• PGData collected in relation to living with HIV<br>• Any PGData created had to be collected independently by people living with HIV, and not in response to a reminder or prompt |
| **Exclusion Criteria** |
| • Studies involving PGData which were not generated independently by people living with HIV, or where PGData were created in response to the receipt of a reminder or prompt<br>• Studies concerning PGData use in the diagnosis, prevention or risk reduction of HIV<br>• Studies solely involving a two-way communication system, including studies involving digital messaging or forum platforms<br>• Conference proceedings |

***Table 1.*** **Summary of inclusion and exclusion criteria.**



For peer review only - http://bmjopen.bmj.com/site/about/guidelines.xhtml

## Selection of sources of evidence

The retrieved articles were exported into Mendeley Reference Manager and duplicates were removed. Titles and abstracts were screened, and full texts were assessed using the eligibility criteria. A data extraction table (see Appendix C) was used to summarise data from articles meeting the eligibility criteria for analysis.

## Empirical appraisal and thematic analysis of included articles

Due to heterogeneity of study designs, empirical quality was assessed using the Mixed Methods Appraisal Tool (MMAT, [21] see Appendix D). Thematic analysis [22] was conducted based upon the qualitative findings from the identified articles. We employed an inductive approach due to the nascent nature of this research field.

For the thematic analysis, primarily individual article-specific mind maps were created to highlight key concepts identified per article. [23] Subsequently, a comprehensive mind map was created to synthesise key topics across all 11 reviewed articles. Topics recurring twice or more between articles were highlighted as having thematic importance and these were finally organised into four overarching themes and three sub-themes. Themes were discussed by CH, KL, HG and ST until consensus was reached.

## Patient and public involvement

This manuscript reports on a review of the literature. As such, no patients or members of the public were explicitly involved in the scoping process, however, one co-author is a peer-researcher (with lived experience of HIV) within a third sector organisation.

# RESULTS

## Search results

Original literature searches were conducted in June 2019. A total of 2,353 articles were identified through academic databases and grey literature keyword searching (Figure 1). Seventy-three full-text articles were assessed, of which five fulfilled the eligibility criteria for



For peer review only - http://bmjopen.bmj.com/site/about/guidelines.xhtml

inclusion. A further six eligible studies were identified from a manual search of reference lists. A total of 11 articles were included for final review (see article summaries, Table 2). An updated search conducted in February 2021 did not identify any further eligible studies.

**[Figure 1. PRISMA Flow Diagram]**

## Participants and demographics

Sample sizes of included studies ranged from 10-160 participants. Participants were predominantly people living with HIV, however, some studies involved HCPs, allied HCPs, informatics specialists, or mixed participant groups (see Appendix C). Apart from one study,[24] where nurses and doctors were participants, role specialisms were not identified. Where reported, studies primarily included men who have sex with men (MSM) and heterosexual men and women. The age range of people living with HIV across the studies was 23-78 years. Participants were mostly male and of Black (including Black African and African American) ethnicity. Only two studies involved transgender people living with HIV, despite this being a HIV key population. Where reported, people living with HIV were most often from low-income, low-educational backgrounds.

## Study characteristics

The empirical quality of the 11 articles reviewed was varied (see MMAT assessment, Appendix D). Studies were predominantly conducted in the USA (eight studies [24] [25] [26] [27] [28] [29] [30] [31]), with others based in China [32], England [33] and other parts of Europe [34]. All PGData platforms were mobile or web-based except for one study involving a medication storage device. [32] Prior to analysis, articles were grouped into three categories according to the type of digital platform used; hypothetical, prototype or pre-established (see Table 2) in order to distinguish between the different natures of digital platform types. Hypothetical platforms were those where researchers had developed a PGData platform concept, with no tangible product in existence (5 studies), [24] [27] [30] [33] [34] prototype platforms involved

PGData platforms being piloted (3 studies), [28] [29] [31] and pre-established platforms referred to PGData platforms already in existence which were being trialled (3 studies). [25] [26] [32] Study designs included user testing, [25] [28] [29][32] focus groups [26] [30] [31] and semi-structured interview sessions. [24] [33] [34] One study also included patient-HCP co-design workshops. [34]

User testing studies varied in structure; two studies trialled beta mobile application (app) prototypes for PGData collection wherein people living with HIV and informaticians completed tasks whilst simultaneously voicing feedback, [28] [29] whilst others examined the impact of medication storage device use [32] and a six-week personal health record app training programme for people living with HIV. [25] User testing studies focused on the perceived usability and feasibility of PGData collection and associated digital platforms; some studies employed quantitative assessments to measure these [25] [28] [29] (see Appendix C). No studies conducted user testing with HCPs. Studies conducting interviews and focus group discussions tended to use open-ended questions or follow discussion guides, and centred around issues of PGData platform acceptability, feasibility, and anticipated barriers and facilitators to PGData adoption.

| **Studies concerning hypothetical digital platforms** | | | |
|---|---|---|---|
| ***Author(s)*** | ***Participants, design, location, study length*** | ***Digital platform*** | ***Overview*** |
| **Bussone (2018)** | -N = 16 people living with HIV<br>- Qualitative, individual semi-structured interviews of approximately 30 minutes<br>-England (UK)<br>-Study length unspecified | No specific digital platform used | Aimed to understand how people living with HIV monitor their personal health data. Addressed topics including participants' current self-management behaviours and issues surrounding current, previous or desired means of tracking personal health information. |

| | | | |
|---|---|---|---|
| **Marent, Henwood & Darking (2018)** | -N = 160 participants<br>• 97 people living with HIV<br>• 63 HCPs working in HIV care<br>-40 doctors<br>-10 nurses<br>-4 psychologists<br>-4 pharmacists<br>-2 social workers<br>-2 nutritionists<br>• 1 sexologist<br>- Qualitative, 14 recorded workshops and 22 semi-structured interviews<br>-Brighton (UK), Lisbon (Portugal), Barcelona (Spain), Antwerp (Belgium) and Zagreb (Croatia)<br>-January-June 2016 | No specific digital platform used | Aimed to determine perceptions of, and advantages and concerns surrounding, the development of a mobile app for HIV management. Addressed topics including perceived challenges and barriers to use of a mobile app for HIV management, current mobile app usage and perceived useful features of a mobile app for HIV management. |
| **Nokes et al. (2013)** | -N = 100 people living with HIV or AIDS<br>-Quantitative<br>-New York, USA<br>-Study length unspecified | No specific digital platform used | Aimed to assess self-efficacy of people living with HIV regarding use of a hypothetical personal health record. Participants received an explanation of a personal health record and completed questionnaires measuring their self-efficacy in relation to digital and paper-based personal health records, condom use and chronic disease management. |
| **Ramanathan et al. (2013)** | -N= 29 people living with HIV<br>-Qualitative: focus group discussions<br>-Los Angeles, USA<br>-Study length unspecified | No specific digital platform used | Aimed to assess feature preferences for a mobile app for HIV self-management. Addressed topics including privacy, goal-setting, data capturing methods, feedback regarding app-user behaviours and the role of reminders. |
| **Swendeman et al. (2016)** | - N = 12 HCPs working in HIV care<br>• 3 doctors<br>• 5 nurses<br>• 2 psychosocial case workers<br>• 1 outreach worker<br>• 1 psychotherapist<br>- Qualitative, individual 45-60 minute semi-structured interviews<br>-Los Angeles, USA<br>-Study length unspecified | No specific digital platform used | Aimed to assess HCP attitudes regarding a hypothetical mobile app (for people living with HIV) and online dashboards (for HCPs) for HIV management. Addressed topics including usability, acceptability and barriers to digital platform use. |
| **Studies concerning prototype digital platforms** | | | |

| *Author(s)* | *Participants, design, location, study length* | *Digital platform* | *Overview* |
|---|---|---|---|
| **Schnall et al. (2011)** | - N = 37 Case Managers working with people living with HIV<br>- Qualitative, one-hour focus group discussions<br>-New York, USA<br>- March-December 2008 | SelectHealth Continuity of Care Document; includes key health information including latest medical test results, pharmaceutical record, visits to healthcare facilities and patient contact information | Aimed to determine factors influencing usage and acceptability of online "SelectHealth Continuity of Care Document" for people living with HIV, before the platform's release. Addressed topics including facilitators and barriers to use of the continuity of care record. |
| **Schnall et al. (2016)** | -N = 15<br>• 10 people living with HVI<br>• 5 informaticians<br>- Mixed: quantitative, qualitative<br>-New York, USA<br>-Study length unspecified | No description of app prototype provided | Aimed to test a prototype mobile app designed to facilitate HIV management in people living with HIV. Participants received a description of a mobile app prototype for HIV management. Informaticians tested the model for 45-90 minutes and completed a Heuristic Evaluation Checklist<br>people living with HIV evaluated prototype app screenings and completed the Post Study System Usability Questionnaire. |
| **Stonbraker et al. (2018)** | -N= 25<br>• 20 people living with HIV who possessed at least one HIV-associated non-AIDS condition<br>• 5 informaticians<br>-Mixed: quantitative; qualitative<br>-New York, USA | -Novel mobile app "VIP-HANA" (Video Information Provider (VIP) for HIV-associated non-AIDS conditions) | Aimed to determine usability of novel mobile app "VIP-HANA" designed to aid people living with HIV with management of HIV/non-HIV-related symptoms, and to determine where improvements to the app were needed. After receiving descriptions of the app participants were assigned tasks on a beta version of the app and had to describe aloud their thoughts and actions whilst their activities were tracked. People living with HIV completed one questionnaire to assess their health literacy level, and two questionnaires relating to the app's usability. |
| **Studies concerning pre-established digital platforms** | | | |
| *Author(s)* | *Participants, design,<br><br>location, study length* | *Digital platform* | *Overview* |

| | | | |
|---|---|---|---|
| **DeSilva et al. (2012)** | -N= 10 people living with HIV who used injection drugs<br>-Individual qualitative interviews<br>-Nanning, China<br>-Study length unspecified | *Wisepill* medication storage container; once opened a wireless signal immediately transmits to a server, the data of which can be obtained by HCPs | Aimed to determine the feasibility and acceptability of using a "Wisepill" device in injecting drug users who were HIV positive. Participants used the "Wisepill" device to monitor their antiretroviral medication adherence for one month. Addressed topics including acceptability, feasibility and usability of the device. |
| **Luque et al. (2013)** | -N=29 people living with HIV<br>-Quantitative<br>-Rochester, New York, USA<br>-6 weeks | *MyMedical* app, (downloaded from iTunes Store). | Aimed to determine acceptability and usability of using the *MyMedical* app as a personal health record on an iPod touch device, and to observe the effect of using a digital personal health record upon HIV treatment self-efficacy. Participants underwent six 90-minute training sessions and were given 'homework' tasks to rehearse between training sessions. |
| **Odlum et al. (2014)** | -N= 57 people living with HIV<br>• Quantitative: 42 people living with HIV<br>• Qualitative: 15 people living with HIV; 8 users of the *MyHealthProfile* app, 7 non-users of the *MyHealthProfile* app<br>-Mixed: quantitative, qualitative<br>-New York, USA<br>-Study length unspecified | *MyHealthProfile* app; acts as a continuity of care people for living with HIV to track their personal health record via an internet connection | Aimed to determine usability of the *MyHealthProfile* app and to determine where improvements could be made to inform a new version of the app named *MyHealthProfile-plus*. Participants completed 2 surveys relating to the usefulness and content of the pre-established *My Health Profile* app. Participants completed 60 minute focus groups regarding the development of new *My Health Profile-plus* app |

***Table 2.*** **Summary of included articles**

**Overview of Key Themes**

Our analysis highlighted four primary cross-cutting themes and three sub-themes, including: perceived acceptability, feasibility and usability of PGData; opportunities presented by PGData; anticipated barriers to PGData use; and how PGData may impact HIV care and HCP-patient relationships.

**Acceptability, feasibility and usability of digital platforms for PGData**

Participants' views regarding the acceptability, feasibility and usability of PGData differed. Some people living with HIV were receptive to digitally tracking their PGData, though this was contingent upon data security. [30] Widespread smartphone access, absence of PGData platform cost, clear design and easy navigability of digital PGData platforms increased the perceived feasibility of PGData use. [24] [26] [32] [33]

Perceptions regarding the perceived suitability of PGData use for people living with HIV varied; some HCPs suggested PGData use would be better suited to newly-diagnosed individuals, those with multi-morbidities, or higher education levels [24] [34] whilst others proposed newly-diagnosed people living with HIV may be unsuitable. [34] Some HCPs questioned whether individual differences including motivation may affect PGData collection and engagement. [24] No data from people living with HIV regarding suitability were reported.

**Opportunities presented by PGData for HIV care**

Participants cited several perceived benefits of PGData use within HIV care, including reduced financial and time burdens, [31] convenience, and increased health proactivity. [26] [31] [33] Some people living with HIV perceived PGData collection could increase their medication adherence, perceived control, understanding and engagement surrounding their HIV care [33] due to their awareness of ongoing PGData monitoring. [24] [30] [32]

**Anticipated barriers and challenges associated with PGData**

Across the studies, several perceived challenges to PGData use emerged, including privacy and security, accessibility and reliability of PGData, and impact on HCPs.

<u>Privacy and security</u>

Participants expressed substantial privacy and security concerns regarding PGData collection and analysis, [25] [26] [30] [31] which some authors attributed to persisting HIV stigma.



For peer review only - http://bmjopen.bmj.com/site/about/guidelines.xhtml

[31] [32] [34] Participants voiced concerns that poor security and privacy may result in unwanted disclosures of sensitive data including HIV status, although how this might occur was not explicitly articulated. [30] [31][32] [34] Several people living with HIV emphasised desires for security mechanisms including encryption, [30] [33] [34] and some people living with HIV favoured paper-based PGData tracking methods due to digital platform apprehension. [33]

Accessibility and reliability of digital infrastructure and collected PGData

There were prevalent concerns regarding the reliability of digital platforms and PGData itself. Some studies reported concerns that technological disruptions and financial instability may hinder digital platform accessibility and usage. [24] [32] Additionally, some participants highlighted PGData reliability could be impaired by incorrect PGData recording. [31] Some people living with HIV warned of intentional retrospective editing to deliberately manipulate PGData. [30] [33]

Impact upon HCPs

HCPs were apprehensive that pre-existing time constraints and large workloads would hinder PGData-reviewing and that PGData involvement would only magnify these burdens. [24] [34] People living with HIV did not comment on the impact of PGData upon HCPs.

**Perceived impacts of PGData upon patient-HCP relationships**

Participants felt that engaging with PGData within clinical consultations could facilitate a more comprehensive picture of how a person may be managing their HIV. [24] [33] [34] Whilst people living with HIV desired PGData platforms allowing two-way communication with HCPs, HCPs feared increased workloads. [34]

Some people living with HIV thought that PGData use may provide opportunities to strengthen patient-HCP relationships. [34] However contrastingly, other participants expressed concern that PGData misinterpretation could impair pre-established face-to-face patient-HCP interactions. [30] [34] Both people living with HIV and HCPs believed PGData use could



For peer review only - http://bmjopen.bmj.com/site/about/guidelines.xhtml

complement, rather than supersede, traditional in-person HIV consultations, although details of such a hybrid model were absent. [34]

## DISCUSSION

This review provides a critical synthesis of the nascent literature on PGData use specifically for HIV care, and highlights several unanswered questions. Across the eleven articles, study designs and methodologies were varied and a spectrum of digital PGData platforms were considered. The views of people living with HIV and HCPs were broadly consistent, apart from on the potential impact of PGData upon patient-HCP relationships.

**Individual and contextual differences**

Our findings suggest that PGdata use may not be suitable for all people living with HIV; indeed, perceptions of PGData and its use within HIV care may differ greatly across patients and contexts. [35] For example, whilst some patients felt reassured by monitoring their long-term health condition, for others, health monitoring could be tiring, intrusive, anxiety-provoking and excessively time-consuming. [9][30] [36] [37] It is important that digital health interventions to support HIV care and self-management do not further reinforce stigma and use language that is accessible to all users. [38] As the majority of studies did not include participants from key HIV populations, such as women, people who are transgender, or people who inject drugs, knowledge of PGData use in these communities remains limited. [15] The current review's bias towards high-income settings further limits its generalisability given the global HIV burden lies predominantly in low and middle-income settings. [39] Furthermore, digital inequalities due to limited access and poor digital literacy [40] [41] will impact acceptability, feasibility and inequalities in PGData use, with the COVID-19 pandemic starkly highlighting these digital divides.[42] [43] Further research is needed to better understand these individual and contextual nuances.



For peer review only - http://bmjopen.bmj.com/site/about/guidelines.xhtml

### Impact upon patient-HCP relationships

Our findings highlight dichotomies between people living with HIV and HCPs; whilst people living with HIV appeared to welcome increased HCP contact and HCP-analysed PGData, HCPs expressed concerns around additional workload and expectations. [11] [14] [16] [24] [34] [44] [45] A PGData guidance framework may be necessary to counteract competing priorities between HCPs and patients regarding such communication preferences. [9] [12] West and colleagues suggest one possible workflow model to address these concerns.[46] PGData use within HIV care may also impact patients' levels of responsibility, autonomy and control over their healthcare relative to HCPs. [37] [47] Thus, it seems plausible that PGData will impact relationships between people living with HIV and HCPs. The extent of this impact is presently unknown, but it may vary in its effect on both patients and HCPs, as well as across settings and cultural contexts.

### Barriers to PGData uptake

Across the studies concerns from both patients and HCPs regarding the privacy, security and reliability of PGData were common, indicating considerable apprehension around the use of PGData. Regarding PGData platforms, in this review mobile apps were the most-used digital platform; however, more broadly, apps have received repeated criticism for lacking regulation and approval for medical use, potentially threatening the quality, validity and reliability of mobile-collected data. [36] [48] [49] [50] [51] [52] Furthermore, many apps lack privacy policies or data encryption measures, potentially risking data exploitation, theft or unauthorised access. [49] [53] [54]

Given the prominent PGData privacy and security concerns highlighted in this review, without adequate regulatory, privacy and security protocols, people living with HIV may be less inclined to engage with PGData. Furthermore, both data security mistrust and social desirability, wherein patients may under or over-report actions to appear more socially



For peer review only - http://bmjopen.bmj.com/site/about/guidelines.xhtml

acceptable, may increase the likelihood of deliberate PGData manipulation, degrading its reliability. [47] [53] Ultimately, PGData adoption into HIV care may be jeopardised if concerns regarding the safety, privacy and trustworthiness of the platforms used to collect PGData, or the PGData itself, prevail amongst people living with HIV and HCPs. [50] [54]

**Limitations**

Terminology and Search Strategy

The small article yield of 11 articles has several possible explanations. Many articles were out of scope and considered ineligible; inconsistencies in PGData terminology across the literature [55] may have exacerbated this and potentially rendered relevant articles unidentifiable using the current review's search strategy. Excluding conference proceedings, coupled with challenges in locating full-text articles, may mean some studies were overlooked. Consequently, the structured search strategy proved less fruitful in yielding articles relative to manual reference-searching, a finding other authors have observed. [56] Additionally, author repetition across the studies calls into question whether the same, or similarly-recruited, samples were used between studies, raising further generalisability concerns.

Lack of real-world evidence

The majority of studies were exploratory, involving hypothetical or pilot platforms, which limits their ecological validity and applicability. Additionally, the heterogeneity of PGData platforms used prevents their direct comparison and evaluation. Although the digital platforms addressed were biased to mobile phones, this reflects the wider digital health literature and this platform is more established than other recent technological developments (e.g. wearable devices). [36] Finally, although PGData use may require training, the one training study [25] involved such high training intensity that its real-world feasibility is unlikely.

**Future research recommendations**

Based upon this review, the following four areas warrant further research:

1. Real-world and longitudinal evidence

Resulting from the absence of real-world, longitudinal evidence of PGData use in HIV care, a full-scale pragmatic trial is necessary to enhance the research's ecological validity and enable greater insight into practical issues surrounding PGData usage within HIV care. The impact of any of the PGData platform categories we identified- pilot, hypothetical or novel- could be explored in trial settings.

2. Impact of PGData use upon clinical outcomes

The direct relationship between PGData use and clinical outcomes in people living with HIV, such as medication adherence, quality of life or mental wellbeing, remains unknown and requires investigation.

3. Digital platforms and PGData types

The effectiveness and optimal digital platform type(s) for PGData collection and monitoring requires evaluation. Additionally, exploration of the most useful types of PGData to track is needed.

4. Different contexts and HIV populations

Research exploring PGData use within HIV care in more diverse populations of people living with HIV is critically needed to increase the literature's representativeness for HIV key populations and low and middle-income settings. Additionally, a greater understanding of how PGData use may fluctuate over time is needed. Future research must improve the scope of geographical and patient contexts to avoid further magnifying research inequities regarding PGData use within HIV care. [5] [57]



For peer review only - http://bmjopen.bmj.com/site/about/guidelines.xhtml

## CONCLUSIONS

The emerging PGData field presents opportunities to improve patient self-management and personalised care, which are both increasingly needed due to health system resource constraints and the complexities of HIV care. In recent months, HIV care, services and people living with HIV worldwide have been negatively impacted by the COVID-19 pandemic, causing decreased HIV testing, disrupted antiretroviral treatment procurement and poorer psychological wellbeing of people living with HIV. [58] [59] However, the pandemic has highlighted the value of, and significant shift towards, digital healthcare delivery, [60] [61] which may provide expedited leverage for further PGData research. Going forwards, the real-world applicability of PGData within HIV care must be elucidated, including navigating the heterogeneity and optimal suitability of PGData platforms for people living with HIV within different contexts. Additionally, PGData integration within HIV care will be conditional upon adequately addressing outstanding privacy, data security, reliability and HCP-patient relationship concerns prior to implementation. [14] [62] Overall, PGData may hold the potential to transform HIV care, in turn realising improvements in supporting not only people living with HIV but also the wider health systems in which this care is provided.



For peer review only - http://bmjopen.bmj.com/site/about/guidelines.xhtml

- **Contributors**: CH, KCL, ST, AD, CC, BK and JG contributed to the conception and/or design of the study. CH led, and KCL and JG contributed to the establishment of the search strategy and method of analysis. Article reviewing and data analysis was led by CH with contribution from KCL and JG. CH drafted the original manuscript with supervision and guidance from JG, KCL and ST. All authors (CC, KCL, ST, AD, CC, BK and JG) have made contributions to the drafting and revising of the article, and have approved the final version.

- **Funding**: This research received no specific grant from any funding agency in the public, commercial or not-for-profit sectors. All authors (with the exception of Clara Hewitt) receive research funding from the INTUIT (Interaction Design for Trusted Sharing of Personal Health Data to Live Well with HIV) research project, which is funded by the Engineering and Physical Sciences Research Council (EP/R033900/1).

- **Data availability statement:** This manuscript reports on a scoping review. All articles included in the sample are included in the article and/or as supplementary material.
- **Competing interests:** None declared.
- **Patient consent:** Not applicable.
- **Ethics approval:** Not applicable.

# REFERENCES

[1] Deeks, S. G., Lewin, S. R. & Havlir, D. V. (2013). The end of AIDS: HIV infection as a chronic disease. *The Lancet*, 382 (9903): pp. 1525-1533.
[2] Claborn, K. R., Fernandez, A., Wray, T. & Ramsey S. (2015). Computer-based HIV adherence promotion interventions: a systematic review. *Translation Behavioral Medicine*, 5 (3): pp. 294–306.
[3] Hunt, P. W. (2014). HIV and aging: Emerging research issues. *Current Opinion in HIV and AIDS*, 9 (4): pp. 302–308.
[4] Marcus, J. L., Leyden, W. A., Alexeeff, S. E., Anderson, A. N., Hechter, R. C., Hu, H., Lam, J. O., Towner, W. J., Yuan ,Q., Horberg, M. A. & Silverberg, M. J. (2020). Comparison of Overall and Comorbidity-Free Life Expectancy Between Insured Adults With and Without HIV Infection, 2000-2016. *JAMA Network* Open, 3 (6): e207954.
[5] Claborn, K. R., Meier, E., Miller, M. B., Leavens, E. L., Brett, E. I. & Leffingwell, T. (2018). Improving adoption and acceptability of digital health interventions for HIV disease management: a qualitative study. *Translational Behavioral Medicine,* 8 (2): pp. 268-279.
[6] Cooper, V., Clatworthy, J. & Whetham, J. (2017). mHealth Interventions To Support Self- Management In HIV: A Systematic Review. *The Open AIDS Journal,* 11: pp. 119-132.
[7] Nittas, V., Mütsch, M., Ehrler, F. & Puhan, M. A. (2018). Electronic patient-generated health data to facilitate prevention and health promotion: a scoping review protocol. *BMJ Open,* 8: pp. e021245.
[8] Park, Y. R., Lee, Y., Kim, J. Y., Kim, J., Kim, H. R., Kim, Y. H., Kim, W. S. & Lee, J. H. (2018). Managing Patient-Generated Health Data Through Mobile Personal Health Records: Analysis of Usage Data. *JMIR Mhealth and Uhealth*, 6 (4): pp. e89.
[9] Shapiro, M., Johnston, D., Wald, J. & Mon, D. (2012). Patient-Generated Health Data. White Paper prepared for Office of Policy and Planning Office of the National Coordinator for Health Information Technology. Available from: https://www.healthit.gov/sites/default/files/rti_pghd_whitepaper_april_2012.pdf. [Accessed 5th June 2019].
[10] Reading, M. & Merrill, J. (2018). Converging and diverging needs between patients and providers who are collecting and using patient-generated health data: An integrative review. *Journal of the American Medical Informatics Association,* 25 (6): pp. 759-771.
[11] Wood, W. A., Bennett, A. V. & Basch, E. (2015). Emerging uses of patient generated health data in clinical research. *Molecular Oncology*, 9 (5): pp. 1018–1024.
[12] Cohen, D. J., Keller, S. R., Hayes, G. R., Dorr, D. A., Ash, J. S. & Sittig, D. F. (2016). Integrating Patient-Generated Health Data into Clinical Care Settings or Clinical Decision-Making: Lessons Learned from Project HealthDesign. *JMIR Human Factors*, 3 (2): pp. e26.
[13] Chung, A. E. & Basch, E. M. (2015). Potential and Challenges of Patient-Generated Health Data for High-Quality Cancer Care. *Journal of Oncology Practice,* 11 (3): pp. 195-197.
[14] Murthy, H. & Wood, S. (2015). The Value of Patient Reported Outcomes and Other Patient-Generated Health Data in Clinical Hematology. *Current Hematologic Malignancy Reports,* 10 (3): pp. 213-224.
[15] Burns, K. (2017). Engagement, empowerment and patient generated health data. PhD thesis, Queensland University of Technology. Available from: https://eprints.qut.edu.au/114076/1/Kara%20Burns%20Thesis.pdf.
[16] Zhu, H., Colgan, J., Reddy, M. & Choe, E. K. (2017). Sharing Patient-Generated Data in Clinical Practices: An Interview Study. *AMIA Annual Symposium Proceedings Archive*. 2016: pp. 1303–1312.



For peer review only - http://bmjopen.bmj.com/site/about/guidelines.xhtml

[17] Torkamani, A., Andersen, K. G., Steinhubl, S. R. & Topol, E. J. (2017). High-definition medicine. *Cell,* 170 (5): pp. 828-843.
[18] Nundy, S., Lu, C.-Y. E., Hogan, P., Mishra, A. & Peek, M. E. (2014). Using Patient-Generated Health Data from Mobile Technologies for Diabetes Self-Management Support: Provider Perspectives from an Academic Medical Center. *Journal of Diabetes Science and Technology*, 8 (1): pp. 74-82.
[19] Pham et al., 2014 Pham, M. T., Rajić, A., Greig, J. D., Sargeant, J. M., Papadopoulos, A., & McEwen, S. A. (2014). A scoping review of scoping reviews: advancing the approach and enhancing the consistency. *Research synthesis methods*, *5*(4), 371–385
[20] Tricco, A. C., Lillie, E., Zarin, W., O'Brien, K. K., Colquhoun, H., Levac, D., Moher, D., Peters, M., Horsley, T., Weeks, L., Hempel, S., Akl, E. A., Chang, C., McGowan, J., Stewart, L., Hartling, L., Aldcroft, A., Wilson, M. G., Garritty, C., Lewin, S., Godfrey, C. M., Macdonald, M. T., Lanlois, E. V., Soares-Weiser, K., Moriarty, J., Clifford, T., Tunçalp, Ö & Straus, S. E. (2018). PRISMA Extension for Scoping Reviews (PRISMA-ScR): Checklist and Explanation. *Annals of Internal Medicine*, 169 (7): pp. 467–473. Available from: https://www.equator-network.org/reporting-guidelines/prisma-scr/
[21] Hong, Q. N., Pluye, P., Fàbregues, S., Bartlett, G., Boardman, F., Cargo, M., Dagenais, P., Gagnon, M.-P., Griffiths, F., Nicolau, B., O'Cathain, A., Rousseau, M.-C. & Vedel, I. (2018). Mixed Methods Appraisal Tool Version 2018 User Guide. Available from: http://mixedmethodsappraisaltoolpublic.pbworks.com/w/file/fetch/127916259/MMAT_2018_criteria-manual_2018-08-01_ENG.pdf. [Accessed 10 June 2019].
[22] Braun, V. & Clarke, V. (2006). Using thematic analysis in psychology. *Qualitative Research in Psychology*. 3 (2): pp. 77-101.
[23] Wheeldon, J. & Åhlberg, M. K. (2012). Mapping mixed-methods research: theories, models, and measures. In Wheeldon, J., & Åhlberg, M. K. *Visualizing Social Science Research: Maps, Methods, & Meaning* (pp. 113-148). Thousand Oaks, CA: SAGE Publications, Inc.
[24] Swendeman, D., Farmer, S., Mindry, D., Lee, S. J. & Medich, M. (2016). HIV Care Providers' Attitudes regarding Mobile Phone Applications and Web-Based Dashboards to support Patient Self-Management and Care Coordination: Results from a Qualitative Feasibility Study. *Journal of HIV and AIDS*, 2 (4): pp. 10.
[25] Luque, A. E., Corales, R., Fowler, R. J., Dimarco, J., Van Keken, A., Winters, P., Keefer, M. C. & Fiscella, K. (2013). Bridging the Digital Divide in HIV Care: A Pilot Study of an iPod Personal Health Record. *Journal of the International Association of Providers of AIDS Care (JIAPAC),* 12 (2): pp. 117-121
[26] Odlum, M., Gordon, P., Camhi, E., Valdez, E. & Bakken, S. (2014). Exploring Factors Related to the Adoption and Acceptance of an Internet-based Electronic Personal Health Management Tool (EPHMT) in a Low Income, Special Needs Population of People Living with HIV and AIDS in New York City, *Studies in Health Technology and Informatics,* 201 (1): pp. 145-152.
[27] Nokes, K., Verkuilen, J., Hickey, D., James-Borga, J. & Shan, J. (2013). Developing a personal health record self-efficacy tool. *Applied Nursing Research,* 26 (1): pp. 32-9.
[28] Schnall, R., Bakken, S., Brown Iii, W., Carballo-Dieguez, A. & Iribarren, S. (2016). Usabilty Evaluation of a Prototype Mobile App for Health Management for Persons Living with HIV. *Studies in Health Technology and Informatics*, 225 (1): pp. 481–485.
[29] Stonbraker, S., Cho, H., Hermosi, G., Pichon, A. & Schnall, R. (2018). Usability Testing of a mHealth App to Support Self-Management of HIV-Associated Non-AIDS Related Symptoms. *Studies in Health Technology and Informatics*, 250 (1): pp. 106–110.
[30] Ramanathan, N., Swendeman, D., Comulada, W. S., Estrin, D. & Rotheram-Borus, M. J. (2013). Identifying preferences for mobile health applications for self-monitoring and self-



For peer review only - http://bmjopen.bmj.com/site/about/guidelines.xhtml

management: Focus group findings from HIV-positive persons and young mothers. *International Journal of Medical Informatics,* 82 (4): pp. 38-46.
[31] Schnall, R., Gordon, P., Camhi, E. & Bakken, S. (2011). Perceptions of factors influencing use of an electronic record for case management of persons living with HIV. *AIDS Care,* 23 (3): pp. 357-365.
[32] DeSilva, M. B., Gifford, A. L., Xu, K., Zhong, L., Feng, C., Brooks, M., Harrold, M., Yueying, H., Gill, C. J., Wubin, X., Vian, T., Haberer, J., Bangsberg, D. & Sabin, L. (2013). *AIDS Research and Treatment,* Article ID: 957862.
[33] Bussone, A. (2018). Reflection and personal health informatics for people living with HIV. PhD Thesis, City University of London.
[34] Marent, B., Henwood, F. & Darking, M. (2018). Development of an mHealth platform for HIV Care: Gathering User Perspectives Through Co-Design Workshops and Interviews. *JMIR mHealth and uHealth*, 6 (10): pp. e184.
[35] Robinson, L., Cotten, S., Ono, H., Quan-Haase, A., Mesch, G., Chen, W., Schulz, J., Hale, T. M. & Stern, M. (2015). Digital inequalities and why they matter. *Information, Communication & Society,* 18 (5): pp. 1-14.
[36] Piwek, L., Ellis, D. A., Andrews, S. & Joinson, A. (2016). The Rise of Consumer Health Wearables: Promises and Barriers. *PLoS Medicine,* 13 (2): pp. e1001953.
[37] Lupton, D. & Maslen, S. (2019). How Women Use Digital Technologies for Health: Qualitative Interview and Focus Group Study. *Journal of Medical Internet Research*, 21 (1): pp. e11481.
[38] Singh, A., Gibbs, J., Estcourt, C., Sonnenberg, P. & Blandford, A. (2017). DH '17: Proceedings of the 2017 International Conference on Digital Health July 2017 Pages 6–15. DOI: https://doi.org/10.1145/3079452.3079469.
[39] Fettig, J., Swaminathan, M., Murrill, C. S. & Kaplan, J. E. (2014). Global epidemiology of HIV. *Infectious Disease Clinics of North America*. 28 (3): pp. 323-337.
[40] McAuley, A. (2014). Digital health interventions: widening access or widening inequalities? *Public Health,* 128 (12): pp. 1118-1120.
[41] Jongbloed, K., Parmar, S., van der Kop, M., Spittal, P. M., & Lester, R. T. (2015). Recent Evidence for Emerging Digital Technologies to Support Global HIV Engagement in Care. *Current HIV/AIDS reports*, 12 (4): pp. 451–461.
[42] Beaunoyer, E., Dupéré, S. & Guitton, M. J. (2020). COVID-19 and digital inequalities: Reciprocal impacts and mitigation strategies. *Computers in Human Behaviour,* 111.
[43] Watts, G. (2020). COVID-19 and the digital divide in the UK. *The Lancet Digital Health,* 2 (8): E395-396.
[44] Liu, A., Coleman, K., Bojan, K., Serrano, P. A., Oyedele, T., Garcia, A., Enriquez-Bruce, E., Emmanuel, P., Jones, J., Sullivan, P., Hightow-Weidman, L., Buchbinder, S. & Scott, H. (2019). Developing a Mobile App (LYNX) to Support Linkage to HIV/Sexually Transmitted Infection Testing and Pre-Exposure Prophylaxis for Young Men Who Have Sex with Men: Protocol for a Randomized Controlled Trial. *JMIR research Protocols*, 8 (1): pp. e10659.
[45] McGraw, D., Belfort, R., Pfister, H. & Ingargiola, S. (2013). Going digital with patients: Managing potential liability risks of patient-generated electronic health information. *Journal of Participatory Medicine*. 18 (5): pp. e41. Available from: http://www.jopm.org/perspective/narratives/2013/12/18/going-digital-with-patients-managing-potential-liability-risks-of-patient-generated-electronic-health-information. [Accessed 20 July 2019].
[46] West, P., Van Kleek, M., Giordano, R., Weal, M. J. & Shadbolt, N. (2018). CHI '18: Proceedings of the 2018 CHI Conference on Human Factors in Computing SystemsApril 2018 Paper No.: 484 Pages 1–13, DOI: https://doi.org/10.1145/3173574.3174058



For peer review only - http://bmjopen.bmj.com/site/about/guidelines.xhtml

[47] Huba, N. & Zhang, Y. (2012). Designing patient-centred personal health records (PHRs): Health care professionals' perspective on patient-generated data. *Journal of Medical Systems,* 36 (6): pp. 3893-3905.
[48] Mehraeen, E., Safdari, R., Mohammadzadeh, N., Seyedalinaghi, S., Forootan, S. & Mohran, M. (2018). Mobile-Based Applications and Functionalities for Self-Management of People Living with HIV. *Health Informatics Meets eHealth,* 248: pp. 172-179.
[49] Torous, J., Roberts, L. W. (2017). Needed Innovation in Digital Health and Smartphone Applications for Mental Health: Transparency and Trust. *JAMA Psychiatry*, 74 (5): pp. 437-438.
[50] Huckvale, K., Prieto, J. T., Tilney, M., Benghozi, P. J. & Car, J. (2015). Unaddressed privacy risks in accredited health and wellness apps: a cross-sectional systematic assessment. *BMC Medicine*, 13: pp. 214.
[51] Gibbs, J., Gkatzidou, V., Tickle, L., Manning, S. R., Tilakkumar, T., Hone, K., Ashcroft, R. E., Sonnenberg, P., Sadiq, S. T. & Estcourt, C. S. (2017). 'Can you recommend any good STI apps?' A review of content, accuracy and comprehensiveness of current mobile medical applications for STIs and related genital infections. *Sexually Transmitted Infections,* 93 (4): pp. 234-235.
[52] Effy, V., Tobias, H., Afua, A. & Alessandro, B. (2018). Digital health: meeting the ethical and policy challenges. *Swiss Medical Weekly,* 148: pp. w14571.
[53] Beauclair, R., Meng, F., Deprez, N., Temmerman, M., Welte, A., Hens, N. & Delva, W. (2013). Evaluating audio computer assisted self-interviews in urban south African communities: Evidence for good suitability and reduced social desirability bias of a cross-sectional survey on sexual behaviour. *BMC Medical Research Methodology,* 13 (1): pp.11.
[54] Powell, A. C., Landman, A. B. & Bates, D. W. In search of a few good apps. (2014). *JAMA,* 311 (18): pp. 1851-1852.
[55] Lordon, R. J., Mikles, S. P., Kneale, L., Evans, H. L., Munson, S. A., Backonja, U. & Lober, W. B. (2020). How patient-generated health data and patient-reported outcomes affect patient-clinician relationships: A systematic review. *Health Informatics Journal,* 1460458220928184
[56] Greenhalgh, T. & Peacock, R. (2005). Effectiveness and efficiency of search methods in systematic reviews of complex evidence: audit of primary sources. *BMJ,* 331: pp. 1064
[57] Sinha, C. & Schryer-Roy, A. M. (2018). Digital health, gender and health equity: invisible imperatives. (2018). *Journal of Public Health (Oxford)*. 40 (2): pp. 21-25.
[58] Jiang, H., Zhou, Y. & Tang, W. (2020). Maintining HIV care during the COVID-19 pandemic. *The Lancet HIV,* 7 (5): pp. e308-e309.
[59] Kowalska, J. D., Skrzat-Klapaczyńska, A., Bursa, D., Balayan, T., Begovac, J., Chkhartishvili, N., Gokengin, D., Harxhi, A., Jilich, D., Jevtovic, D., Kase, K., Lakatos, B., Matulionyte, R., Mulabdic, V., Nagit, A., Papadopoulos, A., Stefanovic, M., Vassilenko, A., Vasylyev, M., Yancheva, N., Yurin, O. & Horban, A., for the ECEE Network Group. (2020). HIV care in times of the COVID-19 crisis - Where are we now in Central and Eastern Europe? *International Journal of Infectious Diseases,* 96: pp. 311–314.
[60] Watson, A. R., Wah, R. & Thamman, R. (2020). The Value of Remote Monitoring for the COVID-19 Pandemic. *Telemedicine and e-Health. Ahead of print.*
[61] Peek, N., Sujan, M. & Scott, P. (2020). Digital health and care in pandemic times: impact of COVID-19. *BMJ Health & Care Informatics,* 27: e100166.
[62] Charani, E., Castro-Sánchez, E., Moore, L. S. & Holmes, A. (2014). Do smartphone applications in healthcare require a governance and legal framework? It depends on the application! *BMC Medicine,* 12 (1): pp. 29.



For peer review only - http://bmjopen.bmj.com/site/about/guidelines.xhtml

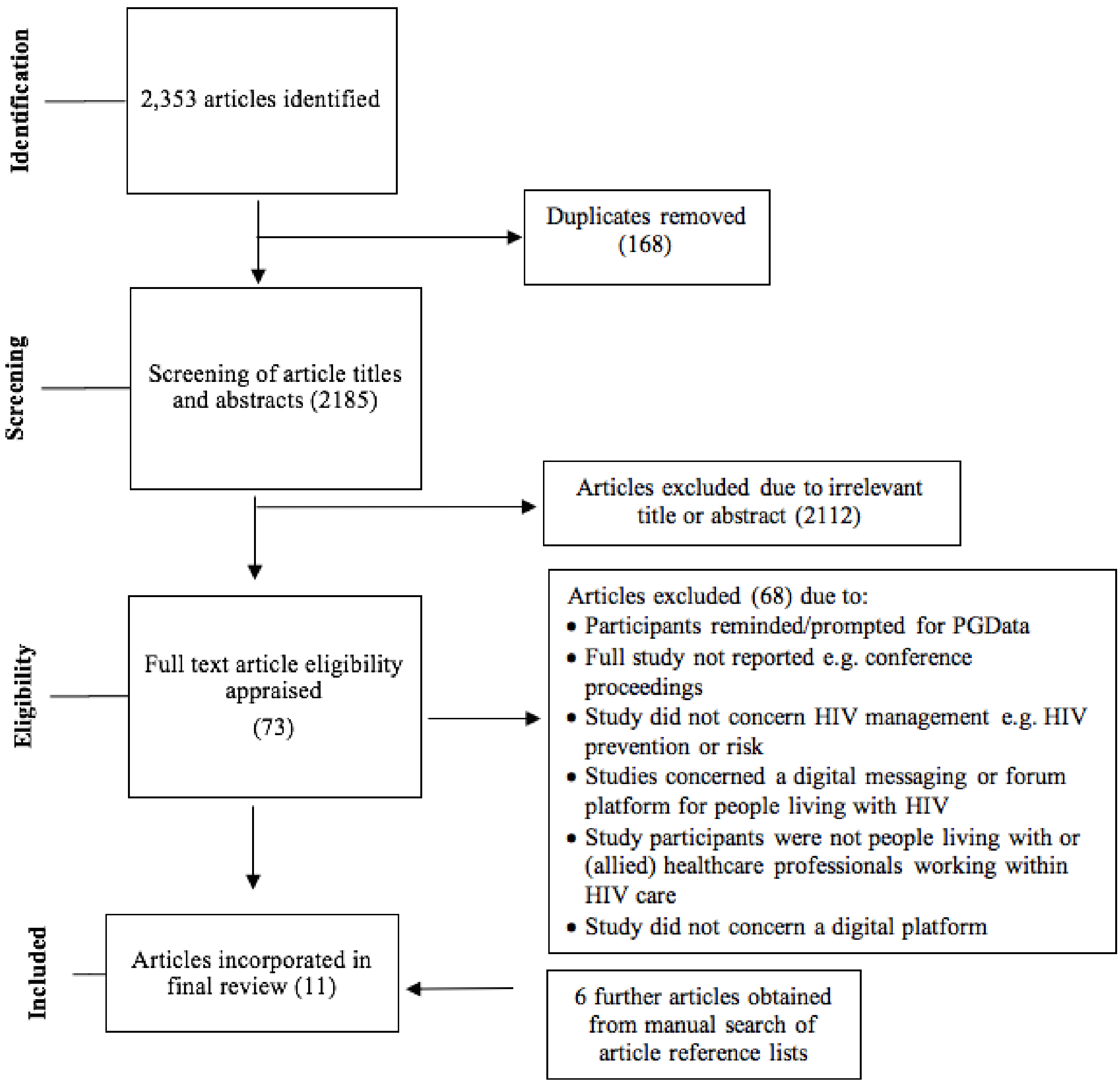


**Figure 1.** ***PRISMA flow diagram.***

only

# Preferred Reporting Items for Systematic reviews and Meta-Analyses extension for Scoping Reviews (PRISMA-ScR) Checklist

| SECTION | ITEM | PRISMA-ScR CHECKLIST ITEM | REPORTED ON PAGE # |
|---|---|---|---|
| **TITLE** | | | |
| Title | 1 | Identify the report as a scoping review. | |
| **ABSTRACT** | | | |
| Structured summary | 2 | Provide a structured summary that includes (as applicable): background, objectives, eligibility criteria, sources of evidence, charting methods, results, and conclusions that relate to the review questions and objectives. | |
| **INTRODUCTION** | | | |
| Rationale | 3 | Describe the rationale for the review in the context of what is already known. Explain why the review questions/objectives lend themselves to a scoping review approach. | |
| Objectives | 4 | Provide an explicit statement of the questions and objectives being addressed with reference to their key elements (e.g., population or participants, concepts, and context) or other relevant key elements used to conceptualize the review questions and/or objectives. | |
| **METHODS** | | | |
| Protocol and registration | 5 | Indicate whether a review protocol exists; state if and where it can be accessed (e.g., a Web address); and if available, provide registration information, including the registration number. | |
| Eligibility criteria | 6 | Specify characteristics of the sources of evidence used as eligibility criteria (e.g., years considered, language, and publication status), and provide a rationale. | |
| Information sources* | 7 | Describe all information sources in the search (e.g., databases with dates of coverage and contact with authors to identify additional sources), as well as the date the most recent search was executed. | |
| Search | 8 | Present the full electronic search strategy for at least 1 database, including any limits used, such that it could be repeated. | |
| Selection of sources of evidence† | 9 | State the process for selecting sources of evidence (i.e., screening and eligibility) included in the scoping review. | |
| Data charting process‡ | 10 | Describe the methods of charting data from the included sources of evidence (e.g., calibrated forms or forms that have been tested by the team before their use, and whether data charting was done independently or in duplicate) and any processes for obtaining and confirming data from investigators. | |
| Data items | 11 | List and define all variables for which data were sought and any assumptions and simplifications made. | |
| Critical appraisal of individual sources of evidence§ | 12 | If done, provide a rationale for conducting a critical appraisal of included sources of evidence; describe the methods used and how this information was used in any data synthesis (if appropriate). | |
| Synthesis of results | 13 | Describe the methods of handling and summarizing the data that were charted. | |

St. Michael's
Inspired Care. Inspiring Science.

For peer review only - http://bmjopen.bmj.com/site/about/guidelines.xhtml

| SECTION | ITEM | PRISMA-ScR CHECKLIST ITEM | REPORTED ON PAGE # |
|---|---|---|---|
| **RESULTS** | | | |
| Selection of sources of evidence | 14 | Give numbers of sources of evidence screened, assessed for eligibility, and included in the review, with reasons for exclusions at each stage, ideally using a flow diagram. | |
| Characteristics of sources of evidence | 15 | For each source of evidence, present characteristics for which data were charted and provide the citations. | |
| Critical appraisal within sources of evidence | 16 | If done, present data on critical appraisal of included sources of evidence (see item 12). | |
| Results of individual sources of evidence | 17 | For each included source of evidence, present the relevant data that were charted that relate to the review questions and objectives. | |
| Synthesis of results | 18 | Summarize and/or present the charting results as they relate to the review questions and objectives. | |
| **DISCUSSION** | | | |
| Summary of evidence | 19 | Summarize the main results (including an overview of concepts, themes, and types of evidence available), link to the review questions and objectives, and consider the relevance to key groups. | |
| Limitations | 20 | Discuss the limitations of the scoping review process. | |
| Conclusions | 21 | Provide a general interpretation of the results with respect to the review questions and objectives, as well as potential implications and/or next steps. | |
| **FUNDING** | | | |
| Funding | 22 | Describe sources of funding for the included sources of evidence, as well as sources of funding for the scoping review. Describe the role of the funders of the scoping review. | |

JBI = Joanna Briggs Institute; PRISMA-ScR = Preferred Reporting Items for Systematic reviews and Meta-Analyses extension for Scoping Reviews.

* Where *sources of evidence* (see second footnote) are compiled from, such as bibliographic databases, social media platforms, and Web sites.

† A more inclusive/heterogeneous term used to account for the different types of evidence or data sources (e.g., quantitative and/or qualitative research, expert opinion, and policy documents) that may be eligible in a scoping review as opposed to only studies. This is not to be confused with *information sources* (see first footnote).

‡ The frameworks by Arksey and O'Malley (6) and Levac and colleagues (7) and the JBI guidance (4, 5) refer to the process of data extraction in a scoping review as data charting.

§ The process of systematically examining research evidence to assess its validity, results, and relevance before using it to inform a decision. This term is used for items 12 and 19 instead of "risk of bias" (which is more applicable to systematic reviews of interventions) to include and acknowledge the various sources of evidence that may be used in a scoping review (e.g., quantitative and/or qualitative research, expert opinion, and policy document).

*From:* Tricco AC, Lillie E, Zarin W, O'Brien KK, Colquhoun H, Levac D, et al. PRISMA Extension for Scoping Reviews (PRISMAScR): Checklist and Explanation. Ann Intern Med. 2018;169:467–473. doi: 10.7326/M18-0850.

St. Michael's
Inspired Care. Inspiring Science.



For peer review only - http://bmjopen.bmj.com/site/about/guidelines.xhtml

**Appendix B-** Search terms used within the literature search strategy.

Keywords for each subject heading of 'HIV' and 'patient-generated health data' respectively were combined using the search command 'OR'. Articles yielded within each of these searches were combined using the search command 'AND' to produce the final article results.

## Keywords used

***Subject heading: HIV***

HIV OR human immunodeficiency virus OR HIV/AIDS OR HIV-infected OR HIV-associated OR HIV-positive OR HIV positive OR HIV+ OR people living with HIV OR persons living with HIV OR HIV patient* OR HIV infection

***Subject heading: Patient-generated health data***

Patient generated data OR patient-generated data OR patient generated health data OR patient-generated health data OR patient generated health information OR patient-generated health information OR personal health record* OR patient portal* OR personal data sharing OR patient collected data OR patient-collected data OR personal health informati* OR personal health data OR electronic health record* OR electronic personal health record* OR electronic health data OR self-recorded health data OR self-recorded data OR self-generated data or self-reported data OR patient collated data OR patient-collected data OR patient-captured data OR patient-controlled data OR patient-reported data OR electronic record.

An example complete database search strategy, for Medline, is shown below:

For peer review only

| # | Searches | Results | Type | Actions | Annotations |
|---|---|---|---|---|---|
| 1 | exp HIV Infections/ or HIV/ or exp HIV Long-Term Survivors/ | 277245 | Advanced | Display Results More ▾ | |
| 2 | (HIV or human immunodeficiency virus).mp. or HIV/aids or HIV-infected.mp. or HIV-associated.mp. or HIV-positive.mp. or HIV positive.mp. or HIV+.mp. or people living with HIV.mp. or persons living with HIV.mp. or HIV patient*.mp. or HIV infection.mp. [mp=title, abstract, original title, name of substance word, subject heading word, floating sub-heading word, keyword heading word, organism supplementary concept word, protocol supplementary concept word, rare disease supplementary concept word, unique identifier, synonyms] | 351883 | Advanced | Display Results More ▾ | |
| 3 | exp health records, personal/ or exp patient generated health data/ or exp patient portals/ | 1636 | Advanced | Display Results More ▾ | |
| 4 | (Patient generated data or patient-generated data or patient generated health data or patient-generated health data or patient generated health information or patient-generated health information or personal health record* or patient portal* or personal data sharing or patient collected data or patient-collected data or personal health informati* or personal health data or electronic health record* or electronic personal health record* or electronic health data or self-recorded health data or self-recorded data or self-generated data or self-reported data or patient collated data or patient-collected data or patient-captured data or patient-controlled data or patient-reported data or electronic record).mp. [mp=title, abstract, original title, name of substance word, subject heading word, floating sub-heading word, keyword heading word, organism supplementary concept word, protocol supplementary concept word, rare disease supplementary concept word, unique identifier, synonyms] | 28979 | Advanced | Display Results More ▾ | |
| 5 | 1 or 2 | 391186 | Advanced | Display Results More ▾ | |
| 6 | 3 or 4 | 29523 | Advanced | Display Results More ▾ | |
| 7 | 5 and 6 | 489 | Advanced | Display Results More ▾ | |

## Appendix C- Data extraction table of included articles

| Studies concerning a hypothetical digital platform for PGData collection | | | | | | | |
|---|---|---|---|---|---|---|---|
| **Authors (date)** | **Location** | **Participants** | **Study design** | **Length of study** | **Study aims and summary** | **Digital platform summary** | **Measures** |
| Bussone (2018, Study One of three) | England, UK | N = 16 people living with HIV<br><br>Age: range 18-57 years, M = 39.87<br><br>Gender: 8 male, 8 female<br><br>Ethnicity: 1 White British, 1 White other, White/Black African, 8 Black African, 2 Latin, 1 Asian Pakistani, 1 White/Black/Caribbean, 1 Mixed other<br><br>Orientation: 5 homosexual, 9 heterosexual, 1 bisexual, 1 asexual | Qualitative, individual semi-structured interviews of approximately 30 minutes | N/A | • **Aim**: to understand how people living with HIV monitor their personal health data<br>**Topics addressed:** participants' current self-management behaviours and issues surrounding current, previous or desired means of tracking personal health information | N/A as no specific digital platform used | Verbal feedback from participants regarding self-tracking of their personal health information |

For peer review only

| | | | | | | | |
|---|---|---|---|---|---|---|---|
| Marent, Henwood & Darking (2018) | Brighton (UK), Lisbon (Portugal), Barcelona (Spain), Antwerp (Belgium) and Zagreb (Croatia) | N = 160<br>• 97 people living with HIV<br>• 63 HCPs working in HIV care<br>-40 doctors<br>-10 nurses<br>-4 psychologists<br>-4 pharmacists<br>-2 social workers<br>-2 nutritionists<br>-1 sexologist<br><br>Age (of people living with HIV): range = 23-78 years<br><br>Gender (people living with HIV): 78 male<br><br>Ethnicity: authors report that people living with HIV had 14 different nationalities, with 13 identifying as belonging to a migrant community<br><br>Orientation (people living with HIV): 65 gay or lesbian, 26 heterosexual, 3 bisexual, 3 other. | Qualitative, 14 recorded workshops and 22 semi-structured interviews | January-June 2016 | • **Aim**: to determine perceptions of, and advantages and concerns surrounding, the development of a mobile app for HIV management<br>• **Topics addressed:** perceived challenges and barriers to use of a mobile app for HIV management, current mobile app usage and perceived useful features of a mobile app for HIV management | A hypothetical mobile app was discussed. | Verbal feedback from participants regarding factors related to a hypothetical mobile app for HIV management |

For peer review only

| | | | | | | | |
|---|---|---|---|---|---|---|---|
| Nokes et al. (2013) | New York, USA | N = 100 people living with HIV/AIDS<br><br>Age: M= 48.5<br><br>Gender: 94 male<br><br>Orientation: unreported<br><br>Ethnicity: 7 Asian/Pacific Islander, 51 African American/Black, 34 Hispanic/Latino, 1 Native American, 4 White/Anglo, 3 other<br><br>Education: 21 <11th grade, 45 high school/general education development, 25 2-years college, 9 4+ years of college | Quantitative | N/A | • **Aim:** to assess self-efficacy of people living with HIV regarding use of a hypothetical personal health record<br>• Participants received an explanation of a personal health record and completed questionnaires measuring their self-efficacy in relation to digital and paper-based personal health records, condom use and chronic disease management | N/A as no specific digital platform used | • Self-efficacy for creation and use of a paper-based personal health record<br>• Self-efficacy for creation and use of a digital personal health record<br>• Self-efficacy regarding medication adherence (HIV-Treatment Adherence Self-Efficacy Scale)<br>• Self-efficacy for chronic disease management scale<br>Self-efficacy for condom usage scale |

For peer review only

| | | | | | | | |
|---|---|---|---|---|---|---|---|
| Ramanathan et al. (2013) | Los Angeles, USA | N = 29 people living with HIV<br><br>Age: range= 30-60 years<br><br>Gender: 75% male<br><br>-Ethnicity: reported approximately one third of participants in each ethnic group of African American, Latino and White<br><br>Orientation: 55% men who have sex with men, 10% transgender | Qualitative, focus group discussions | N/A | • **Aim:** to assess feature preferences for a mobile app for HIV self-management<br>• **Topics addressed:** privacy, goal-setting, data capturing methods, feedback regarding app-user behaviours and the role of reminders | A hypothetical mobile app was discussed. | • Verbal feedback regarding key issues pertaining to development of a mobile app for HIV self-management |

For peer review only

| | | | | | | | |
|---|---|---|---|---|---|---|---|
| Swendeman et al. (2016) | Los Angeles, USA | N = 12 HCPs from a Los Angeles County HIV clinic<br>• 3 doctors<br>• 1 nurse practitioner<br>• 2 nurse case managers<br>• 2 psychosocial case managers<br>• 1 psychosocial case manager<br>• 1 psychotherapist<br><br>Age: unreported<br><br>Gender: unreported<br><br>Ethnicity: unreported<br><br>Orientation: unreported<br><br>Education: unreported | Qualitative, individual 45-60 minute semi-structured interviews | N/A | • **Aim:** to assess HCP attitudes regarding a hypothetical mobile app (for people living with HIV) and online dashboards (for HCPs) for HIV management<br>• **Topics addressed:** usability, acceptability and barriers to digital platform use | A hypothetical mobile app and online dashboard were discussed. | • Verbal feedback from participants regarding the use of hypothetical web-based dashboards for HCPs and hypothetical mobile apps for people living with HIV, both to facilitate self-management in patients |

For peer review only

| **Studies concerning a novel digital platform for PGData collection** | | | | | | | |
|---|---|---|---|---|---|---|---|
| Schnall et al. (2011) | New York, USA | N = 37 Case Managers working with people living with HIV<br><br>Age: unreported<br><br>Gender: unreported<br><br>Ethnicity: unreported<br><br>Orientation: unreported | Qualitative, one-hour focus group discussions | March-December 2008 | • **Aim:** to determine factors influencing usage and acceptability of online "SelectHealth Continuity of Care Document" for people living with HIV, before the platform's release<br>• **Topics addressed:** facilitators and barriers to use of the continuity of care record | The "SelectHealth Continuity of Care Document" includes key health information including latest medical test results, pharmaceutical record, visits to healthcare facilities and patient contact information | • Verbal feedback regarding perceived facilitators and barriers to use of the "SelectHealth Continuity of Care Document" |
| Schnall et al. (2016) | New York, USA | N = 15<br>• 10 people living with HIV<br>• 5 informaticians<br><br>Age: unreported<br><br>Gender (people living with HIV): 6 male<br><br>Ethnicity: unreported<br><br>Orientation: unreported | Mixed: quantitative, qualitative | [illegible] | • **Aim:** to test a prototype mobile app designed to facilitate HIV management in people living with HIV Participants received a description of a mobile app prototype for HIV management<br>• Informaticians tested the model for 45-90 minutes and completed a Heuristic Evaluation Checklist<br>• people living with HIV evaluated prototype app screenings and completed the Post Study System Usability Questionnaire | No description of mobile app prototype provided | • For informaticians: score on Heuristic Evaluation Checklist<br>• For people living with HIV: score on Post Study System Usability Questionnaire as a measurement of the app's usability |

For peer review only

| | | | | | | | |
|---|---|---|---|---|---|---|---|
| Stonbraker et al. (2018) | New York, USA | N= 25<br>• 20 people living with HIV who possessed at least one HIV-associated non-AIDS condition<br>• 5 informaticians<br><br>Age: M= 54.4 years<br><br>Gender: 40% male<br><br>Ethnicity: only reported 85% African American<br><br>Orientation: unreported<br><br>Education: 50% <high school, 35% high school, 15% more than high school | Mixed: quantitative, qualitative | N/A | • **Aim:** to determine usability of novel mobile app *VIP-HANA* (Video Information Provider for HIV-associated non-AIDS conditions) designed to aid people living with HIV with management of HIV/non-HIV-related symptoms, and to determine where improvements to the app were needed<br>• After receiving descriptions of the app participants were assigned tasks on a beta version of the app and had to describe aloud their thoughts and actions whilst their activities were tracked<br>• people living with HIV completed one questionnaire to assess their health literacy level, and two questionnaires relating to the app's usability | • Within the app individuals must indicate which HIV-associated non-AIDS conditions they possess out of nine options<br>• Individuals can then categorise associated symptoms of the condition(s) out of 28 options, according to their severity<br>Upon severity categorisation, the app-user receives tailored strategies to facilitate self-management behaviours | • For informaticians: assigned mobile app task activity and score on Heuristic Evaluation Checklist<br>• For people living with HIV: assigned mobile app task activity, score on the Newest Vital Sign assessment (to determine health literacy level) and scores on two questionnaires relating to app usability (Health Information Technology Usability Evaluation Scale and Post Study System Usability Questionnaire) |

For peer review only

| **Studies concerning an existing, established digital platform for PGData collection** | | | | | | | |
|---|---|---|---|---|---|---|---|
| **Authors (date)** | **Location** | **Participants** | **Study design** | **Length of study** | • **Study aims and summary** | • **Digital platform summary** | • **Measures** |
| DeSilva et al. (2013) | Nanning, China | N= 10 people living with HIV who were injection drug users<br><br>Age: M = 32.7 years<br><br>Gender: 7 male<br><br>Orientation: unreported<br><br>Ethnicity: unreported<br><br>Education: 9 participants <middle school | Qualitative, individual interviews before and after using the device | One month | • **Aim:** to determine the feasibility and acceptability of using a *Wisepill* device in injecting drug users who were HIV positive<br>• Participants used the *Wisepill* device to monitor their antiretroviral medication adherence for one month<br>• **Topics addressed:** acceptability, feasibility and usability of the device | • The *Wisepill* container stores medication<br>• Upon opening, a wireless signal is immediately transmitted to a server, the data of which can be obtained by HCPs<br>• Opening the *Wisepill* container provides a proxy measurement of medication adherence | • Verbal feedback from participants regarding feasibility, acceptability and usability of the *Wisepill* device to track medication adherence<br>• Self-reported adherence to antiretroviral medication after one month of using the *Wisepill* device |

For peer review only

| | | | | | | | |
|---|---|---|---|---|---|---|---|
| Luque et al. (2013) | Rochester, New York, USA | N = 29 people living with HIV<br><br>Age: M= 48<br><br>Gender: 55% male, 41% female, 3% transgender<br><br>Ethnicity: 41% African American, 21% Latino, 28% white, 10% other<br><br>Orientation: unreported<br><br>Education level: 31% <high school, 28 % high school or equivalent, 41% >high school<br><br>Annual household income: 83% <$20,000, 17% >$20,000<br><br>Use of electronic technology: 18% never used a computer, 21% never used the internet, 36% never used email | Quantitative | 6 weeks | • **Aim:** to determine acceptability and usability of a personal health record on an iPod touch device, and to observe the effect of using a digital personal health record upon HIV treatment self-efficacy<br>• Participants underwent six 90-minute training sessions and were given 'homework' tasks to rehearse between training sessions | • The *MyMedical* app, (downloaded from iTunes Store) was trialled as a personal health record on an iPod touch device<br><br>• No more information regarding the app was provided within the study | • HIV-Treatment Adherence Self-Efficacy Scale (baseline and one month post-training)<br>• Post-training questionnaire score for iPod device usability<br>• Post-training questionnaire score for personal health record usability<br>• Attitudes Towards Computers Scale |

For peer review only

| | | | | | | | |
|---|---|---|---|---|---|---|---|
| Odlum et al. (2014) | New York, USA | N= 57 people living with HIV<br>• Quantitative: 41 people living with HIV<br>-M age= 47 years, range 24-63 years<br><br>-Gender: 27 male<br><br>-Ethnicity: 27 Black/African American, 11 other/mixed race<br><br>-Orientation: unreported<br><br>-Education: 25 <high school diploma, 4 associate degrees, 2 Bachelor's degrees<br><br>• Qualitative: 15 people living with HIV,<br>8 users of the *MyHealthProfile* app<br><br>-Gender: 2 male<br><br>-Ethnicity: 7 Black African American, 1 Latino<br><br>-Orientation: unreported | Mixed: quantitative (System Usefulness Survey), qualitative (focus groups of 1 hour) | N/A | • **Aim:** to determine usability of the *MyHealthProfile* app and to determine where improvements could be made to inform a new version of the app named *MyHealthProfile-plus*<br>• Participants completed 2 surveys relating to the usefulness and content of the pre-established *My Health Profile* app<br>• Participants completed 60 minute focus groups regarding the development of new *My Health Profile-plus* app | • The *MyHealthProfile* app acts as a continuity of care document for people living with HIV to track their personal health record via an internet connection | • System Usefulness Survey<br>• Ease of Use Scale<br>• App usefulness score<br>• App components score<br>• Verbal feedback regarding perceived app needs for novel app *My Health Profile-plus* |

For peer review only

| | | | | | | | |
|---|---|---|---|---|---|---|---|
| | | 7 non-users of the *MyHealthProfile* app<br>-Gender: 5 male<br>-Ethnicity: 6 Black African American, 1 Caucasian<br><br>-Orientation: unreported | | | | | |

For peer review only

## Appendix D- Results from the Mixed Methods Appraisal Tool assessment

**Bussone (2018)**

| Study design | Methodological quality criteria | Responses: Yes | No | Can't tell |
|---|---|---|---|---|
| **Screening questions (all studies)** | S1. Are there clear research questions? | X | | |
| | S2. Do the collected data allow to address the research questions? | X | | |
| **Qualitative** | 1.1. Is the qualitative approach appropriate to answer the research question? | X | | |
| | 1.2. Are the qualitative data collection methods adequate to address the research question? | X | | |
| | 1.3. Are the findings adequately derived from the data? | X | | |
| | 1.4. Is the interpretation of results sufficiently substantiated by data? | X | | |
| | 1.5. Is there coherence between qualitative data sources, collection, analysis and interpretation? | X | | |

For peer review only

<u>**DeSilva et al. (2013)**</u>

| | | Responses | | |
|---|---|---|---|---|
| **Study design** | **Methodological quality criteria** | **Yes** | **No** | **Can't tell** |
| **Screening questions (all studies)** | S1. Are there clear research questions? | X | | |
| | S2. Do the collected data allow to address the research questions? | X | | |
| **Qualitative** | 1.1. Is the qualitative approach appropriate to answer the research question? | X | | |
| | 1.2. Are the qualitative data collection methods adequate to address the research question? | X | | |
| | 1.3. Are the findings adequately derived from the data? | | | X |
| | 1.4. Is the interpretation of results sufficiently substantiated by data? | | | X |
| | 1.5. Is there coherence between qualitative data sources, collection, analysis and interpretation? | | | X |

For peer review only

**Luque et al. (2013)**

| Study design | Methodological quality criteria | Responses | | |
|---|---|---|---|---|
| | | **Yes** | **No** | **Can't tell** |
| **Screening questions (all studies)** | S1. Are there clear research questions? | X | | |
| | S2. Do the collected data allow to address the research questions? | X | | |
| **Quantitative descriptive** | 4.1. Is the sampling strategy relevant to address the research question? | X | | |
| | 4.2. Is the sample representative of the target population? | | X | |
| | 4.3. Are the measurements appropriate? | X | | |
| | 4.4. Is the risk of nonresponse bias low? | X | | |
| | 4.5. Is the statistical analysis appropriate to answer the research question? | X | | |

For peer review only

**Marent, Henwood & Darking (2018)**

| Study design | Methodological quality criteria | Responses: Yes | No | Can't tell |
|---|---|---|---|---|
| **Screening questions (all studies)** | S1. Are there clear research questions? | X | | |
| | S2. Do the collected data allow to address the research questions? | X | | |
| **Qualitative** | 1.1. Is the qualitative approach appropriate to answer the research question? | X | | |
| | 1.2. Are the qualitative data collection methods adequate to address the research question? | X | | |
| | 1.3. Are the findings adequately derived from the data? | X | | |
| | 1.4. Is the interpretation of results sufficiently substantiated by data? | X | | |
| | 1.5. Is there coherence between qualitative data sources, collection, analysis and interpretation? | X | | |

For peer review only

**Nokes et al. (2013)**

| | | Responses | | |
|---|---|---|---|---|
| **Study design** | **Methodological quality criteria** | **Yes** | **No** | **Can't tell** |
| **Screening questions (all studies)** | S1. Are there clear research questions? | X | | |
| | S2. Do the collected data allow to address the research questions? | X | | |
| **Quantitative descriptive** | 4.1. Is the sampling strategy relevant to address the research question? | X | | |
| | 4.2. Is the sample representative of the target population? | X | | |
| | 4.3. Are the measurements appropriate? | X | | |
| | 4.4. Is the risk of nonresponse bias low? | X | | |
| | 4.5. Is the statistical analysis appropriate to answer the research question? | X | | |

For peer review only

**Odlum et al. (2014)**

| | | Responses | | |
|---|---|---|---|---|
| **Study design** | **Methodological quality criteria** | **Yes** | **No** | **Can't tell** |
| **Screening questions (all studies)** | S1. Are there clear research questions? | X | | |
| | S2. Do the collected data allow to address the research questions? | X | | |
| **Qualitative** | 1.1. Is the qualitative approach appropriate to answer the research question? | X | | |
| | 1.2. Are the qualitative data collection methods adequate to address the research question? | X | | |
| | 1.3. Are the findings adequately derived from the data? | X | | |
| | 1.4. Is the interpretation of results sufficiently substantiated by data? | X | | |
| | 1.5. Is there coherence between qualitative data sources, collection, analysis and interpretation? | X | | |
| **Quantitative descriptive** | 4.1. Is the sampling strategy relevant to address the research question? | | | X |

| | | | | |
|---|---|---|---|---|
| | 4.2. Is the sample representative of the target population? | | X | |
| | 4.3. Are the measurements appropriate? | | | X |
| | 4.4. Is the risk of nonresponse bias low? | | | X |
| | 4.5. Is the statistical analysis appropriate to answer the research question? | | | X |
| **Mixed methods** | 5.1. Is there an adequate rationale for using a mixed methods design to address the research question? | | X | |
| | 5.2. Are the different components of the study effectively integrated to answer the research question? | | X | |
| | 5.3. Are the outputs of the integration of qualitative and quantitative components adequately interpreted? | | X | |
| | 5.4. Are divergences and inconsistencies between quantitative and qualitative results adequately addressed? | | X | |
| | 5.5. Do the different components of the study adhere to the quality criteria of each tradition of the methods involved? | | | X |

For peer review only

**Ramanathan et al. (2013)**

| | | Responses | | |
|---|---|---|---|---|
| **Study design** | **Methodological quality criteria** | **Yes** | **No** | **Can't tell** |
| **Screening questions (all studies)** | S1. Are there clear research questions? | X | | |
| | S2. Do the collected data allow to address the research questions? | X | | |
| **Qualitative** | 1.1. Is the qualitative approach appropriate to answer the research question? | X | | |
| | 1.2. Are the qualitative data collection methods adequate to address the research question? | X | | |
| | 1.3. Are the findings adequately derived from the data? | X | | |
| | 1.4. Is the interpretation of results sufficiently substantiated by data? | X | | |
| | 1.5. Is there coherence between qualitative data sources, collection, analysis and interpretation? | X | | |

For peer review only

**Swendeman et al. (2016)**

| | | Responses | | |
|---|---|---|---|---|
| **Study design** | **Methodological quality criteria** | **Yes** | **No** | **Can't tell** |
| **Screening questions (all studies)** | S1. Are there clear research questions? | X | | |
| | S2. Do the collected data allow to address the research questions? | X | | |
| **Qualitative** | 1.1. Is the qualitative approach appropriate to answer the research question? | X | | |
| | 1.2. Are the qualitative data collection methods adequate to address the research question? | X | | |
| | 1.3. Are the findings adequately derived from the data? | X | | |
| | 1.4. Is the interpretation of results sufficiently substantiated by data? | X | | |
| | 1.5. Is there coherence between qualitative data sources, collection, analysis and interpretation? | X | | |

For peer review only

**Schnall et al. (2011)**

| Study design | Methodological quality criteria | Responses: Yes | No | Can't tell |
|---|---|---|---|---|
| **Screening questions (all studies)** | S1. Are there clear research questions? | X | | |
| | S2. Do the collected data allow to address the research questions? | X | | |
| **Qualitative** | 1.1. Is the qualitative approach appropriate to answer the research question? | X | | |
| | 1.2. Are the qualitative data collection methods adequate to address the research question? | X | | |
| | 1.3. Are the findings adequately derived from the data? | X | | |
| | 1.4. Is the interpretation of results sufficiently substantiated by data? | X | | |
| | 1.5. Is there coherence between qualitative data sources, collection, analysis and interpretation? | X | | |

For peer review only

**Schnall et al. (2016)**

| | | Responses | | |
|---|---|---|---|---|
| **Study design** | **Methodological quality criteria** | **Yes** | **No** | **Can't tell** |
| **Screening questions (all studies)** | S1. Are there clear research questions? | X | | |
| | S2. Do the collected data allow to address the research questions? | | X | |
| **Qualitative** | 1.1. Is the qualitative approach appropriate to answer the research question? | | | X |
| | 1.2. Are the qualitative data collection methods adequate to address the research question? | | | X |
| | 1.3. Are the findings adequately derived from the data? | | | X |
| | 1.4. Is the interpretation of results sufficiently substantiated by data? | | | X |
| | 1.5. Is there coherence between qualitative data sources, collection, analysis and interpretation? | | | X |
| **Quantitative descriptive** | 4.1. Is the sampling strategy relevant to address the research question? | | X | |

For peer review only

| | | | | |
|---|---|---|---|---|
| | 4.2. Is the sample representative of the target population? | | X | |
| | 4.3. Are the measurements appropriate? | | | X |
| | 4.4. Is the risk of nonresponse bias low? | | | X |
| | 4.5. Is the statistical analysis appropriate to answer the research question? | | | N/A |
| **Mixed methods** | 5.1. Is there an adequate rationale for using a mixed methods design to address the research question? | | X | |
| | 5.2. Are the different components of the study effectively integrated to answer the research question? | | X | |
| | 5.3. Are the outputs of the integration of qualitative and quantitative components adequately interpreted? | | X | |
| | 5.4. Are divergences and inconsistencies between quantitative and qualitative results adequately addressed? | | X | |
| | 5.5. Do the different components of the study adhere to the quality criteria of each tradition of the methods involved? | | X | |

For peer review only

**Stonbraker et al. (2018)**

| | | Responses | | |
|---|---|---|---|---|
| **Study design** | **Methodological quality criteria** | **Yes** | **No** | **Can't tell** |
| **Screening questions (all studies)** | S1. Are there clear research questions? | X | | |
| | S2. Do the collected data allow to address the research questions? | X | | |
| **Qualitative** | 1.1. Is the qualitative approach appropriate to answer the research question? | | | X |
| | 1.2. Are the qualitative data collection methods adequate to address the research question? | | | X |
| | 1.3. Are the findings adequately derived from the data? | X | | |
| | 1.4. Is the interpretation of results sufficiently substantiated by data? | X | | |
| | 1.5. Is there coherence between qualitative data sources, collection, analysis and interpretation? | X | | |
| **Quantitative descriptive** | 4.1. Is the sampling strategy relevant to address the research question? | | | X |

For peer review only

| | | | | |
|---|---|---|---|---|
| | 4.2. Is the sample representative of the target population? | | X | |
| | 4.3. Are the measurements appropriate? | | | X |
| | 4.4. Is the risk of nonresponse bias low? | | | X |
| | 4.5. Is the statistical analysis appropriate to answer the research question? | | | X |
| **Mixed methods** | 5.1. Is there an adequate rationale for using a mixed methods design to address the research question? | | X | |
| | 5.2. Are the different components of the study effectively integrated to answer the research question? | | X | |
| | 5.3. Are the outputs of the integration of qualitative and quantitative components adequately interpreted? | | X | |
| | 5.4. Are divergences and inconsistencies between quantitative and qualitative results adequately addressed? | | X | |
| | 5.5. Do the different components of the study adhere to the quality criteria of each tradition of the methods involved? | | X | |

For peer review only
